# Supporting the working life exposome: Annotating occupational exposure for enhanced literature search

**DOI:** 10.1371/journal.pone.0307844

**Published:** 2024-08-15

**Authors:** Paul Thompson, Sophia Ananiadou, Ioannis Basinas, Bendik C. Brinchmann, Christine Cramer, Karen S. Galea, Calvin Ge, Panagiotis Georgiadis, Jorunn Kirkeleit, Eelco Kuijpers, Nhung Nguyen, Roberto Nuñez, Vivi Schlünssen, Zara Ann Stokholm, Evana Amir Taher, Håkan Tinnerberg, Martie Van Tongeren, Qianqian Xie

**Affiliations:** 1 Department of Computer Science, National Centre for Text Mining, University of Manchester, Manchester, United Kingdom; 2 Centre for Occupational and Environmental Health, School of Health Sciences, University of Manchester, Manchester, United Kingdom; 3 Federation of Norwegian Industries, Oslo, Norway; 4 Department of Occupational Medicine and Epidemiology, National Institute of Occupational Health, Oslo, Norway; 5 Department of Public Health, Research Unit for Environment, Occupation and Health, Danish Ramazzini Centre, Aarhus University, Aarhus, Denmark; 6 Department of Occupational Medicine, Danish Ramazzini Centre, Aarhus University Hospital, Aarhus, Denmark; 7 Institute of Occupational Medicine, Edinburgh, United Kingdom; 8 Netherlands Organisation for Applied Scientific Research, Utrecht, Netherlands; 9 Department of Global Public Health and Primary Care, University of Bergen, Bergen, Norway; 10 Occupational Health Group, Institute for Risk Assessment Sciences, Utrecht University, Utrecht, Netherlands; 11 Center for Occupational and Environmental Medicine, Stockholm, Sweden; 12 School of Public Health and Community Medicine, University of Gothenburg, Gothenburg, Sweden; 13 Institute of Environmental Medicine, Karolinska Institutet, Stockholm, Sweden; University of Kurdistan Hewler, IRAQ

## Abstract

An individual’s likelihood of developing non-communicable diseases is often influenced by the types, intensities and duration of exposures at work. Job exposure matrices provide exposure estimates associated with different occupations. However, due to their time-consuming expert curation process, job exposure matrices currently cover only a subset of possible workplace exposures and may not be regularly updated. Scientific literature articles describing exposure studies provide important supporting evidence for developing and updating job exposure matrices, since they report on exposures in a variety of occupational scenarios. However, the constant growth of scientific literature is increasing the challenges of efficiently identifying relevant articles and important content within them. Natural language processing methods emulate the human process of reading and understanding texts, but in a fraction of the time. Such methods can increase the efficiency of both finding relevant documents and pinpointing specific information within them, which could streamline the process of developing and updating job exposure matrices. Named entity recognition is a fundamental natural language processing method for language understanding, which automatically identifies mentions of domain-specific concepts (*named entities*) in documents, e.g., exposures, occupations and job tasks. State-of-the-art machine learning models typically use evidence from an *annotated corpus*, i.e., a set of documents in which named entities are manually marked up (*annotated*) by experts, to learn how to detect named entities automatically in new documents. We have developed a novel annotated corpus of scientific articles to support machine learning based named entity recognition relevant to occupational substance exposures. Through incremental refinements to the annotation process, we demonstrate that expert annotators can attain high levels of agreement, and that the corpus can be used to train high-performance named entity recognition models. The corpus thus constitutes an important foundation for the wider development of natural language processing tools to support the study of occupational exposures.

## Introduction

Occupational exposures constitute major contributors towards non-communicable diseases [1]. On a global level, it is estimated that occupation-related diseases are responsible for between 3% and 7% of deaths annually [2–4]. Although associations between numerous occupational exposures and diseases have been well studied, the focus is often on links between a single type of exposure and a specific health outcome. There is also typically an assumption that all workers with a particular job title are subject to the same types and levels of exposures [5]. However, this approach is overly simplistic for several reasons. Firstly, people working in a given occupation will almost always be subject to *multiple* exposures, whose types and intensities may vary according to the specific characteristics of their employment. These characteristics include the types of tasks that they undertake [6] and the environment in which they work [7]. Secondly, over the course of their working lives, it is common for people to be employed in a variety of jobs, each of which may be subject to different types of exposures. Thirdly, over time, various factors can result in alterations in exposures and their levels. These include changes in work environment and process conditions; introduction of new standards; and response to regulatory requirements [8–10]. These factors, in combination with the varying influences of non-occupational exposures that result from differing lifestyles, make it highly likely that each person with a particular job title will have experienced different types and levels of exposures over the course of their lifetime. Such differences could significantly impact upon their individual likelihoods of developing specific non-communicable diseases [11].

Compared to the traditional “single exposure, single disease” approach to studying exposure-disease associations, the *working-life exposome* approach [5] is better aligned to the realities of workers’ lives, since it considers how the *totality* of occupational and non-occupational exposures experienced by individuals during their lifetime impacts upon their health.

*Job Exposure Matrices (JEMs)* are structured resources that can support the working-life exposome approach, through their cross-tabulation of occupations with estimated indices of exposure to one or more factors [12,13]. The use of JEMs makes it feasible to discover the types and levels of exposures experienced by individuals over the course of their working lives, based on their job histories. Nevertheless, JEMs exhibit certain limitations. For example, the time and expense required to collect and analyse data to determine exposure estimates [14] necessarily restricts the current inventory of JEMs to a subset of all possible occupational exposures. Moreover, changes in the occupational landscape [15], triggered by variety of societal, economic, technological, legal and political factors [16], can lead to altered exposure levels and/or the set of occupations that are subject to a specific type of exposure. The frequency of such changes can make it challenging to ensure that existing JEMs are kept up-to date and accurate. Furthermore, the occupational-level exposure estimates provided in most existing JEMs may not be sufficiently granular to account for potential exposure variations within the same job category. These can occur due to different working conditions, such as specific tasks undertaken or environmental conditions [17].

Scientific literature articles constitute a primary source of evidence to support the development and update of JEMs [18,19], since they often contain detailed information regarding exposures in a wide variety of occupational scenarios. However, locating relevant articles among the vast and constantly growing repository of published scientific literature represents a significant challenge. Keyword queries almost inevitably retrieve a significant proportion of irrelevant articles, meaning that searches must typically be followed by a detailed screening of the retrieved articles to verify their relevance and to locate important information within them.

Natural Language Processing (NLP) techniques [20] emulate certain parts of the human process of reading and understanding texts, but in a fraction of the time. Applying NLP methods to large document collections can thus provide more effective and efficient means of locating of relevant articles and pinpointing relevant information within them. This in turn could help to accelerate the JEM development process, increase the feasibility of refining them with more fine-grained exposure estimates, and facilitate easier tracking of temporal developments that may require JEMs to be updated. However, a recent literature review of NLP methods concerning exposure assessment [21] revealed that the small number of existing approaches focused on *occupational* exposures (e.g., [22–24]) are currently insufficient to support detailed automated analysis of documents in this field.

As a step towards stimulating further NLP research in this area, this article focuses on Named Entity Recognition (NER) [25] for occupational exposures. NER plays a fundamental role in automated language understanding, and is a prerequisite for various, more complex NLP methods. The goal of NER is to automatically identify and categorise mentions of important domain-specific concepts (known as *named entities* or *NEs*) in documents. Most state-of-the-art NER approaches use machine learning (ML), or more specifically, deep learning (DL) methods [26]. The learning process is generally reliant on an *annotated corpus* of domain-specific documents, in which NEs of interest have been manually marked-up by experts in the field. Although a domain-relevant annotated corpus concerning physical exposures/accidents and their consequences in the construction industry has previously been developed [27], the production of additional corpora annotated with NEs relevant to other types of occupational exposures is key to broadening the scope of NLP efforts within this field.

In response, we have developed a novel corpus annotated with NEs relevant to substance exposures. The corpus consists of pertinent sections of selected scientific literature articles, in which domain experts have annotated six different categories of NEs that are particularly relevant in the context of developing, refining and updating JEMs. The NE categories encompass the exposure substances themselves, the circumstances under which these exposures occur (e.g., jobs/tasks undertaken, working environments, etc.) and information about how exposure measurements were taken. We show that the average levels of inter-annotator agreement (IAA) across our corpus compare with or exceed related efforts, thus providing evidence of the quality and consistency of the annotations produced. We furthermore demonstrate the practical value of our corpus by using it to fine-tune and evaluate two state-of-the-art ML-based NER models, with highly promising results. It is intended that the corpus and initial NER results will act as a stimulus for the development of further novel NER approaches and of more sophisticated NLP methods that exploit the NEs as the basis to recognise more complex knowledge in articles.

The remainder of this article is structured as follows. In the *Related work* section, we provide a survey of previous NLP work in the field of exposure assessment. Subsequently, in the *Materials and methods* section, we introduce the iterative workflow used to develop our corpus, describe the annotation scheme, and provide an overview of the NER methods that we have applied to the final corpus to demonstrate its suitability for ML purposes. In the *Results* section, we firstly report and analyse various statistics relating to IAA and individual annotator behaviour over the various iterations of the corpus development workflow. We then present and discuss the statistics and characteristics of the final annotated corpus. Subsequently, we report and discuss the results of our NER experiments. Finally, in the *Conclusion* section, we summarise our contributions and suggest directions for future work.

## Related work

Although there is a scarcity of NLP research concerned specifically with *occupational* exposures, NLP techniques have been more widely applied to the study of other types of exposures. Our review of related work thus covers a range of exposure types, to provide a general demonstration of the utility of NLP analyses within the exposure assessment field. Firstly, we investigate the important contribution of NER to different types of NLP analyses and NLP-based applications, with a focus on exposure assessment. Secondly, we survey a range of previous exposure-related NER approaches, and consider their relative advantages and disadvantages.

### Applications of named entity recognition in exposure assessment

The output of NER tools has been demonstrated as a useful aid for various tasks in the field of exposure assessment, e.g., systematic reviewing [28] and exposure database curation [29]. NEs can help both to identify documents relevant to these tasks, and to locate important information within the documents. The efficiency of finding specific information within documents can be enhanced through the application of relation extraction methods [30]. Relations constitute important “nuggets” of information (i.e., brief, self-contained information items [31]) in text that involve NEs. Automated recognition of relations can make it possible to rapidly locate documents containing specific types of evidence, e.g., exposures contributing towards metabolic syndrome [32] or causal relations between microbial exposures and diseases [33]. Relations can also form the basis for the development of *knowledge graphs* (KGs) [34], whose structured representations of the knowledge conveyed in collections of documents can be straightforwardly explored and/or queried to alleviate the need for detailed reading of large numbers of documents. Within the domain of occupational exposures, a KG encoding the circumstances of physical exposures/accidents in the construction industry [35] was developed using the output of an NER model [36] trained using the previously introduced annotated corpus [27].

NEs can also feed into other types of NLP methods to improve their performance. An example is automatic document classification [37], which may be used to categorise documents according to the type(s) of exposure that they describe [38] or to determine whether or not a document is relevant to a subject of interest, e.g., the effects of environmental exposures on human health [39,40]. Topic modelling methods [41], which automatically identify the range of subjects or topics discussed within a collection of documents, can also be guided by domain-specific NEs [42]. The output of topic modelling methods can be used as the basis for *clustering* documents into thematically similar groups [43], which can help to quickly identify the most relevant documents returned by a search. In terms of exposures, topic modelling has been used to characterise the landscape of the exposome research field [44] and to identify topics in articles concerning climate-related [45] and early life [46] exposures.

The practical utility of NLP methods such as those introduced above has been further demonstrated through their integration within a range of user-oriented web-based applications. These include tools to increase the efficiency of screening for systematic reviewing [47,48] and semantic search systems. The latter allow users to flexibly explore and rapidly filter documents based on various aspects of their content, such as mentions of diseases, genes and proteins in COVID-19 research papers [49], and entities and relations concerning the circumstances of accidents/physical exposures in workplace accident reports [50].

### Approaches to exposure-related NER

Several different approaches have been taken to exposure-related NER. Depending on the exposures of interest, it may be sufficient to reuse existing biomedical tools [51–54] to recognise NEs such as chemicals, genes, proteins and diseases [32,55]. Novel approaches to recognising other types of exposures have included pattern matching, which can be feasible for NEs with a fairly fixed format, e.g., sources of exposure to electromagnetic fields [56]. A more popular approach involves detecting NEs by matching phrases occurring in documents against a dictionary that lists important domain-specific concepts. For example, a dictionary generated from concepts in the EXPOSEO ontology [57] was used to detect environmental exposures to nanomaterials [58], while chemicals and their associated synonyms in the PubChem database [59] were used as the basis to detect chemicals found in blood [29]. In other cases, task-specific dictionaries were developed to recognise safety risk factors in accident reports [23], fault-related exposures [28], asthma-triggering exposures in tweets [60] and socioeconomic status exposures in electronic health records [61]. However, the highly variable nature of language means that dictionary-based approaches must account for the potentially many synonymous ways in which concepts may be mentioned in text. Accordingly, manual development of custom dictionaries is only realistically feasible when the scope of the concepts to be recognised is restricted. For example, although promising results were achieved using a dictionary-based approach to detect six key types of information about epidemiological studies in academic abstracts [62,63], the considerable manual effort required to develop multiple dictionaries limits the scalability/adaptivity of such an approach. Moreover, although several large-scale resources provide inventories of concepts relevant to occupational exposure assessment (e.g., job titles and industries [64–67]), their lack of detailed synonym lists restricts their ability to support NER for occupational exposures.

Approaches based on ML are generally more promising and scalable. Their ability to learn general patterns in text that denote the presence of NEs, using evidence from an annotated corpus, allows them to recognise a wider range of NEs, compared to dictionary-based methods. However, it can be challenging to produce an annotated corpus that is sufficiently representative of real data to allow ML models to learn and generalise effectively. Although increasing the size of the corpus could help to achieve adequate representativeness, the time-consuming nature of producing even modest amounts of high-quality human-annotated data can make this infeasible. State-of-the-art deep learning approaches [68] can help to mitigate this issue by exploiting the complex language understanding capabilities of pre-trained language models (PLMs), such as Bidirectional Encoder Representations from Transformers (BERT) [69]. PLMs are unsupervised language models that have undergone pre-training with an extensive amount of data, enabling them to capture lexical, syntactic and semantic knowledge. This detailed linguistic knowledge can then be exploited for various NLP tasks, such as NER, sentiment analysis and question answering, by *fine-tuning* the PLM with the aid of an annotated corpus. Fine-tuned PLM-based models can sometimes exceed human levels of performance, even using modest amounts of annotated data [69].

Several previously developed PLM-based models can recognise a subset of NE types relevant to occupational exposures, such as occupation names [70,71] and chemicals [72–74]. However, these models were fine-tuned using corpora belonging to various different domains and/or text genres (e.g., tweets, electronic health records and scientific articles). The characteristics of the corpus used for fine-tuning can affect the performance of the model when it is applied to documents belonging to other domains/genres [26,75]. For example, a model that is fine-tuned using tweets, which are typically terse, and often use language that is colloquial and ungrammatical [76], is likely to struggle if applied to the more formal, descriptive language of literature articles.

Our construction of a corpus in which multiple NE types relevant to occupational exposures are all annotated in domain-relevant literature articles is thus aimed at facilitating the development of NER models that can recognise a range of important domain-specific concept mentions, and which can perform optimally when applied to new documents with similar characteristics.

## Materials and methods

### Annotation workflow

Our corpus development workflow closely follows the *Model-Annotate-Model-Annotate (MAMA)* cycle [77], which consists of four steps that are followed in an iterative manner:

- **Model**–An annotation *model* or scheme defines the set of NE categories to be annotated, accompanied by a set of *guidelines* to instruct annotators on how and when to mark-up mentions of each category.
- **Annotate**—Annotators follow the guidelines to mark up a set of documents.
- **Evaluate–**IAA is calculated to determine the extent to which different annotators can follow the guidelines to produce consistent (and thus, high quality) annotations. Consistency is important to facilitate successful training/fine-tuning of ML models [26].
- **Revise—**Annotation discrepancies are identified and discussed with annotators, as a result of which the scheme and/or guidelines may be revised to improve their robustness and clarity, prior to the next iteration of the cycle.

Our initial scheme and guidelines were developed by analysing a sample of articles concerning substance exposures of interest. Collaborative analysis between experts in occupational exposures and NLP aimed to ensure both the identification of the most pertinent domain-specific concepts typically mentioned within articles and the suitability of the NE annotations produced for ML purposes. The annotation was carried out by twelve annotators, all of whom have hands-on knowledge of exposure assessment. Following guidance in [78], each article was annotated by at least two of these annotators, with the aid of the *brat* annotation tool [79].

We applied the MAMA cycle in four iterations or “rounds”, which vary in terms of the number of articles annotated and/or the focused exposure substance discussed in the articles, as detailed further below. The final set of guidelines is provided in S1 Appendix. To create the final corpus, we combined the annotations from different annotators into unified sets by employing a rule-based method (described in S2 Appendix), which takes into account the strengths and weaknesses of individual annotators in annotating different NE categories.

We follow [80] by annotating NEs in both the abstract and full-text sections of each article. Although there is likely to be some overlap in their information content, abstracts and full paper sections have been shown to vary in terms of their language structure [81]. Therefore, annotating different parts of articles can provide the evidence needed to develop NER models that can perform robustly across different sections, regardless of their specific language structure. This can be useful in facilitating the automated detection of information at varying levels of granularity. For example, high-level details of a study may be extracted from the abstract, while more detailed information may be extracted from full-text sections. To maximise the amount of useful information annotated, only selected sections from the full text of each article were annotated (i.e., *Methods* and *Results*), since these sections typically contain the densest and most relevant information pertaining to exposure measurements.

### Annotation scheme

Table 1 provides information about the six different NE categories that constitute our final annotation scheme, along with short definitions and examples.

**Table 1:** Annotation scheme for substance exposures.

Category	Definition	Examples
Substance or Exposure Measured	Names of substances, chemicals or pollutants (recognised exposure entities) that are measured or sampled.	*respirable quartz dust; elemental carbon*
Occupation/Job Title	Phrases that characterise a person or group of people who form the subject(s) of exposure studies. The characterisation may be in terms of their occupation, job title, position, general type of work undertaken, types of equipment/materials typically worked with, or their working location/environment.	*carpenters; concrete workers; operators in the refinery*
Industry/Workplace	Phrases denoting either the type of workplace OR the industry involved in the sampling series.	*mining operations; trucking industry; diesel factory; our-lane motorway*,*; diesel powered fork-lift trucks*
Job Task/Activity	Specific and well-defined physical activities or actions that are carried out by workers as part of their daily working duties.	*welding; concrete pouring; mechanical mowing of weeds*
Occupational Hygiene (OH) Measurement Device	Phrases that name or describe the characteristics of a device, tool, apparatus, or sampling head used by occupational hygienists to measure levels of particulate and gaseous exposures in the workplace.	*IOM samplers; Higgins Dewell cyclones; 25 mm closed-faced aerosol filter cassettes; Dräger stain tubes*
Sample Type Personal	Phrases denoting that collected samples of airborne substances, chemicals or pollutants represent personal exposures.	*personal measurements; personal breathing zone sample*

*Substance or Exposure Measured* and *Occupation/Job Title* are fundamental categories, since they correspond to the main axes in most substance-oriented general population JEMs. Their automated recognition could facilitate exploration of the range of jobs that are mentioned in the context of substance exposure(s) of interest and allow the straightforward location of articles that are likely to provide evidence relating to exposed occupations. *Industry/Workplace* and *Job Task/Activity* aim to capture fine-grained information about working circumstances that could influence the specific exposures and/or intensities experienced by workers with a particular job title. The ability to explore how exposures differ by industry, workplaces and job tasks within the context of particular occupations across a range of different articles could help to suggest novel and more appropriate ways of classifying exposure estimates, compared to the more standard use of job titles alone.

Determining *how* exposure measurements were performed is important when developing JEMs. Ideally, the studies on which exposure estimates are based should all use the same techniques, to minimise potential variability of the results. This motivates the annotation of the *OH Measurement Devices* used to collect samples, along with evidence that the *Sample Type* was personal (i.e., the sampler was carried by individuals in the breathing zone). While samples may either be personal or static/stationary, we only annotate evidence of personal sampling. This is partly because evidence of personal sampling is typically described more clearly and explicitly in articles, compared to evidence of stationary sampling. Additionally, personal sampling is more relevant for JEM development, since it directly measures the exposure levels experienced by workers, and is used as the basis for comparison with exposure limits. When automatically recognised NEs are eventually used to aid with literature search, an absence of *Sample Type Personal* NEs in an article is likely to indicate that stationary sampling was undertaken.

### Annotation rounds

Details regarding the four different rounds of annotation are as follows:

- **Round 1—**A single article concerning diesel exhaust exposures was independently marked up by all 12 annotators as an initial test of the scheme and guidelines. Given that this round used the first version of the guidelines, and it was the annotators’ first attempt to perform the annotation task, it was anticipated that the annotations produced would not be of a sufficient quality/consistency to be included in our final corpus. However, the types of disagreements identified would provide valuable evidence about issues with the initial scheme and guidelines.
- **Round 2 –**A total of 51 articles and reports that include measurements of occupational diesel exhaust exposures were annotated. To ensure the selection of articles containing details pertinent to the development and update of JEMs, they were chosen from the sets of publications identified by two literature reviews concerning occupational diesel exhaust exposures [82,83]; the measurement data from articles identified in one of these reviews was used as basis to develop the diesel engine exhaust JEM (DEE-JEM) [82]. The selected articles were split into six different groups of roughly equal sizes, each of which was independently marked up by two randomly paired annotators.
- **Round 3 –**Three articles, each concerning exposure measurements of respirable crystalline silica (RCS) in a different industry, i.e., agriculture, construction and silicon carbide, were marked up by all twelve annotators. According to the substantial updates to the guidelines made at the end of round 2, along with the change to a different exposure substance, we decided to carry out this common exercise involving all annotators. This would allow us to assess the potential impact of these changes on annotation quality, prior to carrying out larger scale annotation of articles concerning RCS exposures.
- **Round 4 –**A total of 47 articles and reports were annotated, each describing measurement series of RCS exposures in different industries. Similarly to round 2, some of these publications were selected from the sets identified in previous literature reviews focused on RCS exposures in two different industries, i.e., construction [84] and agriculture [85]. However, to ensure that the corpus provides evidence of descriptions of RCS exposures across different industries, we selected additional articles concerning other industries that were retrieved using the following PubMed query: ((((silica) OR (quartz)) AND (exposure)) AND ((worker*) OR (occupational))) AND (measurement*). Similarly to round 2, the articles were split into six different groups, each marked up by two annotators. In this round, however, annotators were paired *selectively*, as described below, to try to maximise the quality and consistency of the annotations produced.

To determine the most appropriate selective pairing of annotators in round 4, the twelve annotators were ranked according to their average pairwise IAA scores in round 3. The six annotators with the highest scores were placed in the *primary* group, while the remaining six annotators were placed in the *secondary* group. Each article was annotated by one annotator from each group. It was anticipated that the primary annotators would produce a strong set of annotations for each document. Based on an analysis of the annotation trends for each annotation pair, rules were then formulated (see S2 Appendix) to determine whether and how these primary annotations should be augmented with those of the secondary annotator. To avoid any potential influence on annotation behaviour, annotators were unaware of these groupings and of their relative performance in comparison to other annotators. However, all annotators were provided with individual, targeted feedback prior to round 4, in an attempt to maximise the correctness of their annotations.

### NER experiments

The final corpus was used to fine-tune and evaluate two BERT-based NER models, both of which have previously demonstrated high levels of performance when applied to the task of detecting NEs in scientific articles (e.g., [86,87]). Both models use the Hugging Face ML and data science platform [88] in combination with the PyTorch deep learning library [89]. The characteristics of the two different models are as follows:

- **Token-based model–**Assigns labels to each token (i.e., word) in a sentence, according to whether the token constitutes:
  ○ The *beginning (B)* of an NE
  ○ A word *inside (I)* of a multi-word NE
  ○ The *end* (*E)* of a multi-word NE
  ○ A word *outside (O)* of an NE

An example of a sentence with expected labels for each token is shown in Table 2.

**Table 2:** Example of a sentence with token-level NE labels.

The	primary	activity	at	both	garages	was	Diesel	engine	maintenance	.
**O**	**O**	**O**	**O**	**O**	**B-Industry** **Workplace**	**O**	**B-JobTask** **Activity**	**I-JobTask** **Activity**	**E-JobTask** **Activity**	**O**

- **Span-based model-** Generates all possible NE spans in a sentence, consisting of different numbers of tokens, and assigns a label to each span according to its most appropriate NE type (if any). The advantage of span-based models is that they are able to learn rich representations of the complete NE spans, rather than learning only representations of individual tokens [90]. We employ the recently-proposed span-based approach described in [91].

To ensure a thorough evaluation of the suitability and robustness of each of the models, we performed the following experiments:

- **10-fold cross validation–** 90% of the sentences in the corpus were split randomly into ten equal groups or folds. Ten different experiments were performed, by taking each fold in turn as the test set, and combining the remaining nine folds as the training set.
- **Evaluation on held-out test set—**To further assess the performance and robustness of the models when applied to previously unseen data, they were evaluated on the *held-out test set*, consisting of the remaining 10% of the corpus, which was not used at all during the cross-validation experiments. Models were trained using a random 80% of the corpus, while a further 10% was used as the development set, to fine-tune model parameters.

## Results and discussion

### Average inter-annotator agreement

**Fig 1:**
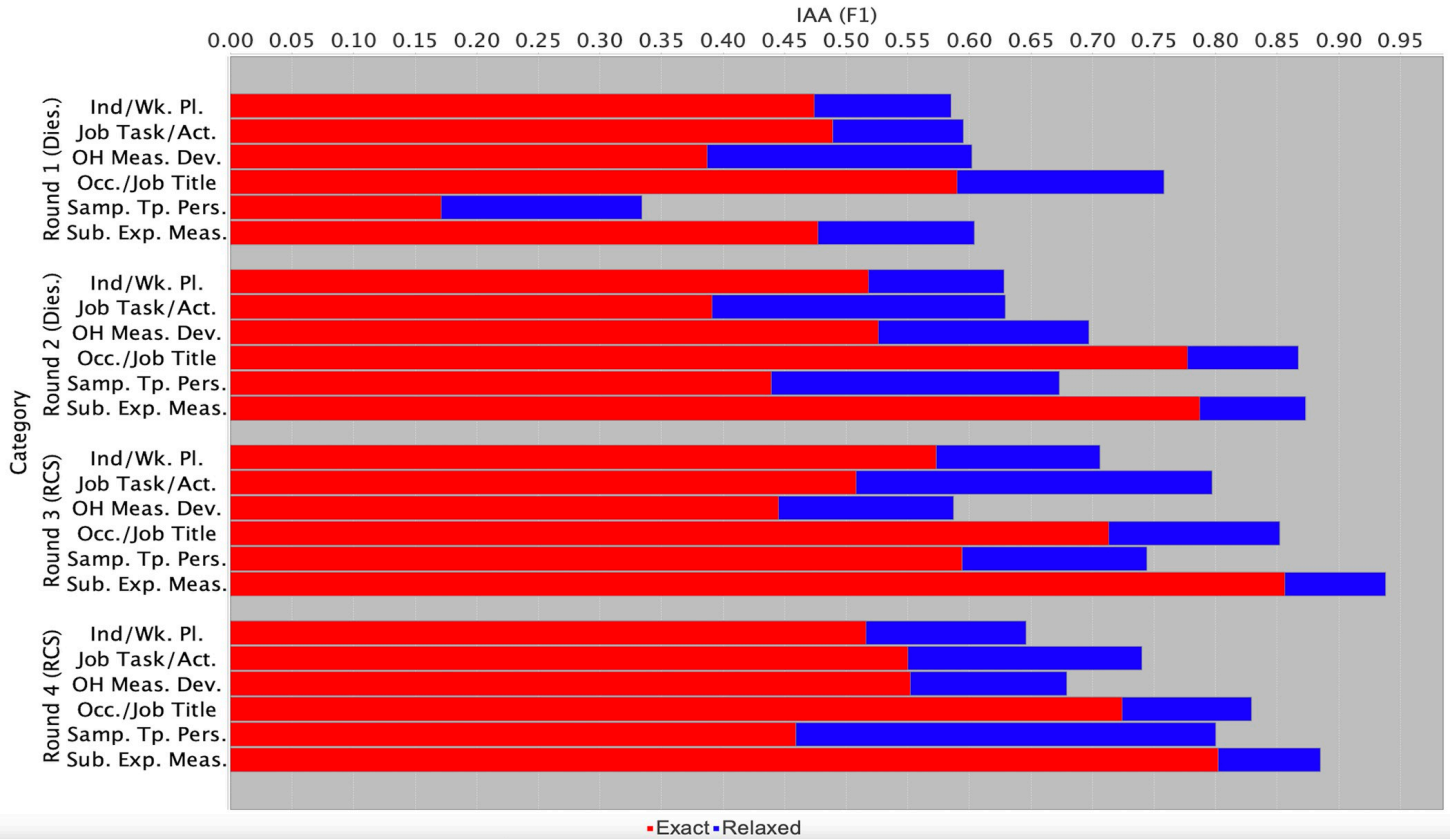
Average IAA rates across different annotation rounds. “Ind/Wk. Pl” = Industry/Workplace; “Job Task/Act.” = Job Task/Activity; “OH Meas. Dev.” = OH Measurement Device; “Occ./Job Title” = Occupation/Job Title; “Samp. Tp. Pers” = Sample Type Personal; “Sub. Exp. Meas” = Substance or Exposure Measured; “Dies.” = Diesel Exhaust.

Fig 1 illustrates the average IAA rates attained by different pairs of annotators for each NE category across each of the four annotation rounds. IAA was calculated in terms of the F1 score [92]. We report results for both *exact matching* (where the category and span of annotations created by two different annotators must match exactly) and *relaxed matching* (where it is sufficient for the two annotators’ spans to overlap with each other, as long as the same category has been assigned).

Between rounds 1 and 3, there was a general trend for IAA to increase, thus demonstrating that the iterative workflow helped to improve the quality and consistency of annotations. The tendency for IAA to drop for most categories in round 4 can be explained by our above-mentioned strategy of pairing annotators from the primary and secondary groups in this round. While this aimed to ensure that each article had at least one set of strong annotations (as discussed further below), it was also likely to result in the lowest IAA rates. Nevertheless, the magnitude of most IAA reductions in round 4 was small, while IAA still increased for some categories. These observations provide evidence that additional guideline refinements prior to round 4 helped to maintain or further improve annotation quality.

According to [92], an F1 score of below 0.60 for IAA is indicative of a complex annotation task and/or a lack of sufficient guidance about how to perform the task. Despite the lower IAA in round 4 compared to round 3, average relaxed IAA rates for all categories in round 4 still reached above this 0.60 F1 threshold, ranging between 0.65 F1 (*Industry/Workplace*) and 0.89 F1 (*Substance or Exposure Measured*). These IAA rates compare favourably with those obtained for the related task of annotating the same number (but different types) of NE categories relating to occupational hazards/exposures in the construction industry, for which relaxed IAA ranged between 0.68 and 0.92 F1 [27]. These annotations were shown to be adequate for training an NER tool suitable for practical integration in a semantic search system [50]. Annotation efforts in other domains with similar numbers of NE categories report similar IAA results (e.g. [93,94]), and also demonstrate the suitability of their annotations for training NER models. Such comparisons reinforce the adequacy of our annotated corpus for training ML-based NER tools.

Below, we provide brief discussions regarding the IAA patterns observable in Fig 1 for the six different NE categories. More detailed discussion is provided in S3 Appendix.

**Occupation/Job Title–**The consistently high levels of IAA obtained may be explained by a combination of factors. These include the existing familiarity of most annotators with phrases denoting occupations, the restricted semantic scope of the category in comparison to several other categories, and the relatively fixed nature of the types of spans to be annotated. The latter typically correspond to simple noun phrases consisting of one or more nouns and adjectives (e.g., *train driver*, *construction workers*, etc).

**Substance or Exposure Measured–**Low IAA in round 1 largely concerned disagreements involving a category that was removed from the original annotation scheme, i.e., *Exposure Form*. Information about state or form is often expressed as an adjective (e.g., *particulate* or *gaseous*) within the same noun phrase as the named substance. However, since a general guideline states that annotations should usually correspond to *complete* noun phrases, there was confusion about how to annotate phrases containing *both* a form *and* a substance, e.g., *gaseous PAHs*. We thus combined the two categories, such that information about state or form occurring within the immediate context of an exposure substance should be included within the span of the *Substance or Exposure Measured* annotation. Following this change, IAA increased considerably in subsequent rounds.

**OH Measurement Device—**IAA increased between rounds 1 and 2, following extended exemplification in the guidelines of the different types of apparatus/equipment that should be annotated (e.g., sampling heads, cassettes and other filter holders, containers for collecting gaseous samples and devices used to collect real time samples) and clarification of the details that should be included within annotated spans. Such details include manufacturer names (e.g., *Grimm* *PDM*), size information (e.g., *10-mm* *Dorr-Oliver cyclone*) and material of manufacture (e.g., *aluminum* *47-mm in-line filter holders*). The unstable IAA observable in rounds 3 and 4 may at least partly be explained by annotators’ varying levels of familiarity with occupational hygiene equipment. This sometimes led to difficulties in differentiating pieces of equipment/apparatus used to measure levels of exposures in the workplace from those used to perform laboratory analysis (e.g., *flame ionization detector*) or those forming part of the “sampling train” (e.g., *NIOSH-approved pump*).

**Sample Type Personal**–The significant rise in IAA between rounds 1 and 2 may be explained by the modification of the definition of this category between these two rounds. The original *Sample Type* category encompassed phrases providing evidence of *both* personal *and* stationary sampling. However, the latter were found to be sparser and to often take the form of long and complex phrases that specify the location of the stationary sampling equipment (e.g., *Air was sampled at a* *position where portions of newly produced asphalt were emptied repeatedly*). As well as being difficult to spot, such phrases could also be confused with *Industry/Workplace* annotations, since these can also correspond to locations. Reducing the scope of this category to phrases denoting personal sampling only resulted in considerable improvements in IAA from round 2 onwards. This is because such phrases tend to be shorter, more explicit and far less variable (e.g., *personal sampling* or *personal exposures*). As explained above, recognising evidence of stationary sampling is not strictly necessary if evidence of personal sampling can be detected accurately, since an absence of personal sampling mentions is likely to indicate that stationary sampling was undertaken.

**Job Task/Activity**–The large amount of annotation evidence collected in round 2 revealed the complex and diverse nature of phrases belonging to this category. Substantial extensions to the guidelines based on this evidence resulted in increases in both exact and, more significantly, relaxed IAA, in round 3. Updates included clarifying that activities can be described using either nouns *or* verbs, and that annotations should always correspond to specific, well-defined activities. While single nouns/verbs may sometimes fulfil the latter criterion (e.g., *drilling*, *welding*), more vague nouns/verbs should only be annotated if the neighbouring context clarifies the nature of the activity and can be included in the annotated span (e.g., *concrete* *pouring*). Additionally, given that activities can be described in many ways and with varying levels of detail, a range of syntactic and semantic criteria/restrictions were introduced to try to reduce uncertainly among annotators regarding the exact types of words/phrases and details that should be included within annotated spans. For example, in addition to the basic activity word (***bold*** in the following examples), annotated spans should include additional words *(**underlined)* if they occur in specific contexts within the immediate vicinity of the activity word, and as long as they provide specific types of information about the activity. Specific contexts include words within the same phrase as activity-denoting nouns (e.g., *rock* ***drilling***); objects of activity-denoting verbs (e.g., ***laying*** *conduit*); and prepositional phrases that follow activity-denoting words (***installation*** *of drop ceilings*). These words/phrases must provide information about the item affected by the activity (e.g., ***scraping*** *poultry houses*); the initial state of the activity (e.g., ***lift*** *the concrete block* *out of the pavement*); or the resultant state of the activity (e.g., ***drill*** *holes* *through the block*).

**Industry/Workplace**–Similarly to *Job Task/Activity*, the wide semantic scope of this category, particularly in terms of the huge variety of workplaces that are described in articles, only became fully apparent following the large-scale annotation effort in round 2. The guidelines were thus extended prior to round 3 to enumerate and exemplify a range of phrase types that provide information about where people work. These include indoor work areas (*diesel use mines*, *tollbooths*), outdoor work areas (*four-lane motorway*, *heavy repair area*), vehicles driven by workers (*diesel fork-lift truck*, *lead locomotive*), large pieces of heavy equipment (*asphalt mill*, *pneumatic drills*) and specific features of the working environment (*surface*, *underground*, *enclosed workspaces*). While these additions resulted in increased agreement in round 3, they had a smaller impact than the changes made to the *Job Task/Activity* guidelines. Given the level of variability in the descriptions of working environments encountered in articles, it is impossible to enumerate them all in the guidelines. As such, determining which types of phrases are suitable and sufficiently specific to be annotated using this category can be challenging, and may be at least partly reliant on the annotator’s depth of knowledge about a particular industry.

Although round 4 demonstrated an increasing convergence of relaxed IAA rates across different categories, discrepancies between exact and relaxed IAA remained to varying degrees. Span disagreements can occur particularly when NEs are described using unexpected or complex phrase structures. In terms of *Substance or Exposure Measured* spans, for example, information about state/form most typically occurs within the same noun phrase as the name of the substance (e.g., *respirable* *crystalline silica*). However, despite relevant exemplification in the guidelines, state/form information was sometimes overlooked when it occurred *after* the noun phrase introducing the substance, (e.g., *particulate matter* *less than or equal to 10 μm diameter*). For *Job Task/Activity*, the complex nature of certain task descriptions, which can sometimes extend to multiple phrases that follow the activity-denoting word, can also lead to span discrepancies, e.g., *milling of asphalt* *from concrete highway pavement*.

### Variability of inter-annotator agreement

**Fig 2:**
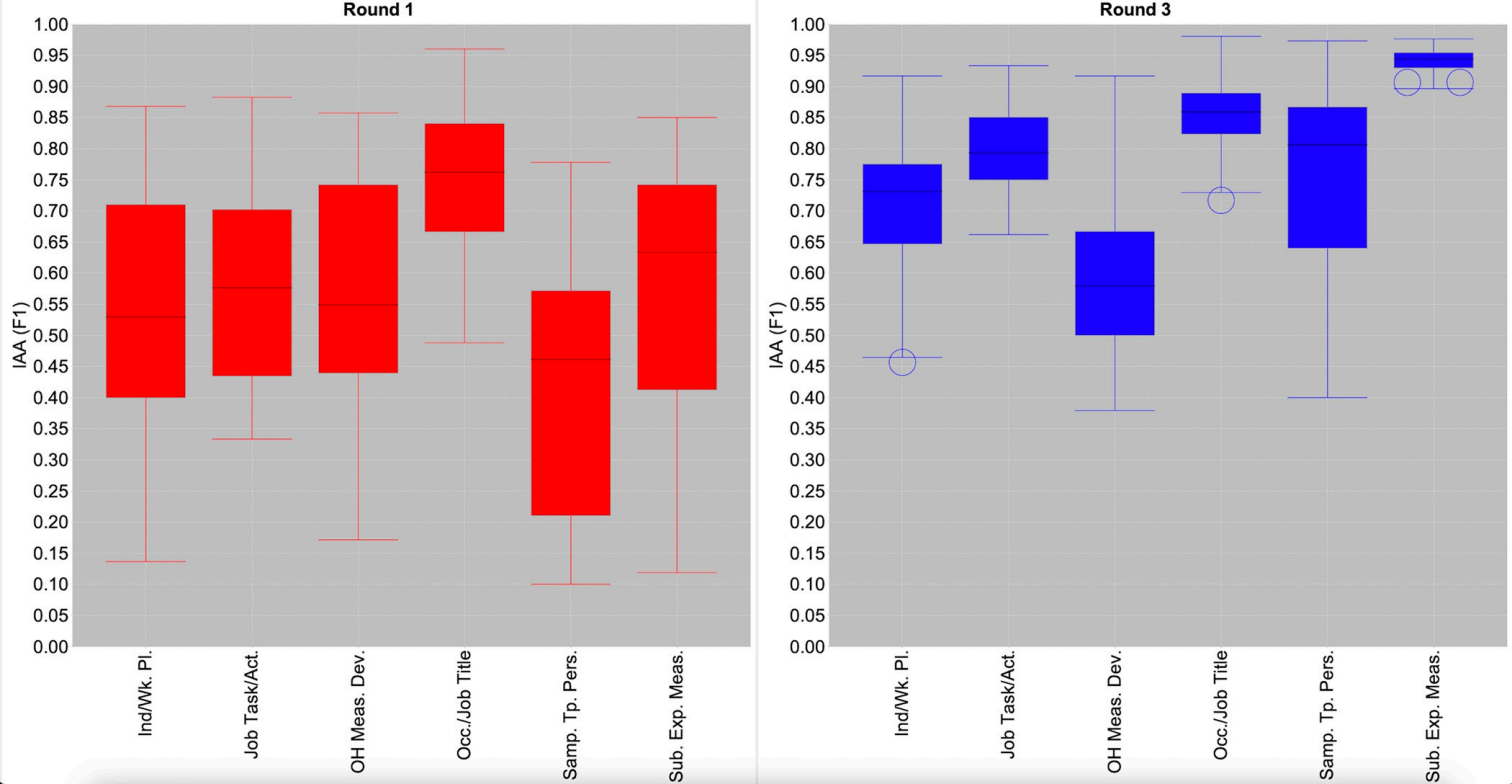
Comparison between pairwise relaxed IAA distributions for rounds 1 and 3. The boxes illustrate the interquartile range (IQR) for each respective category and round, i.e., the range of the middle 50% of pairwise IAA rates. The line within each box represents the *median* (middle) pairwise IAA rate among all annotators for the respective category and round. The bottom of each box represents the *lower quartile*; 25% of pairwise IAA rates fall below this value. The top of each box represents the *upper quartile;* 25% of pairwise IAA rates fall above this value**. The “whiskers” outside of the boxes extend as far as the minimum and maximum pairwise IAA values, as long as these fall with 1.5 times the IQR.** Any IAA rates that fall outside of this range are considered as outliers, and are marked with circles. “Ind/Wk. Pl” = Industry/Workplace; “Job Task/Act.” = Job Task/Activity; “OH Meas. Dev.” = OH Measurement Device; “Occ./Job Title” = Occupation/Job Title; “Samp. Tp. Pers” = Sample Type Personal; “Sub. Exp. Meas” = Substance or Exposure Measured.

While the increases in the average IAA rates depicted in Fig 1 provide evidence of the *overall* success of our iterative corpus development process, we also wanted to assess the degree to which this positive impact extended across *all* annotators. Fig 2 provides box and whisker plots that illustrate the *range* of category-wise IAA rates obtained for each possible pairing of the different annotators in rounds 1 (diesel exhaust) and 3 (RCS). Both rounds involved the annotation of a common set of articles by all annotators, thus allowing meaningful comparisons to be made. For ease of comparison between the two rounds, we show only the relaxed matching rates; the general trends for exact matching are similar.

A notable trend in Fig 2 is that lower bounds of IAA increased for *all* categories between rounds 1 and 3, and significantly so for most categories. This indicates that all annotators developed an improved understanding of the guidelines for all categories between these two rounds. For most categories, there was also a considerable increase in the median IAA, such that in round 3, an IAA of 0.70 F1 or above was achieved by half of the annotator pairs for all categories apart from *OH Measurement Device*. Moreover, the upper bounds of relaxed IAA increased for all categories between rounds 1 and 3, and reached above 0.90 F1 for at least some pairs of annotators for *all* NE categories in round 3. This provides strong evidence that the refined guidelines are sufficiently comprehensive and clear to allow consistent annotations to be produced independently by different annotators. Furthermore, the reduced plot lengths for all categories in round 3 compared to round 1 demonstrate reduced variability between the IAA scores achieved among different annotator pairs, and thus an increasingly shared understanding of the task. The most significant reductions are for *Job Task/Activity*, *Occupation/Job Title* and *Substance or Exposure Measured*.

Despite these positive trends, a considerable degree of variability in pairwise IAA remained in round 3 for *OH Measurement Device*, *Industry/Workplace* and *Sample Type Personal*. For *OH Measurement Device* and *Industry/Workplace*, this is likely to stem from differing levels of specialised knowledge among annotators regarding occupational hygiene equipment and/or familiarity with specific industries, as discussed above. Nevertheless, the skewed nature of the round 3 plots for both *Industry/Workplace* and *Sample Type Personal* demonstrates smaller variability at the upper end of the scale for these categories. This indicates that there is a stronger group of annotators who have a good level of understanding about how to mark-up mentions of them.

**Fig 3:**
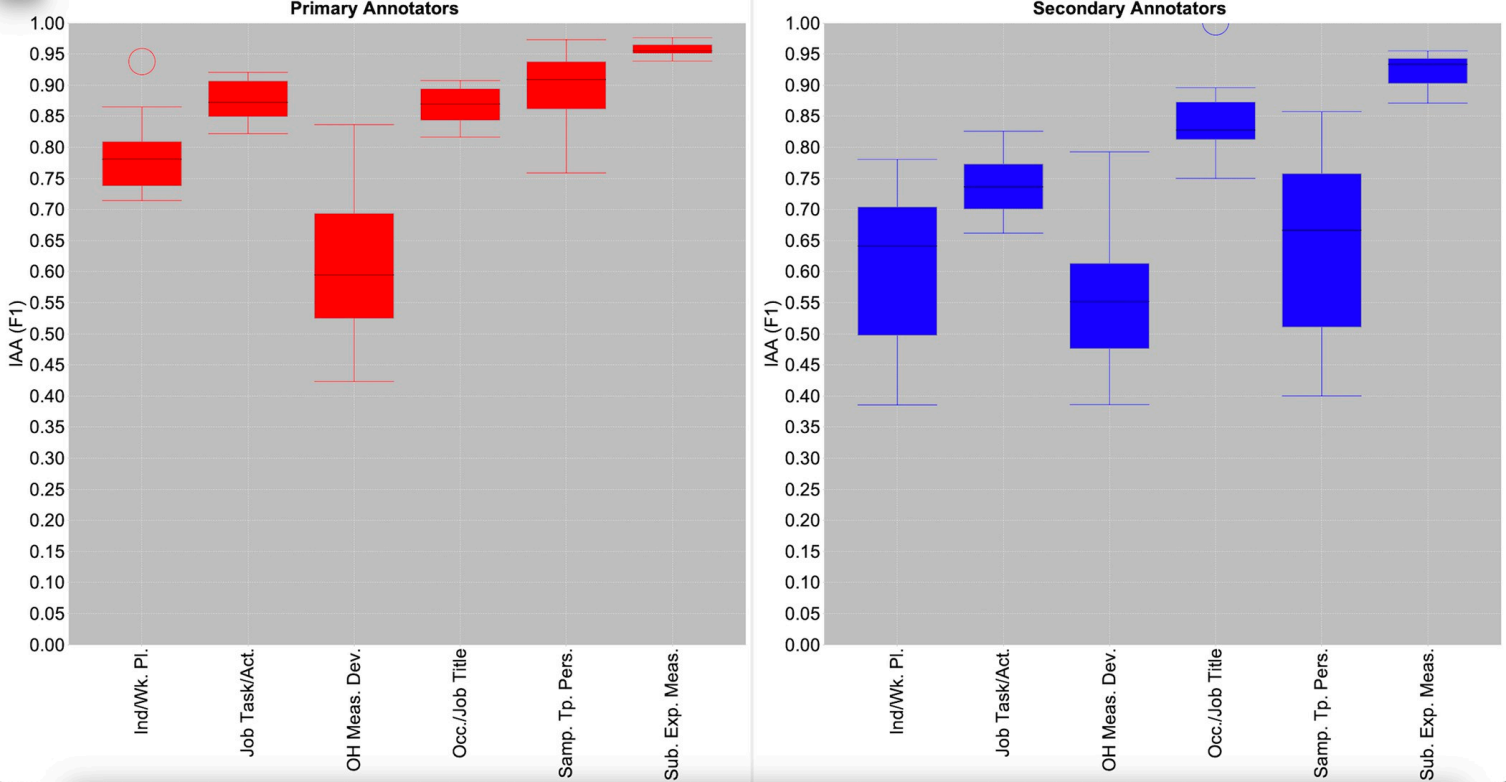
Comparison between pairwise relaxed IAA rates for primary and secondary annotator groups in round 3. “Ind/Wk. Pl” = Industry/Workplace; “Job Task/Act.” = Job Task/Activity; “OH Meas. Dev.” = OH Measurement Device; “Occ./Job Title” = Occupation/Job Title; “Samp. Tp. Pers” = Sample Type Personal; “Sub. Exp. Meas” = Substance or Exposure Measured.

The latter observation is reinforced in Fig 3, whose box and whisker plots depict the differences between the ranges of pairwise relaxed IAA rates obtained in round 3 when the primary and secondary groups of annotators are considered separately. Except for *OH Measurement Device*, the degree of variability among IAA rates in the primary group is always smaller than for the secondary group, and significantly so for *Industry/Workplace* and *Sample Type Personal*. The upper and lower bounds of pairwise IAA are both consistently higher for the primary group, while pairwise IAA among all primary annotators consistently rises above 0.70 F1 (and above 0.80 F1 for *Occupation/Job Title*, *Substance or Exposure Measured* and *Job Task/Activity*). These observations confirm that annotators in the primary group have a high level of shared understanding about how to annotate most categories. This strengthens our assumption that these annotators would produce a good quality set of annotations in round 4, to provide a strong basis for the annotations in the final set.

Although Fig 3 shows that the primary annotators generally produced more consistent annotations than the secondary group, it illustrates that high pairwise IAA are also achievable among members of the secondary group. Indeed, there is very little difference between the plots of the primary and secondary groups for *Occupation/Job Title* and *Substance or Exposure Measured*. This indicates that mentions of both categories can be annotated to a high standard by all annotators. For the other NE categories, at least some pairs of secondary annotators achieved IAA rates that fall within the same range as the primary annotators. This provides evidence that some of the secondary annotators’ annotations are of a sufficient quality to augment those produced by the primary annotators. Nevertheless, the greater degree of variation in annotation behaviour among secondary annotators motivates our approach of developing customised rules to combine the annotations from each pair of annotators to obtain an optimal consolidated final set.

The pattern for *OH Measurement Device* in Fig 3 is distinct from that of other categories. Although the upper and lower bounds of pairwise IAA are both marginally higher for the primary group, the degree of variability remains almost equally large for both the primary and secondary groups. For both groups, IAA can reach above 0.78 F1, and fall below 0.45 F1. This provides further evidence that the precise knowledge and experience required to annotate this category accurately appears to differ from that of other categories.

### Final corpus analysis

In this section, we present statistics and discuss characteristics and trends of the annotations in the final version of the complete corpus, i.e., the 101 papers concerning either diesel exhaust or RCS exposures, following the application of the rule-based algorithm to combine annotations.

For each NE category, Table 3 provides three different statistics, as follows:

- **Total annotations**—Total number of spans annotated in the corresponding category.
- **Unique spans–** Number of distinct spans annotated in the corresponding category, after converting to lower case.
- **Unique span frequency–**Average number of times that each unique span in the corresponding category was annotated.

**Table 3:** Final corpus statistics.

Category	Total Annotations	Unique Spans	Unique Span Frequency
Industry/Workplace	2887	964	2.99
Job Task/Activity	1720	1072	1.60
OH Measurement Device	932	431	2.16
Occupation/Job Title	2219	680	3.26
Sample Type Personal	531	116	4.58
Substance or Exposure Measured	7909	825	9.59

*Substance or Exposure Measured* is by far the most annotated category, with almost three times as many annotations as the second most annotated category, i.e., *Industry/Workplace*. Measurement campaigns described in scientific articles often refer to several different substances, each of which may be mentioned multiple times in text. For example, a given substance may be mentioned in the context of each of the different circumstances under which its exposure levels were measured. These circumstances may include varying combinations of occupations, workplaces and/or job tasks. Such patterns of mentions help to explain why the average frequency of each unique *Substance or Exposure Measured* span is higher than any other NE category. Indeed, several substances that are highly pertinent to diesel exhaust and RCS exposures are mentioned several hundred times in the corpus, e.g., *organic carbon*, *elemental carbon*, *dust*, *quartz*, *silica* and their abbreviations. Although our focus is on diesel exhaust and RCS exposures, mentions of other exposed substances discussed in the articles were also annotated, given our aim to develop NER tools that are sufficiently general to allow application to articles concerning other types of exposures. While this partly explains the high number of unique spans annotated in this category (i.e., 825), many span variations also occur because of the inclusion of information about state or form. This information can be specified in various ways and at different levels of detail. For example, there are 46 unique spans that mention the word *diesel*, including *diesel emissions*, *diesel particles*, *diesel aerosol particulates*, *submicrometer-sized diesel particles* and *respirable fraction of diesel exhaust particulate*, among others.

Although the total number of *Job Task/Activity* annotations is around 5 times smaller than the total number of *Substance or Exposure Measured* annotations, the number of unique *Job Task/Activity* spans annotated is 20% larger. Of the 1072 unique *Job Task/Activity* spans annotated, only 17 occur ten or more times in the corpus, mostly corresponding to single word annotations like *drilling*, *demolition* or *paving*. In general, descriptions of job tasks and activities are highly variable and largely unique. This is partly due to the wide range of different activities associated with each occupation, whose specific details are likely to vary according to the context in which they are described. Each activity and its associated details can be described using a variety of different phrase types and structures, including noun or verb phrases, verb objects, prepositional phrases, passive constructions, etc. As an example, descriptions of activities involving the word *loading* include information about the target receptacle *(loading the trucks; loading furnaces);* information about the item being loaded (*rock is loaded; loading ore; loading of debris; load cargo*); or both of these (*loading of crushed rock into dump trucks; loading broken rock onto the conveyor belt*).

Several very general workplaces are frequently annotated as *Industry/Workplace*. These include *mine/mines*, which collectively account for 229 annotations (i.e., 8% of the total annotations in this category). While this helps to explain why the average unique span frequency is almost twice as high as *Job Task/Activity*, the number of unique annotated spans is also very high (i.e., 964). This is because a range of similar workplaces can be described in a variety of ways and at different levels of granularity. For example, the word *mine* forms part of a further 66 unique annotated spans. Variations in the types of additional information provided about the workplace include the type of material mined (e.g., *oil shale mines; coal mines*, *potash mines*), the characteristics of the mine (*underground mines; open pit mines; surface mines*), a combination of the above (*underground iron ore mine*) and specific areas of mines (*production areas of the mines; intake shafts of the mines; underground mine roadway*).

Although *Occupation/Job Title* ranks third in terms of the total number of annotated spans, it ranks fourth in terms of the number of unique spans, with over 30% fewer unique spans than *Industry/Workplace* or *Job Task/Activity*. This is because the number of unique occupations is much smaller than the range of potential places where such jobs may be carried out and the types of tasks that they involve. The most frequently annotated *Occupation/Job Title* spans are short, general job titles, e.g., *miners*, *drivers*, *mechanics* and *carpenters*. Nevertheless, the 680 unique spans annotated exhibit considerable diversity, according to the frequent mention of more specific job roles. As an example, there are 62 different unique spans that include the word *driver*, which may specify the nature of the work (*pickup & delivery driver; long-haul driver*) or the types of vehicles driven (*train driver; bus driver; truck driver*). The latter may be specified in various ways and at differing levels of detail *(drivers of trains; drivers of short-haul suburban goods trains; short-haul locomotive driver*).

The total number of *OH Measurement Device* annotations is significantly lower than the four NE categories discussed above, and each unique span is mentioned relatively infrequently (on average 2.16 times, which is lower than all other categories apart from *Job Task/Activity*). However, the number of unique spans is still reasonably high (i.e., 431). While there is a reasonably limited number of *types* of devices/equipment, there are many possible variations in their specific descriptions. For example, there are 82 unique spans in which the word *cyclone* appears, ranging from a single word to spans that include material of manufacture (*nylon cyclone; aluminium cyclone*), size (*10-mm cyclone*), manufacturer name (*Dewell-Higgins cyclone*), state/form targeted (*cyclone-type respirable dust samplers; PM1*.*0 cyclone*) and combinations of the above (*10 mm nylon cyclone; SKC Ltd conductive plastic cyclone; MSA 10 mm nylon Dorr-Oliver personal cyclone)*. Annotations in this category can furthermore correspond to device names or model numbers (e.g., *P-Trak*, *Sidepak AM510*, *SCC1*.*062 Triplex*), which further helps to account for the wide diversity of spans.

*Sample Type Personal* is the least annotated category, with a total of 531 annotations in the whole corpus. However, on average, each unique annotated span occurs more frequently than all other categories apart from *Substance or Exposure Measured*. This is because evidence of personal sampling most frequently takes the form of a small number of very simple phrases. For example, the phrases *personal sample(s)*, *breathing zone(s)*, *personal exposure(s)*, *personal sampling* and *personal measurement(s)* collectively for around 60% of the total number of annotations in this category.

### NER experiments

#### Dataset characteristics

Table 4 reports average annotation statistics for the ten splits of the data used in the cross-validation experiments. The final column shows the average percentages of annotations in the test folds whose spans do not occur at all in the training data. This is intended to provide a general indication of the likely difficulty for the fine-tuned models to predict the annotations belonging to NE each category. For example, if the test fold includes a high percentage of annotations for a particular category that were seen by the model during training, then it is assumed that the category will be easier for the model to predict, as it will already be conditioned to detect many of its mentions.

**Table 4:** Average annotation statistics in the data splits used for cross-validation.

Category	Average number of annotations in training folds	Average number of annotations in test fold	Average percentage of test fold annotations not present in training folds (standard deviation in parentheses)
Industry/Workplace	2144	238	23% (± 4%)
Job Task/Activity	1335	148	49% (± 6%)
OH Measurement Device	674	75	39% (± 4%)
Occupation/Job Title	1760	196	20% (± 3%)
Sample Type Personal	438	49	16% (± 5%)
Substance or Exposure Measured	6307	701	6% (± 1%)

Across the different test folds, *Substance or Exposure Measured* consistently exhibits the lowest and least variable percentage of previously unseen annotations. This can be explained partly by the large number of training instances in this category, combined with its relatively narrow semantic scope. Conversely, the highly diverse nature of *Job Task/Activity* annotations results in this category having the highest and most variable proportion of previously unseen annotations.

Table 5 provides statistics regarding the annotations in the held-out test data set. While the proportions of previously unseen annotations for some categories vary only slightly from the averages reported for the cross-validation test folds in Table 4, the percentages of *Job Task/Activity* and *Occupation/ Job Title* are significantly higher, and are thus likely to pose greater challenges for the models.

**Table 5:** Annotation statistics in the held-out test set.

Category	Annotation Count	Percentage of annotations not in training and/or development sets
Industry/Workplace	328	28%
Job Task/Activity	84	68%
OH Measurement Device	121	37%
Occupation/Job Title	177	42%
Sample Type Personal	84	11%
Substance or Exposure Measured	690	8%

#### Cross-validation results

Table 6 reports the mean results obtained by the two BERT-based models across the ten different splits of the data in the cross-validation experiments. The results demonstrate that both the token-based and span-based models exhibit similarly encouraging levels of performance. In terms of F1 scores, there are only minor differences between the two models, and the scores compare favourably to those achieved for BERT-based NER models that have been applied to scientific articles in other specialised domains (e.g., [86,95]).

**Table 6:** Mean cross-validation results for the two BERT-based models over ten folds.

Category	Exact matching						Relaxed matching					
	Span-based model			Token-based model			Span-based model			Token-based model		
	P	R	F1	P	R	F1	P	R	F1	P	R	F1
Substance or Exposure Measured	***0*.*91*** ±0.01	***0*.*93*** ±0.01	***0*.*92*** ±0.01	0.90 ±0.01	***0*.*93*** ±0.01	0.91 ±0.01	**0.93** ±0.01	0.95 ±0.01	**0.94** ±0.01	0.92 ±0.01	**0.96** ±0.01	**0.94** ±0.01
Sample Type Personal	***0*.*81*** ±0.07	***0*.*86*** ±0.07	***0*.*83*** ±0.05	0.80 ±0.06	0.85 ±0.04	0.82 ±0.05	**0.87** ±0.07	0.91 ±0.06	0.89 ±0.05	**0.87** ±0.04	**0.93** ±0.04	**0.90** ±0.03
Industry/Workplace	***0*.*77*** ±0.03	0.79 ±0.03	***0*.*78*** ±0.02	0.75 ±0.02	***0*.*80*** ±0.02	***0*.*78*** ±0.02	**0.83** ±0.02	0.83 ±0.02	0.83 ±0.01	0.82 ±0.03	**0.86** ±0.02	**0.84** ±0.01
Occupation/Job Title	***0*.*92*** ±0.02	0.92 ±0.02	***0*.*92*** ±0.02	0.91 ±0.02	***0*.*93*** ±0.02	***0*.*92*** ±0.02	**0.94** ±0.02	0.93 ±0.02	0.93 ±0.01	**0.94** ±0.02	**0.94** ±0.03	**0.94** ±0.01
OH Measurement Device	***0*.*77*** ±0.08	0.77 ±0.05	***0*.*77*** ±0.06	0.74 ±0.06	***0*.*78*** ±0.06	0.76 ±0.05	**0.84** ±0.06	0.83 ±0.05	0.83 ±0.04	0.82 ±0.05	**0.86** ±0.05	**0.84** ±0.03
Job Task/Activity	***0*.*78*** ±0.05	***0*.*74*** ±0.05	***0*.*76*** ±0.05	0.73 ±0.05	***0*.*74*** ±0.04	0.73 ±0.04	**0.84** ±0.05	0.79 ±0.05	0.82 ±0.04	0.83 ±0.05	**0.82** ±0.05	**0.83** ±0.03
Overall	***0*.*86*** ±0.01	***0*.*88*** ±0.01	***0*.*87*** ±0.01	0.85 ±0.01	***0*.*88*** ±0.01	0.86 ±0.00	**0.90** ±0.01	0.90 ±0.01	0.90 ±0.01	0.89 ±0.01	**0.92** ±0.01	**0.91** ±0.00

P = Precision; R = Recall. The highest scores achieved for exact matching are shown ***bold italics***, while the highest scores for relaxed matching are shown using **bold underline.** If both models achieve equal scores for a particular combination of metric and matching criteria, then both scores are highlighted accordingly. The figures preceded by ± denote the degree of variability among the results for different folds, in terms of standard deviation.

The average *Overall* scores for the cross-validation experiments are high, reaching 0.85 or above for both relaxed and exact matching, in terms of precision, recall and F1. The low standard deviation statistics indicate minimal variation in these overall scores across the different folds, which helps to demonstrate the general robustness of the models. For individual NE categories, the average scores for all three metrics fall above 0.73 for exact matching and 0.79 for relaxed matching. The high performance for *Substance or Exposure Measured* is expected, given the large number of training instances, most of which also occur in the test folds. However, the results for other categories indicate that the models have successfully learnt to generalise and correctly predict previously unseen annotations using significantly fewer training annotations. This is most notable for *Occupation/Job Title*, whose performance and cross-fold stability are on par with *Substance or Exposure Measured*. However, very respectable results are also achieved for *Job Task/Activity*, especially given the high percentage of previously unseen annotations in the test folds and the potential complexity of annotations in this category. While *Sample Type Personal* and *OH Measurement Device* achieve good average results with even smaller numbers of training annotations, their larger degree of performance variability across different folds indicates that further training data may be beneficial to ensure more stable performance.

While the two models are comparable in terms of their F1 scores, they exhibit different, but complementary, behaviours in other respects. For example, the span-based model tends to achieve the highest precision, and is slightly more successful in predicting correct span lengths. This latter observation provides evidence that the richer representations used by this model allow it to learn the characteristics of valid entity spans more accurately. Meanwhile, the token-based model generally obtains higher recall, and performs a little more robustly across different folds, as indicated by the slightly lower standard deviation figures. The observed precision and recall patterns are in line with the trends reported in the comparison of span-based and token-based NER in [90], while the generally complementary features of the two types of models have been noted in other previous studies [96,97], in which they were combined to further boost performance.

#### Held-out test set results

Table 7 reports the results of applying the two models to the held-out test set. The general behavioural trends of the two models are very similar to those reported above for the cross-validation experiments, which helps to demonstrate that they exhibit stable and predictable behaviour. While performance statistics are generally slightly lower than for the cross-validation experiments, this is to be expected, given the more challenging characteristics of the held-out test set, i.e., most categories have higher proportions of previously unseen annotations. Nevertheless, precision, recall and F1 scores of 0.70 or above are obtained by one or both models, using both relaxed and exact matching criteria, for all categories apart from *Job Task/Activity*. This category was expected to be particularly challenging, according to its previously noted complex characteristics, combined with the particularly high proportion of previously unseen annotations in the held-out test set. It is also noteworthy that *Occupation/Job Title* achieves the best F1 scores among all NE categories for both exact matching (span-based model, 0.87) and relaxed matching (token-based model, 0.94), despite the high proportion of previously unseen annotations in the held-out test set (42%).

**Table 7:** Performance of the two fine-tuned BERT models on the test set.

Category	Exact matching						Relaxed matching					
	Span-based model			Token-based model			Span-based model			Token-based model		
	P	R	F1	P	R	F1	P	R	F1	P	R	F1
Substance or Exposure Measured	***0*.*85***	0.88	***0*.*86***	0.83	***0*.*90***	***0*.*86***	**0.88**	0.91	**0.90**	0.87	**0.94**	**0.90**
Sample Type Personal	***0*.*83***	***0*.*87***	***0*.*85***	0.82	0.82	0.82	**0.88**	**0.91**	**0.89**	**0.88**	0.88	0.88
Industry/Workplace	***0*.*76***	0.70	0.73	0.73	***0*.*74***	***0*.*74***	**0.80**	0.75	0.77	**0.80**	**0.81**	**0.80**
Occupation/Job Title	***0*.*90***	***0*.*84***	***0*.*87***	0.85	***0*.*84***	0.85	0.93	0.88	0.90	**0.95**	**0.93**	**0.94**
OH Measurement Device	***0*.*74***	***0*.*74***	***0*.*74***	0.63	0.73	0.67	**0.78**	0.78	**0.78**	0.74	**0.83**	**0.78**
Job Task/Activity	***0*.*59***	0.67	***0*.*63***	0.58	***0*.*68***	***0*.*63***	**0.66**	0.70	0.68	0.65	**0.75**	**0.70**
OVERALL	***0*.*81***	0.81	***0*.*81***	0.78	***0*.*83***	0.80	**0.85**	0.85	0.85	0.84	**0.88**	**0.86**

P = Precision; R = Recall. The highest scores achieved for exact matching are shown ***bold italics***, while the highest scores for relaxed matching are shown using **bold underline.** If both models achieve equal scores for a particular combination of metric and matching criteria, then both scores are highlighted accordingly.

Table 8 provides some examples of NEs that were correctly predicted by one or both of the models when applied to the held-out test set, and whose spans do not occur in the training or development sets. The table confirms that both models can correctly detect previously unseen NEs that include various types of details, and which cover much of the syntactic and semantic diversity of the different categories that was outlined in the *Final Corpus Analysis* section above.

**Table 8:** Examples of correct model-predicted annotations.

Category	Correctly predicted NEs
**Industry/Workplace**	• *clerk’s office* • *citrus harvest site* • *ambulance* • *forklifts* • *unenclosed work sites* • *agricultural operations*
**Job Task/Activity**	• *diesel engine maintenance* • *excavation of a large disposal pit* • *scraping poultry houses* • *expansion joints were being sawed in the fresh concrete* • saw through the decking of an existing bridge • **old pavement could be removed**
**OH Measurement Device**	• *IOM respirable dust samplers* • *3-piece 37 mm cassette* • *photoelectric aerosol sensor* • *Sierra 290 series personal cascade impactor* • open face polystyrene cassette of 37 mm diameter • 7 hole sampler • **conductive plastic sampler with a respirable dust cyclone**
**Occupation/Job Title**	• *blacksmiths* • *stone-processing plant operators* • *parking ramp attendants* • *grape harvest workers* • *drivers of shunting locomotives* • *refinery workers* • *short-haul locomotive driver* • plumber • **ironworkers**
**Sample Type Personal**	• *personal respirable-dust samples* • *operator’s breathing zone* • **personal EC samples**
**Substance or Exposure Measured**	• *acetaldehyde* • *levoglucosan* • *diesel particulate* • *inhalable endotoxin* • *respirable coal dust* • *submicron mineral dust* • *total foliar dust* • diesel exhaust fume • **airborne culturable bacteria**

*Italics* = predicted by both models; underlined = predicted by token-based model only; **bold** = predicted by span-based model only.

An analysis of NEs that were incorrectly predicted by the models reveals that many of them relate to more subtle aspects of the annotation guidelines, which may be difficult to learn using annotated evidence alone. Examples include the following:

- Determining which activity-denoting words are too vague to stand alone as *Job Task/Activity* annotations. Given that the training data includes examples such as *welding* and *welding of structural steel*, it is difficult for the models to determine that *loading* is considered too vague to occur as a single word annotation, especially since it occurs in very similar contexts to *welding* (e.g., *loading of debris*).
- Distinguishing references to (groups of) workers who do not constitute the *subjects* of exposure studies (e.g., *industrial hygienists*), which are out of scope for annotation, according to the guidelines for *Occupation/Job Title*. Such distinctions can be difficult for the models to make, since there is limited negative evidence in the training corpus, and they look and behave like many valid *Occupation/Job Title* annotations.
- Distinguishing between similar phrases that describe occupational hygiene devices, some of which are out of scope according to the guidelines for *OH Measurement Device*. For example, both *cyclone-type respirable dust samplers* and *open face total dust sampler* constitute valid annotations, because they provide details about the characteristics of the sampler type. However, *personal diesel exhaust aerosol samplers* is out of scope, because it mentions only the substance that the device is used to collect, rather than any specific details regarding the sampler type characteristics.

For all NE categories, there is a certain degree of discrepancy between the exact and relaxed evaluation scores. An examination of the incorrectly predicted spans reveals a mixture of cases in which the model-predicted spans appear more correct than the expert-annotated spans, and vice-versa. Examples of cases where the models predict longer spans that seem more correct than the expert-annotated spans include *rural* *terminals* vs. *terminals* (Industry/Workplace), *driving* *in traffic* vs. *driving* (Job Task/Activity), *small* *impactor* vs. *impactor* (OH Measurement Device), *equipment* *operators* vs. *operators* (Occupation/Job Title), *personal* *air monitoring* vs. *personal* (Sample Type Personal) and *diesel exhaust* *particulate* vs. *diesel exhaust* (Substance or Exposure Measured). Such examples provide evidence that the models have correctly grasped that annotations should typically correspond to *complete* phrases. The models tend to predict spans that are shorter and simpler than the expert-annotated spans in cases where the latter correspond to rare NE structures. Examples include *remove old asphalt* vs. *remove old asphalt* *from an interstate highway* (Job Task/Activity) and *outside* vs. *outside* *the furnace hall* (Industry/Workplace). For the second example, however, the span-based model correctly predicted the longer span.

## Conclusions

As a first step towards developing NLP tools for the occupational exposure assessment domain, we have constructed a novel NE-annotated corpus of scientific articles concerning occupational exposures to diesel exhaust and RCS, in which high levels of IAA are attainable for all NE categories. To demonstrate the value of the corpus, we have used it as the basis to fine-tune two state-of-the-art PLM-based NER models to automatically recognise mentions of our target NE categories. Both models achieved results that mostly exceed 0.80, in terms of precision, recall and F1 score, which are comparable with PLM-based NER models applied to scientific articles in other specialised domains, and are able to recognise NEs with diverse characteristics.

The exploration of large language models (LLMs), such as Generative Pre-Trained Transformer (GPT) models [98], represents a promising future direction to further improve upon NER results. LLMs are pre-trained with larger amounts of data than BERT, and are able to perform a range of tasks (including NER [99]), either without any fine-tuning, or else by using only a small number of task-specific examples [100]. Furthermore, several recent studies have shown that that LLMs can be fine-tuned to follow annotation guidelines [101,102], which may provide scope to better handle the more complex/subtle aspects of our annotation scheme that were problematic for PLM-based models.

Additional future work will involve enriching the corpus with further levels of annotation, including linking entities to concepts in domain specific databases and identifying relations among entities, to support the development of a range of more sophisticated NLP models. It is hoped that the future integration of such models into an efficient semantic search system for occupational exposure literature will considerably increase the feasibility of developing and maintaining a repository of JEMs with fine-grained exposure estimates. This in turn will help to increase the accuracy of differentiating individuals’ lifetime exposures and levels, which could ultimately support the development of improved preventative workplace policies to reduce the incidence of non-communicable diseases.

## Supporting information

S1 Appendix Named entity annotation guidelines. (PDF)

S2 Appendix Rule-based combination of annotations. (PDF)

S3 Appendix Detailed annotation analysis. (PDF)
