# Supplementary material for: Supporting the working life exposome: Annotating occupational exposure for enhanced literature search
Source: PLoS One. 2024 Aug 15;19(8):e0307844. doi: 10.1371/journal.pone.0307844 (PMC11326626; doi:10.1371/journal.pone.0307844)
Supplement: S1 Appendix — (PDF) [file pone.0307844.s001.pdf]

S1 Appendix: Named entity annotation guidelines

Paul Thompson[1], Ioannis Basinas[2] and Sophia Ananiadou[1]

[1] National Centre for Text Mining, Department of Computer Science, University of Manchester, UK

[2] Centre for Occupational and Environmental Health, School of Health Sciences, University of Manchester, UK

IF YOU USE THESE GUIDELINES, PLEASE ATTRIBUTE THE AUTHORS AND AFFLIATIONS ABOVE, AND CITE THE FOLLWING ARTICLE:

Paul Thompson, Sophia Ananiadou, Ioannis Basinas, Bendik C. Brinchmann, Christine Cramer, Karen S. Galea, Calvin Ge, Panagiotis Georgiadis, Jorunn Kirkeleit, Eelco Kuijpers, Nhung Nguyen, Roberto Nuñez, Vivi Schlünssen, Zara Ann Stokholm, Evana Amir Taher, Håkan Tinnerberg, Martie Van Tongeren and Qianqian Xie (2024). Supporting the working life exposome: annotating occupational exposure for enhanced literature search. PLOS ONE.

Table of Contents

1. Introduction 1

2. General Guidelines 2

2.1 Annotation Scope 2
2.2 Annotation Span 4
2.3 Discontinuous Spans 5

3. Category-specific guidelines 8

3.1 Industry / Workplace 8
3.2 Occupation / Job Title 15
3.3 Job Task / Activity 22
3.4 Substance or Exposure Measured 31
3.5 Occupational Hygiene (OH) Measurement Device 36
3.6 Sample Type: Personal 43

4. Quick reference guides 46

4.1 Overview 46
4.2 General Guidelines 48
4.3 Industry / Workplace 49
4.4 Occupation / Job Title 50
4.5 Job Task / Activity 51
4.6 Substance or Exposure Measured 52
4.7 Occupational Hygiene (OH) Measurement Device 53
4.8 Sample Type: Personal 54

1. Introduction

The Exposome Project for Health and Occupational Research (EPHOR) aims to improve our ability to study the working life exposure by developing a toolbox of methods for the collection, storage, analysis and interpretation of working-life data, and by increasing our understanding about the way in which the interaction of co-exposures can result in disease onset. Part of this process is the development of the EuroJEM, an enhanced and harmonised Job Exposure Matrix (JEM) for European populations, which combines existing smaller JEMs with dynamic methods for updating its coverage, both in terms of adding new agents (i.e., those corresponding to emerging exposures) and revising exposure estimates to account for the constantly changing nature of exposure in workplaces.

The use of text mining (TM) is explored as a means to assist with the dynamic update of the EuroJEM. It is anticipated that TM will assist us in identifying relevant literature more accurately and efficiently, by helping to locate articles that mention combinations of parameters of interest.

Annotations form an integral component of the TM process. This annotation task focuses on marking up *entities* in scientific literature articles; each entity corresponds to a mention of concept of interest within an article. The entity annotation task entails identifying specific types of entities and then labelling each one with an appropriate category label.

These guidelines use the following terminology to help to define the task:

Scope refers to the range of words/phrases that should be considered for annotation.

Span refers to the exact extent of text that should be marked up to create an annotation

Each section in these guidelines enumerates which types text spans should be *included* or *excluded* for each annotation category, in terms of annotation scope and/or span, supported by examples. Each example constitutes (part of) a sentence, in which certain words/phrases may be highlighted as follows:

- Text spans that are to be *included* (i.e., they should be annotated within the given category) are denoted using square brackets (e.g.: **[lorry driver]**). Cases that particularly exemplify cases in focus are emboldened; other words phrases that also belong to the category in question are also enclosed in square brackets, but are not emboldened.
    - ***Italicised underlined words*** may be used to emphasise cases in which particular words should be included within bracketed spans
- Text spans that are to be *excluded* (i.e., they are NOT to be annotated within the given category) are denoted using strike through (e.g.: ~~**[car make]**~~)

The remainder of these guidelines is organised as follows:

- Firstly, a *General Guidelines* section provides a set of scope and span guidelines that should be followed for the annotation of all entities (i.e., regardless of the specific entity category).
- This is followed by a separate section for each entity category, providing detailed category-specific annotation guidance.
- Finally, a set of quick reference guides is provided. These are one-page guides that aimed to act as mnemonics for both the general and category-specific guidelines during the annotation process. Once the full guidelines have been read and understood, these reference guides can be consulted easily as a reminder of the inclusion and exclusion criteria for scopes and spans.

2. General Guidelines

2.1 Annotation Scope

✓ Include:

- If the same entity is mentioned multiple times in a document, then EVERY mention should be annotated

The EC samplers were placed near the breathing zone of the **[parking ramp attendants]** and close to the open window. There were several **[parking ramp attendants]** that worked in the booth throughout the day

- Relevant abbreviations/acronyms should always be annotated <u>**in addition to, and separately from, full forms**</u>.

The sum of the [OC] fraction and the [EC] fraction is termed **[total carbon]** (**[TC]**).

- For entities that are nouns, consider the **complete** noun phrase first (i.e., all nouns and adjectives in the phrase)
    - If the complete phrase corresponds to an entity (according to the category-specific guidelines below), then the phrase should be marked up using this category, even if a part of the phrase could be seen to belong to another entity category
        - In the example below the phrase "underground construction workers" corresponds to a phrase of type *Occupation /Job Title*, according to the guidelines specified in section 3.2. Although "underground construction" corresponds to an *Industry/Workplace*, the longer *Occupation/Job Title* phase should be annotated in preference.

Personal exposures to dust and gases were measured among 189 **[underground construction workers].**

- If the complete phrase does not correspond to an entity, but a part of it does, then this part may be annotated.
  - In the example below, the complete noun phrase "underground tunnel construction project" does not correspond to an entity of interest, although "underground tunnel construction" complies with the guidelines for "Industry/Workplace" and so it should be annotated.

We monitored occupational exposure to diesel exhaust in an **[underground tunnel construction]** project.

✗ Exclude

- The annotation of longer phrases takes priority over the annotation of shorter phrases.
  - If, by following the category-specific guidelines, an annotation would include within it an annotation belonging to another category, then the shorter "embedded" span should NOT be annotated.
    - In the example below, since *"underground construction workers"* corresponds to an entity of type *Occupation / Job Title* (see section 3.2), the phrase *"underground construction"* within it (which corresponds to an *Industry / Workplace*, section 3.1) should not be annotated.

Personal exposures to dust and gases were measured among 189 **[[~~underground construction~~] workers].**

- As a further example, the phrase "*interstate highway*" in the sentence below could correspond to an *Industry/Workplace* (see Section 3.1), because it denotes where work is taking place. However, the word *"repair"* introduces a job activity and, according to the *Annotation Span* guidelines for the category *Job Task/Activity* (see section 3.3.3), the annotated span should include the object to which the *"repair"* action is being applied (in this case, the interstate highway). Therefore, the longer *Job Task/Activity* span "*repair of an interstate highway*" takes priority over the shorter *Industry/Workplace* span.

Samples 16–24 were obtained during **[repair of an ~~[interstate highway]~~]**

2.2 Annotation Span

Annotation spans should generally be kept as short as possible, but should include a sufficient number of words to characterise the entity according to the category-specific guidelines.

✓ Include:

- For entities that are **nouns**, the annotation will usually include all nouns and adjectives within the noun phrase, <u>as long as the complete phrase corresponds to an entity of interest</u>, according to the category-specific guidelines detailed in the sections below.

The **[*<u>asphalt</u>* strippers]** had a significantly higher exposure to total dust

Sixty **[*<u>underground coal</u>* miners]** exposed to diesel emissions were tested before and after a work shift for ventilatory function changes.

The elemental carbon (EC) levels in the **[*<u>trailing</u>* locomotives]** tended to be greater than those in the **[*<u>lead</u>* locomotive].**

- Entities that correspond to or include **verbs** usually describe actions or activities. Spans that include verbs or verb nominalisations should be annotated as described in the category-specific guidelines. If an annotation contains a verb, any preposition or adverb that follows the verb or its grammatical object should be included within the annotated span if the action/activity can only be fully understood in the presence of the preposition or adverb.

They send a P&D driver to **[pick the freight *<u>up</u>*],** which is often on pallets

For larger jobs a clamshell-type device may be used with a backhoe to **[lift the block *<u>out</u>*]**

- Each entity in a list should be annotated **<u>separately</u>**.

**[paver operators]**, **[screedmen], [roller drivers]**, and **[drivers of the binding agent trucks]**

- If a general entity is followed by more specific instances, both the general term and the more specific terms should be annotated **<u>separately</u>**.

**[Amines]** (**[*<u>dietanolamine</u>*]** , **[*<u>diethylenetriamine</u>*]** , **[*<u>triehytlenete- tramine</u>*]**) were collected on tubes filled with the adsorbent XAD-2

- Entities separated by slashes should be annotated **separately.**

All **[EC]**/**[OC]**/**[TC]**, [BC] and [PM 2.5] results were classified using environmental and occupational variables

✗ Exclude

- Determiners (e.g., *a, the, some*, …) and numerical values that occur at the start of noun phrases.

**[~~The~~ asphalt strippers]** had a significantly higher exposure to total dust

In total, **[~~22~~ miners]** were studied.

- The word "to" should be excluded when annotating a verb in its infinitive form.

Collectors usually stay in the rear of the truck [**~~to~~ dump the trash**]

- If an annotation contains a verb, subjects of the verb will typically be excluded from the annotated span. Note that objects of verbs may sometimes be included, based on instructions in the category-specific guidelines.

Next, one or more **~~[long-haul drivers~~ drive**] to the regional hub terminal closest to the delivery point.

2.3 Discontinuous Spans

Discontinuous annotations (i.e., annotations that include two or more conjoined, but non-adjacent "fragments" of text) should be used under certain circumstances, as explained below.

✓ Include:

- In some cases, words are omitted but are "implicitly" understood, as in the case of the coordinated phrase "*elemental and organic carbon"*
  - Even though the word *carbon* only occurs once, we can understand that two different substances are mentioned here:
    - *elemental carbon*
    - *organic carbon*

We want to identify these substances using two separate annotations. While *organic carbon* is a continuous annotation, *elemental carbon* is marked up as a discontinuous annotation that links together the two words of the phrase, even though they do not occur next to each other.

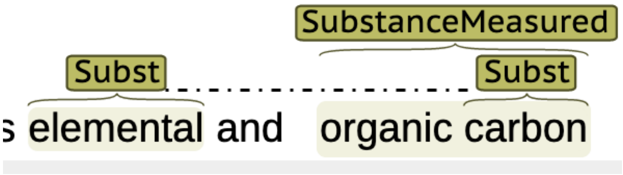

- A similar case holds in the example below. We can identify three different annotations that meet the criteria for being annotated using the *Industry / Workplace* category, i.e.,
    - *lead locomotives*
    - *trailing locomotives*
    - *miscellaneous yard locomotives*

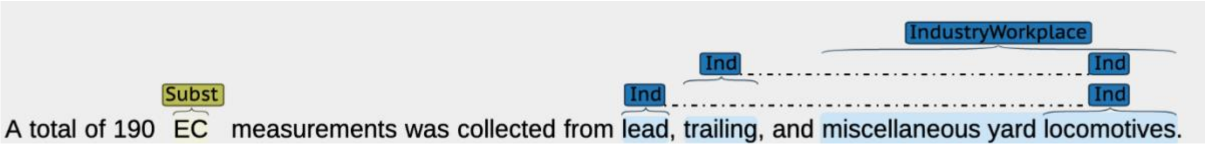

Again, we can make use of discontinuous annotations to account for the fact that the word *locomotives* is shared by all three of the workplaces.

- In the example below, discontinuous annotations are used to separately annotate the two different *Job Task/ Activity* spans mentioned in the sentence, i.e.,
    - *machine harvesting of tree crops*
    - *machine harvesting of vegetables*

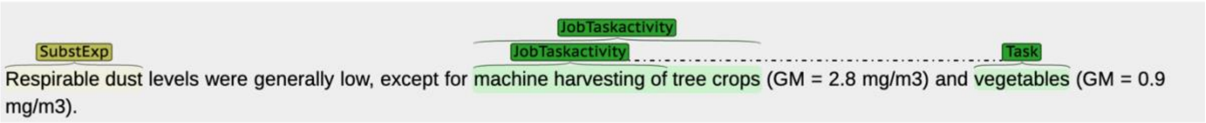

- Discontinuous annotations should also be used to exclude bracketed information that occurs in the middle of phrases. The bracketed information may correspond, among others to:
    - Manufacturers of devices or apparatus

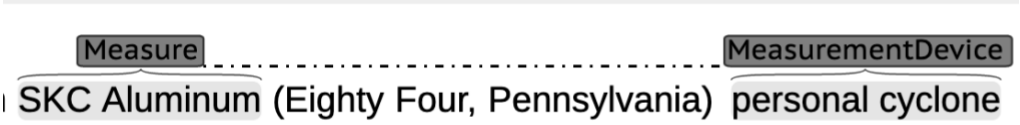

- Acronyms or abbreviations

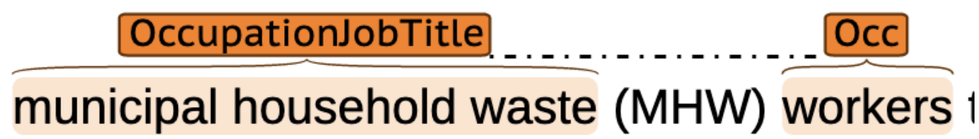

It should be noted that, based on the guidelines introduced above, acronyms should normally be separately annotated from full forms, when they correspond to entities of interest. In the example above, however, while the full form "*municipal household waste workers"* complies with the definition of the *Occupation /Job Title* category, the acronym "*MHW"* does not (since this represents *municipal household waste,* which is not itself an occupation)

✗ Exclude:

- For phrases that constitute entities, and which do not include bracketed information, discontinuous annotations should NOT be used to exclude words **in the middle of the phrases**; rather, the whole phrase should be annotated as a continuous annotation

✗ 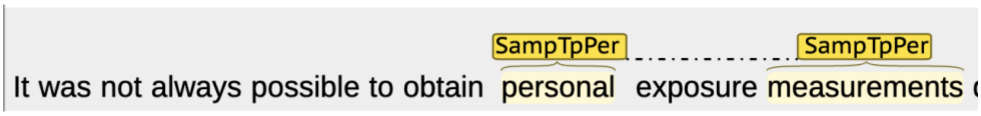

✓ 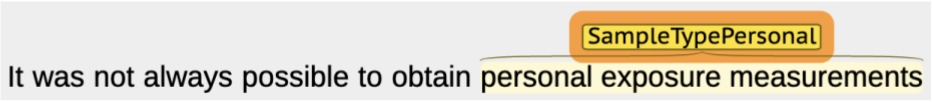

- The above guideline also holds if another entity occurs in the middle of a longer phrase; the longer phrase takes priority, according to the guideline above stating that **complete** noun phrases should be considered firstly

✗ 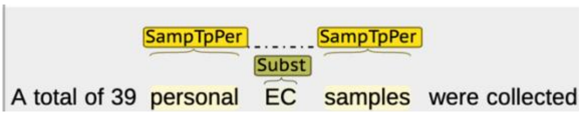

✓ 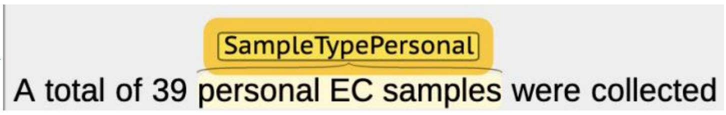

- Discontinuous annotations should NOT be used to link together separate continuous annotations, even if they are closely related to each other.
    - In the example below, *loader drivers* and *support operators* are two separate occupations in a coordinated phrase. The only case in which discontinuous annotations should be used for coordinated phrases is when one or more of the words is *shared* by multiple entities in the coordinated phrase, as was shown in the examples in the *Include* section above.

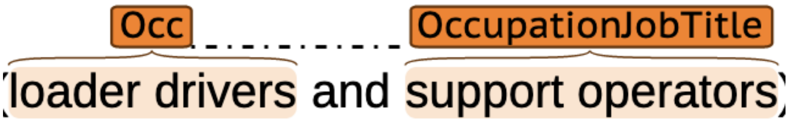

3. Category-specific guidelines

In this section, we provide definitions and guidelines for each of the six different entity categories.

3.1 Industry / Workplace

3.1.1 Definition

Any word or phrase that describes **EITHER**:

- The **industry** involved in the sampling series. This includes any description referring to an economic activity, such as the construction of buildings, extraction and processing of raw materials, the manufacturing/production, selling, purchasing, distribution or consumption of goods or the provision or selling of services.

- The type of **workplace/environment** involved in the sampling series. Such descriptions include large areas such as production plants, general environmental conditions such as “indoor”, “outdoor”, “underground” or “overground”, as well as more specific locations such as parts of buildings, enclosed spaces, and vehicles or other types of heavy equipment (both mobile and stationary) on which work takes place.

3.1.2 Annotation scope

✓ Include:

- Words/phrases that describe the economic activity, industry or sector to which the workers or workplaces participating in the sampling series belong.

We monitored occupational exposure to diesel exhaust in a Swedish **[underground tunnel construction]** project.
However, the long-term benefits and cost effectiveness of this treatment in **[mining operations]** have yet to be established
A similar approach was used in a study of diesel exhaust exposures in the **[trucking industry].**

7 companies were involved in **[nonmetal mining]**, 2 in **[railway transport]**, and 7 in **[bus transport]**.

Job occupations found in the **[mechanical services]** division include pipe fitters, machinists, welders and mechanics

The **[construction]** activities studied included: abrasive blasting, concrete pavement sawing and asphalt milling

- Words/phrases that define/name the type of workplace or environment involved in the measurements. This may include (but is not limited to) the following:

    - References to general areas, plants or factories

The main objective of this study was to assess the occupational exposures of gate controllers at the **[port]** of Montreal, Canada, to DE from on-road container trucks following a multimeric monitoring strategy.

Measurements were carried out on one **[bitumen plant]**, three **[asphalt production plants]**, two **[asphalt stripper sites]**, and 14 **[asphalt paving sites]**.

Exposures also were assessed at 4 **[small rural terminals]** in New England by sampling in the **[dock area]**

The exposure assessment survey in the **[diesel factory]** was conducted from October 2012 to March 2013.

    - Indoor areas where work takes place.

The possibility of adverse respiratory health effects amongst coal miners in and at **[diesel-use mines]** has been investigated.

Paving in **[tunnels]** showed significantly higher levels of total dust

The Latvian **[railway depot]** we studied included a large **[repair and maintenance workshop]**

All **[tollbooths]** are of similar size (L ×W×H= 1.5 m ×1.0 m× 2.1 m)

- Specific types of outdoor areas

Particle number concentration as measured by P-Trak at a **[rural road paving site]** (about 60 km from Stockholm) on a **[four- lane motorway]** is shown in Figure 1.

Two areas of potential diesel exhaust exposure were identified: the **[turnaround area]** and the **[heavy repair area]**.

- Vehicles and other types of "mobile" heavy equipment
  - i.e., equipment which workers sit in or on to operate, and which can generally be moved.

This study reports parallel measurements of airborne levels of diesel fume pollutants in nine [distribution depots] where **[diesel powered fork-lift trucks]** (**[FLTs]**) were in use.

The elemental carbon (EC) levels in the **[trailing locomotives]** tended to be greater than those in the **[lead locomotive].**

A number of measurements in the [ramps] and **[buses]** were close to or below the limit of detection ~1 mg m 3)

A heavy equipment operator is stationed in the cab of the **[backhoe]**.

The water truck driver spent almost all of his time in the cab of the **[water truck]**.

The operator spent almost all of his time on top of the **[asphalt mill].**

- Pieces of stationary heavy equipment that are sufficiently large to constitute a point of reference to specify the location of workers.

In Figure 2, a laborer is seen stationed at the **[pneumatic drills]**.

During the first shift, the investigators found 8-hour TWA respirable quartz concentrations ranging from the REL to over 4 times the REL at Area 1 above the **[conveyor belt]**.

- References to specific features of the environment where work takes place, which may impact on the types and/or levels of exposures (e.g., underground, overground, enclosed, unenclosed, indoors, outdoors etc.)

Multiple samples were collected from three areas on the **[surface]** during two consecutive day shifts.

Multiple samples were collected during three day shifts in three **[underground locations]**

The levels for elemental carbon ranged from 4 to 178 g/m3 (mean of 41 g/m3) inside **[enclosed work spaces]**, compared with 0.5 to 53 g/m3 (mean of 10 g/m3) for samples collected in **[unenclosed work sites].**

Occupational Exposure to Respirable Dust, Respirable Crystalline Silica and Diesel Engine Exhaust Emissions in the London **[Tunnelling Environment].**

For the inhalable dust fraction, levels were reduced considerably when an **[enclosed cabin]** was present.

Samples 1–3 were obtained during sandblasting of a concrete wall **[outdoors].**

- Descriptions of simulated workplaces

Initial test work was carried out in a **[test tunnel]** (45 m long, 5 m wide, and 2.5 m high) constructed on pit top from steel formwork, reinforced plastic sheeting, and brattice to create a **[simulated section of mine roadway].**

✗ Exclude:

- Specific tasks performed by workers on a daily basis; these should instead be annotated using *the Job task / Activity* category (see section 3.3)

This resulted in part because some of them were exposed to particulate matter generated by **[~~welding~~]**.

The highest mean thoracic quartz exposures were found in **[~~laying conduit~~]**.

- Phrases that form part of job task descriptions, even if these phrases could also correspond to workplaces.
    - In the example below, the word *reconstruction* introduces a job task. The span for the *Job Task/Activity* annotation should include the object undergoing

reconstruction, i.e., *"interstate highway bridge"* (see *Annotation Span* guidelines for *Job Task/Activity* in Section 3.3.3).

  - Thus, the *Job Task/Activity* annotation should be *"reconstruction of an interstate highway bridge"*
  - Although the bridge is where the work is taking place, the general guideline that the annotation of longer spans takes priority means that *"interstate highway bridge"* should not be annotated as an *Industry/Workplace* in this context.

> Samples 13–15 were obtained during *reconstruction* of an **[~~interstate highway bridge~~].**

- Mentions of smaller (e.g., hand-held) pieces of equipment that do not provide information about the location of workers

> Tables III and IV present data from a [construction site] where the concrete walls of a large building were being smoothed using an **[~~electric angle grinder~~]**.

> For small jobs, a **~~[jackhammer]~~** may be used to break up the heavy block of sawed concrete

- Names of locations (towns, countries, etc)

> All the SO2 was absorbed or neutralised in the office at **~~[Dalston]~~**, and only the dock site showed any excess over background.

- References to named sites or areas

> At **~~[Merton]~~** all these instruments were housed in an office alongside **~~[site A]~~**

> During the second shift, quartz was detected in the **[~~Area 1~~]** samples, although at levels too low to quantify.

- General words/phrases that do not provide information about the nature of the working environment and/or industry

> Two or more additional diesel sources in the **~~[area]~~** will elevate the concentration of elemental carbon, as will enclosing the **~~[work site].~~**

Two areas of potential diesel exhaust exposure were identified at each **~~[yard]~~**

Each **~~[plant]~~** was sampled twice in different seasons to evaluate seasonal and process-related changes in exposure

A laborer spent his time walking alongside the **~~[machine]~~**, as did the foreman

- Words/phrases that occur as part of company names, even if they could otherwise denote workplaces.

Other participants in the exposure monitoring phase included *Ontario Southland* **~~[Railways]~~**

Potential improvements were validated by application in standard coal mining operations at *Tower* **[~~Colliery~~].**

- Words/phrases that describe very general features of the environment, rather than providing information about the specific working environment.

The levels of both NO2 and particles were higher for bus drivers in the **[~~city~~]** than for them driving in the **~~[suburbs]~~.**

- Words or phrases describing specific job roles; these should be annotated as Occupation/Job Title (see Section 3.2)

A total of 51 **~~[asphalt workers]~~** carried personal samplers

- Parts of phrases that characterise workers (even if the characterisation is based on the industry/location in which they work). The complete phrase should instead be annotated as *Occupation/Job Title* (see Section 3.2)

Personal samples for exposure to dust, diesel exhaust, quartz, and welding fume were collected on **~~[highway construction]~~** *workers*.

Before 1995 the number of **[~~mine~~]** *workers* exposed to diesel exhaust was greater

Workers on the ~~***[farms]***~~ were asked to wear a personal sampler during various operations on the farms

Filling of pallet boxes with SiC resulted in a 1.6–2.7-fold increased exposure for *operators in the* ~~***[refinery]***~~.

3.1.3 Annotation span

✓ Include:

- Words within the same phrase that characterise the workplace more precisely, in terms of its location, size, usage, characteristics, etc.

As before, the years of diesel exposure relates only to that member of the pair who worked in an **[*underground diesel-use* mine]**

The technical problems with sampling for EC as an indicator for diesel exhaust in **[*coal* mines]** are well known

Also, for a **[*small, poorly ventilated* workroom]**, the general ventilation multiplier would be similar

Approximately 12 manual workers were usually deployed in the vicinity of the **[*diesel-powered* loaders]**

- Words like *industry* or *services,* if they occur in the same phrase that mentions an industry or economic activity.

[Engineering *services*] is also responsible for constructing non-railway buildings such as storage sheds and bunk houses

The purpose of this study was to assess diesel exhaust exposures in the **[electric utility *industry*]**

- Words like *site* or *environment,* if they help to fully characterise a place of work.

Ninety tunnel construction workers employed at 11 available **[construction *sites*]** participated in the exposure study

Table II presents data from two **[interstate highway construction *__sites__*]**.

Occupational Exposure to Respirable Dust, Respirable Crystalline Silica and Diesel Engine Exhaust Emissions in the London **[Tunnelling *__Environment__*]**

✗ Exclude:

- References to named places.

**[Mine ~~M~~]** was located in Utah at an altitude of approximately 2,400 m (8,000 ft)

Occupational Exposure to Respirable Dust, Respirable Crystalline Silica and Diesel Engine Exhaust Emissions in the **[~~London~~ Tunnelling Environment].**

- Words that do not constitute part of the industry name/description

Drivers constituted a large proportion of the workers in **[bus transport ~~companies~~]**

3.2 Occupation / Job Title

3.2.1 Definition

A phrase that characterises a person or group of people in terms of their occupation, job title, position, the general type of work that they typically undertake, the types of equipment/materials with which they typically work or the type of location in which they typically work.

3.2.2 Annotation scope

✓ Include:

- Any word or phrase that characterises a person or persons according to the type of work that they typically undertake, the types of equipment/materials with which they typically work or the type of location in which they typically work. This includes (but is not necessarily limited to):
    - Specific job titles

The geometric mean exposure to nitrogen dioxide was 0.9 ppm for **[electricians]**.

The study primarily focused on operating engineer, and to a lesser extent on **[carpenters]** during the tunnel finish and cut and cover stages.

- Phrases that characterise people according to the nature of their work

The **[asphalt strippers]** had a significantly higher exposure to total dust

The main objective of this study was to assess the occupational exposures of **[gate controllers]** at the port of Montreal, Canada, to DE from on-road container trucks following a multimeric monitoring strategy.

- Phrases that characterise people according to the materials with which they work

The prevalence of sneeze was higher among the **[*battery* workers]**

In addition, **[*concrete* workers]** performing ironwork and carpentry work outside the tunnels were included in the study

- Phrases that characterise people according to the general location or environment in which they work

Before 1995 the number of **[*mine* workers]** exposed to diesel exhaust was greater

This suggests that diesel exhaust exposures of **[*railway* workers]** in Russia currently are the same as exposures in most Western countries.

The **[*underground* workers]** were a priori expected to have 'high' exposure to diesel

The **[*surface* workers]** were anticipated to experience 'low' (background) levels of exposure

[Workers *in toll stations*] are exposed to high levels of both elemental and organic carbon exhaust

Filling of pallet boxes with SiC resulted in a 1.6–2.7-fold increased exposure for **[operators *in the refinery*]**.

- Phrases that characterise people according to the equipment or vehicles that they generally operate

A [***heavy equipment*** **operator]** is stationed in the cab of the backhoe.

The investigators found a respirable concentration of quartz for the **[*concrete cutter* operator]** of 14.0 mg/m 3 during a 350-minute sample.

In fact, each bus route would have several **[*bus* drivers]** in different buses throughout the day

The [helper] used a new, clean, pesticide-type spray can filled with tap water to wet down the surface of the concrete wall just ahead of where the **[worker *using the grinder*]** was working

- Phrases that characterise people according to the tasks that they undertake as part of their work

Underground construction workers were divided into occupational groups performing similar tasks in similar working conditions: **[*drill and blast* crew]; [*shaft-drilling* crew];**

- Phrases that characterise people according to the generic activity (industry) in which they work

Personal air measurements of aerosols and gases among **[*tunnel construction* workers]** were performed as part of a 11-day follow-up study

Personal exposures to dust and gases were measured among 189 **[*underground construction* workers]**

✗ Exclude:

- Any word or phrase describing the nature, character, location or industry of work that does NOT refer to specific people; such phrases should normally be annotated either as *Industry /Workplace* (see section 3.1) or *Job Task / Activity* (see section 3.3)._

Exposure to Ultrafine Particles in ~~[Asphalt Work]~~

Briefly, the excavation process starts off with [~~drilling~~]

- Phrases that characterise workers in ways other than according to the nature of their work, e.g., according to shift patterns

~~**[Workers on the afternoon and night shifts]**~~ were generally younger and had worked about 6 years less than the ~~**[day shift workers]**~~.

- General phrases that do not correspond to a specific occupation or type of job

**[~~miscellaneous trades~~]** including [ironworkers], [carpenters], [plumbers], [electricians], [t ile setters], [piledrivers], and [boilermakers]

Elemental carbon (EC), nitrogen dioxide and inhalable dust were measured using sampling equipment attached to the subjects and measurements obtained in the breathing zone of the ~~**[workers**~~]

- Words/phrases that do not refer to a worker in the context that they occur, even if the same word/phrase may refer to workers in other contexts .
    - In the example below, the word *grinder* is clearly referring to a piece of equipment and so should NOT be annotated.

A metal shroud was attached to the ~~**[grinder]**~~, and a flexible tube connected the vacuum to the shroud.

-
    - Note that in other contexts (such as in the example below), *grinder* may also refer to a worker operating such equipment; in such cases, the *Occupation /Job Title* category should be used.

Separate personal breathing zone samples were obtained for the **[grinder]** and for the helper.

- Mentions of workers who do not constitute subjects of exposure measurements (i.e., those who are not being sampled).
    - E.g., industrial hygienists who carry out the study

The ~~**[industrial hygienist]**~~ did a walk through survey of the premises and recorded information such as type of equipment used and organization of work.

3.2.3 Annotation span

✓ Include:

- All nouns and adjectives that occur within *simple* noun phrases that characterise workers. Simple noun phrases consist of one or more nouns, and possibly adjectives. The different words in the phrase may help to precisely define the type or location of work, as in the examples below:

The **[*shaft* drillers]** were exposed to the highest air concentrations of thoracic aerosol mass
Sixty **[*underground coal* miners]** exposed to diesel emissions were tested before and after a work shift for ventilatory function changes.
The only job with potential expo- sure to diesel exhaust in which a sizable number of women were employed was **[*bus* driver]**

- Longer, *modified* noun phrases may be annotated if they convey essentially the same meaning as a simple noun phrases, such as those provided in the examples above. For example, the phrase ***worker using the grinder*** is an example of a modified noun phrase that can be used to describe the same type of worker as the simple noun phrases ***grinder*** or ***grinder operator***.

  Modified noun phrases consist of:

  - A noun corresponding to a worker or group of workers
  - A subsequent *modifying* phrase, which serves to better characterise the workers in terms of their typical type or location of work. The modifying phrase may take the following forms:

    - *A prepositional phrase*
    - *A phrase beginning with a verb in "ing" form*
    - *A relative clause* introduced by the word "who"
    - *A reduced relative clause,* i.e., a shortened relative clause in which the word *"who"* is omitted.

    Whenever a noun corresponding to a worker is encountered, its context should be carefully considered to determine whether the annotation of a longer modified phrase is appropriate.

    The table below provides examples of how information conveyed in simple noun phrases that characterise workers can be expressed in (near) equivalent ways using different types of modified noun phrases. In each case, the

complete span of the modified noun phrase should constitute the span of the *Occupation/Job Title* annotation.

- It should be noted that some of the example *modified* noun phrases provided in the table below contain phrases that could be annotated using other categories if they occurred in other contexts (e.g., *"asphalt mill"* could be *Industry/Workplace,* while *"processing SiC"* could be a *Job Task/Activity).* However, according to the general guideline that the annotation of longer phrases takes priority over shorter phrases, the complete modified noun phrase should be annotated as *Occupation/Job Title* in these cases.

Simple noun Phrase	Modified noun phrase with (near) equivalent meaning	Modifying phrase type	Example sentence
train drivers	*drivers of trains*	Prepositional phrase	In the coal mine 1-NP levels in the breathing zones of the **[drivers *of trains*]** were ~2-fold higher than the levels observed for surface workers
toll station workers	*workers in toll stations*	Prepositional Phrase	**[Workers *in toll stations*]** are exposed to high levels of both elemental and organic carbon exhaust
backhoe operator	*operator of the backhoe*	Prepositional Phrase	The [laborer at the controls of the drill] works in higher dust concentrations than the **[operator *of the backhoe*]**
farm workers	*workers on the farms*	Prepositional Phrase	**[Workers *on the farms*]** were asked to wear a personal sampler during various operations on the farms.
asphalt mill operator	*workers operating asphalt mills*	*"ing"* clause	The **[workers *operating an asphalt mill*]** removed old asphalt from an interstate highway.
grinder operator	*worker using the grinder*	*"ing"* clause	The [helper] wet down the surface of the concrete wall just ahead of where the **[worker *using the grinder*]** was working
SiC processors	*workers processing SiC*	"ing" clause	The group consisted of **[workers *processing SiC*]** ([crusher operator], other [refinery operator], and [fines operator])
SiC production workers	*workers who are employed in the production of SiC*	Relative clause	Crystalline silica exposures for **[workers *who are employed in the production of SiC*]** were generally low.
SiC production workers	*workers involved in the production of SiC*	Reduced relative clause	The group consisted of **[workers *involved in the production of SiC*]** ([mix operator] and [pay loader operator]).

✗ Exclude:

- Information following noun phrases, corresponding to specific locations or conditions under which workers' activities are taking place, if the noun phrase itself provides sufficient characterisation of the job or occupation.

NOTES:

- As specified in the *Include* criteria for annotation scope, *Occupation /Job Title* annotations may include certain types of general locations, if they are the only means by which workers are characterised (e.g., ***mine*** *workers*, ***underground*** *workers*). In the example below, the phrase *paver operators* provides a sufficient characterisation of workers, and so additional information should be excluded from the span
- Locations of work should normally be annotated using the *Industry/Workplace* category

**[Paver operators ~~seated on paving machines without a cabin]~~** showed significantly higher levels of total dust than **[paving operators ~~on paving machines with a closed cabin]~~**.

- Words referring to named places

Raffle (1957) has studied the deaths, retirements due to ill health, and transfers to alternative work due to lung cancer amongst various groups of **[~~London~~ Transport employees]**

- Words referring to personal characteristics of workers (e.g., gender or ethnicity)

A quasi-experimental study design was developed to examine the acute effects of occupational exposure to diesel emissions in **[~~white male~~ underground coal miners]** during an 8-h work shift.

- Job codes in occupational schemes

The feasibility study focused on workers with potentially high exposure to diesel emissions: **[drivers ~~(ISCO 985)~~]**, **[mechanics ~~(ISCO 843)~~]**, and **[railway workers ~~(ISIC 71)~~]** in land transport

- Verb clauses
  - "Active" verb clauses describe *actions/activities* rather than people and should not be annotated as *Occupation /Job Title*

The [~~worker operated a water truck~~].

  - Since *Occupation/Job Title* annotations name or characterise ***people,*** and are described using *nouns,* it follows that *Occupation/Job Title annotations* should

always correspond to *noun phrases* (either *simple* or *modified* noun phrases, as exemplified above).

- A potential source of confusion is that some types of modified noun phrases include verbs, but these serve to *characterise* the worker, e.g.,
    - *worker **operating** the water truck*
- The difference between modified noun phrases and verb clauses is that, in a sentence, modified noun phrases function in the same way as simple noun phrases, but verb clauses do not. For example, if the subject of a verb is a simple noun phrase, then this subject phrase can be straightforwardly substituted with a modified noun phrase, even if the modified noun phrase contains a verb. This is illustrated below.

The **[water truck operator]** was exposed to higher levels of dust

The **[worker operating the water truck]** was exposed to higher levels of dust

- In contrast, substituting the simple noun phrase in the example with the verb clause "worker operated a water truck" would result in a sentence that is ungrammatical.

The **[~~worker operated a water truck~~]** was exposed to higher levels of dust

3.3 Job Task / Activity

3.3.1 Definition

Specific and well-defined physical activities or actions that are carried out by workers as part of their daily working duties.

Annotations in this category should aim to capture **as much information as is provided in the text about the activity itself** that is conveyed by:

- The word (noun or verb) that corresponds to the basic activity
- Other words/phrases that occur in the immediate context of this word, which serve to specify/clarify the activity more precisely (if present)

As an example, the word "*sandblasting*" may be used to describe a basic activity. If this is followed by information about ***what*** is being sandblasted, then this information should also be included within the annotation, e.g. *"sandblasting of a concrete wall".*

Annotations in this category should only be created if the annotated text span in isolation is sufficient to allow a specific and well-defined activity to be understood.

The examples in the *Annotation scope* and *Annotation span* sections below serve to clarify more precisely when annotations of this category should be created and what should be included within their spans.

3.3.2 Annotation scope

✓Include:

- Single words (verbs or nouns) may be annotated, but ONLY if they correspond to specific and well-defined job activities that can be understood in isolation, and as long as no further neighbouring details about the nature of the activity are provided in the text.
  - Words such as *sandblasting, welding* or *drilling* or provide a clear, well-defined picture of a basic activity, even if there are no neighbouring details in the text about *what* is being sandblasted, welded or drilled. However, details about the objects or materials undergoing such actions should ALWAYS be included in the annotated span, IF they are present in the text.
  - Words such as *installation* or *repair* are too vague in isolation to convey a well-defined activity. However, if such words are followed by information about *what* is being installed or repaired e.g., "*repair of highways*" then the nature of the task becomes sufficiently clear and the complete span does constitute a valid *Job Task/Activity* annotation.

This resulted in part because some of them were exposed to particulate matter generated by **[welding]**.

The operations with the highest mean respirable dust exposures were found in **[~~installation~~].**

- Longer phrases should be annotated whenever they are present in the text and as long as they provide further details about what the activity involves. Further guidance about the length of span to annotate in such cases is provided in the *Annotation Span* section below.

In the thoracic dust samples, the highest percentage of quartz was found in the samples from **[*concrete* pouring]**.

[loading *of debris*] by means of a diesel-powered loading machine on to three to four diesel engine trucks

The highest mean thoracic quartz exposures were found in **[laying *conduit*]**.

Lower but still considerable levels were measured for **[*ground preparation* operations]**, **[*mechanical* mowing *of weeds*]** and **[feeding *poultry*]**.

✗ Exclude:

- Phrases that describe **where** work was carried out, _as long as_ these locations DO NOT correspond to object to which the activity is applied, or which is affected by it.
    - In the example below, the phrases with strikethrough denote locations where measurements were taken. As such, they should be annotated using the *Industry /Workplace* category (see Section 3.1)

> Measurements were carried out on one **~~[bitumen plant]~~**, three **~~[asphalt production plants]~~**, two **~~[asphalt stripper sites]~~**, and 14 **~~[asphalt paving sites]~~**

-
    - In contrast, consider the example below. Although the word *"highway"* could be annotated as *Industry/Workplace* in certain contexts, here it constitutes part of a job task description (i.e., what is being repaired), and so the complete emboldened phrase should be annotated as *Job Task/Activity.*

> Samples 5–12 were taken during **[repair of a *highway*]**.

- Parts of phrases that **characterise workers** (even if the characterisation is based on the type of task that they undertake). The complete phrase should instead be annotated as *Occupation/Job Title* (see Section 3.2)

> Underground construction workers were divided into occupational groups performing similar tasks in similar working conditions: **[~~drill and blast~~] crew; [~~shaft-drilling~~] crew;**

- Phrases which start with a verb, but which denote locations of work, rather than specific activities; the locations instead should be annotated using the *Industry/Workplace* category (see Section 3.1)

> Area samples were collected in an office, to evaluate employees **[~~working indoors in laboratory and office environments~~]**

> Dock workers **[~~use forklift trucks~~]** to [move freight around]

- Verbs/nouns that refer to work carried out, but which are too vague/general to allow an understanding of the specific task undertaken, and which do not have neighbouring words/phrases that specify further information about the task

> Workers were engaged in various **~~[production]~~** assignments above ground

The **[handling]** would be described by the way in which the engine was being [driven]

The measurements showed considerable difference in exposure levels between **[various operations]**

- In the example below *"construction activities"* should not be annotated as *Job Task/Activity.* Although the phrase refers to a set of activities, this is at a very general level that **does not** comply with the definition of this category, i.e., "*specific and well-defined physical activities or actions*". Note that:
  - The other activities listed in the sentence **do** correspond to specific, well-defined tasks, and so they **should** be annotated.
  - It should also be noted that the word "construction" is a candidate for annotation as *Industry/Workplace,* since it refers to the economic activity that results from aggregating specific job tasks such as the ones listed in the sentence.

The **[construction activities]** studied included: [abrasive blasting], [concrete pavement sawing] and [asphalt milling]

- Unintended actions/activities, or an activity that does not form part of a worker's daily duties or activities

Each employee who participate in the study **[completed an activity questionnaire]** at the end of the work shift.

- Words/phrases that characterise a person or persons in terms of the job that they do, even if they are characterised in terms of the tasks that they undertake. Such phrases should instead be annotated as *Occupation/Job Title* (See section 3.2)

The **[asphalt strippers]** had a significantly higher exposure to total dust than the other **[asphalt workers]**

- Phrases that describe **general** economic activities or industries rather than specific job tasks; these should be annotated as *Industry / Workplace* (see Section 3.1)

Both personal and area samples were collected from three major operating divisions of the railways: **[mechanical service], [transportation]** and **[engineering].**

3.3.3 Annotation span

✓Include:

Annotations should include within their span as much information as is provided in the text about the activity *itself*.

Descriptions of activities may vary in terms of:

- The ***types of information*** provided about the activity
- The ***level of detail*** provided about the activity
- The ***structure of the description***, i.e., the types of phrases used to convey information

The annotated span should aim to capture as much information as is provided about **what** the activity involves. Typically, the *types of information* provided in text will include the following:

- *Nature or characteristics* of the activity
- Materials, items or objects to which the activity *is applied or which are affected by it*
- *Initial and/or resultant state* of the activity, e.g.
    - A starting or finishing position of the activity
    - Materials, items or objects which *result from* or *constitute the outcome* of the activity

As mentioned above, the *level of detail* provided about activities in text can vary, in that information may be provided that corresponds to some, all or none of the types listed above.

In the examples below, the parts of the annotated span corresponding to different types of information are indicated using curly brackets, with subscript words denoting the type of information that they provide.

As an example of how the level of detail provided about activities in text may vary, consider the activity of *drilling,* which may be defined as follows:

The activity of boring with a drill into or through a material [item affected] to produce an outcome (e.g., holes, grooves) [resultant state].

- In some cases, no details are provided about the item affected or the resultant state. In the example below, only the word corresponding to the basic activity, i.e., *drilling,* is provided. However, since *drilling* alone conveys a well-defined activity, this single. Word still constitutes a valid *Job Task/ Activity* span.

Typically, this **[{drilling$_{activity}$}]** is done without any dust control**.**

- In other cases, partial details about the activity are provided, which should be included within the annotated span whenever they are present.

- In the example below, the word *shaft* corresponds to the result of the drilling activity. Details of the affected object (i.e., the material into which the drilling took place) are not provided.

> **[{Shaft$_{result}$}{drilling$_{activity}$}]** was the highest exposed job to air concentrations for all measured contaminants.

- The two examples below illustrate cases in which information about the material affected by the drilling is provided, but details about the result of drilling are not.

> Tunnel construction workers are engaged in **[{rock$_{item_affected}$} {drilling$_{activity}$}]**

> For **[{drilling$_{activity}$}** {**concrete highway pavement$_{item_affected}$**}**]** the respirable quartz concentrations ranged up to 4.4 mg/m 3 for a 358-minute sample.

- More complete details about the activity may also be described. In the example below, both the item affected by the drilling *and* the result of the activity are provided. Since both are integral to the description of the drilling activity, they should *both* be included within the annotated span.

> Another method is to **[{drill$_{activity}$} {holes$_{result}$} {through the block$_{item_affected}$}].**

Job tasks/activities may be conveyed by either *nouns* or *verbs*. Whenever a noun or verb conveying a relevant activity is encountered in text, different types of words/phrases in the immediately surrounding context may provide further details about the activity. The tables below list and exemplify the types of contextual words/phrases that should be considered for inclusion within annotated spans, depending on whether the basic activity is described using a noun or a verb.

Potential types of context for inclusion in annotations of job tasks described by *verbs*

Type of contextual word, phrase or construction	Example	Notes
**Grammatical object of verb**	*Lower but still considerable levels were measured for* ***[{feeding***$_{activity}$***} {poultry***$_{item\_affected}$***}]***	• Grammatical objects of verbs most typically specify the *item affected* by the activity
	Collectors usually stay in the rear of the truck to ***[{dump***$_{activity}$***} {the trash*** $_{item\_affected}$***}]***	
	For ***[{drilling***$_{activity}$***} {concrete highway pavement***$_{item\_affected}$***}]*** *the respirable quartz concentrations ranged up to 4.4 mg/m 3*	• Grammatical objects of verbs may be longer phrases, possibly including prepositions (e.g. , *the heavy block of sawed concrete*)<br>• The complete description of the item affected should be included in the annotated span
	For small jobs, a jackhammer may be used to ***[{break up***$_{activity}$***} {the heavy block of sawed concrete***$_{item\_affected}$***}]***	
**Prepositional phrase following verb**	*The activities involved* ***[{tunnelling***$_{activity}$***} {through areas of clay***$_{item\_affected}$***}]***	• The action of *tunnelling* involves forcing a passage through a material.<br>• The prepositional phrase following the verb provides details about the *affected* material.
**Prepositional phrase following direct object of verb**	*Another method is to* ***[{drill***$_{activity}$***} {holes***$_{result}$***} {through the block***$_{item\_affected}$***}]***	• The verb object (i.e., *holes*) corresponds to the *result* of performing the *drilling* activity.<br>• The subsequent prepositional phrase contains the item *affected* by the activity, i.e., through which drilling is taking place
	A small crane was used to ***[{lift***$_{activity}$***} {the concrete block***$_{item\_affected}$***} {out of the pavement***$_{initial\_state}$***}]***	• The activity of *lifting* involves raising an object from a starting position to an end position.<br>• The verb object corresponds to the item *affected* (i.e., *what* is being lifted).<br>• The subsequent prepositional phrase corresponds to the *initial state* of the activity, i.e., the starting position of the block.
**Activities in the passive voice**	*The heavy repair area, where the* ***[{locomotives***$_{item\_affected}$***} {are repaired***$_{activity}$***}]****, me 200 feet by 250*	• Activities may be described using the *passive voice*.<br>• The verb object in the active voice becomes the subject in the passive voice/<br>• The annotated span should include:<br>o Grammatical subjects and prepositional phrases that contribute towards the description of the activity<br>o The utilised form of the verb "to be" occurring together with the activity verb
	[{Wet concrete$_{item\_affected}$***} {was applied***$_{activity}$***}]*** *using a remote controlled nozzle application*	
	[{Expansion joints$_{result}$***} {were being sawed***$_{activity}$***} {in the fresh concrete***$_{item\_affected}$***}]*** *within six hours of being placed.*	• Prepositional phrases following passive verbs should also be included in the span if they provide information about the activity.<br>• The action of *sawing* involves applying a saw to a material (here, the *fresh concrete* specified in the prepositional phrase) to produce a *result* (here, the *expansion joints* specified as the subject of the passive construction).
	A worker used a gasoline-powered handheld masonry saw (see Figure 7) to [{saw$_{activity}$*} {indentations*$_{result}$*} {in the concrete floor*$_{item\_affected}$*}]so that* ***[{restroom floor drains***$_{affected}$***} {could be installed***$_{activity}$***}]***	• If a modal verb should as "could", "can"or "would" occurs in the same verb phrase as the basic activity verb, this should be included within the annotated span

Potential types of context for inclusion in annotations of job tasks described by *nouns*

Type of contextual word, phrase or construction	Example	Notes
**Other nouns/ adjectives in same noun phrase**	*The respirable dust and silica concentrations for the personal samples collected during* ***[{concrete$_{item_affected}$} {mixing$_{activity}$}]*** *were below occupational exposure limits*	• Other nouns within the same noun phrase as the basic activity may provide information about the item affected by the activity.
	Tunnel construction workers are engaged in ***[{rock$_{item_affected}$} {drilling$_{activity}$}]***	
	In the past they performed most major ***[{engine$_{item_affected}$} {repair$_{activity}$}]*** *on company trucks in house*	
	[{Shaft$_{result}$} {drilling$_{activity}$}] *was the highest exposed job to air concentrations for all measured contaminants.*	• Other nouns within the same noun phrase as the basic activity may alternatively correspond to the result of the activity
	Shaft drillers had the highest exposure to oil mists (GM=1.4 mg/m3), which was generated mainly from ***[{pneumatic$_{nature}$} {drilling$_{activity}$}]****.*	• Adjectives within the same noun phrase as the basic activity may serve to describe the nature or characteristics of the activity
	Mines M and R use ***[{continuous$_{nature}$} {mining$_{activity}$}]*** *to develop longwall panels.*	
**Prepositional phrase immediately following noun phrase**	*The excavation process starts off with* ***[{charging$_{activity}$} {of explosives$_{item_affected}$}]****.*	• If the activity is described using a noun, then following **prepositional phrases starting with "of"** will typically provide similar information to the grammatical object of a verb, i.e., such phrases will often provide information about the item(s) affected by the activity
	The operations with the highest mean respirable dust exposures were found in ***[{installation$_{activity}$} {of drop ceilings$_{item_affected}$}]***	
	Inhalable dust levels were lower during [{ground$_{item_affected}$} {preparation$_{activity}$}] and ***[{machine$_{nature}$} {harvesting$_{activity}$} {of dry harvested field crops$_{item_affected}$}]*** *when an enclosed cabin was present*	
**Subsequent following prepositional phrase**	*During the* ***[{milling$_{activity}$} {of asphalt$_{result}$} {from concrete highway pavement$_{item_affected}$}]****, the sampling indicated a respirable quartz concentration ranging up to 0.34 mg/m 3 for a 504-minute sample*	• Similarly to verbs, subsequent prepositional phrases that follow noun phrases may provide additional relevant details about the activity<br>• The activity of *milling* is applied to a starting material, which is *affected by* the action to produce a *resultant material*<br>o Here, the **first prepositional phase** contains the resultant material<br>o The **second prepositional phrase** contains the starting (affected) material

- Sufficient text should be annotated to adequately describe a task or activity
    - In the example below, *"concrete finish"* does not describe an activity, but *"concrete finish work"* does. A similar case also holds for *"excavation support work"*.

The operations with the highest mean respirable dust exposures were found in **[concrete finish *work*]** and **[excavation support *work*]**

- Distinct activities that are denoted to be temporally ordered (e.g., if one activity is specified to occur before, during or after another) should be separately annotated

The dust samples in Table II represent **[concrete drilling work]** during **[interstate highway repair]**.

- If a "higher level" well defined task is broken down into one or more specific tasks, then all tasks should be separately annotated

Rock support includes, e.g. **[sealing of the rock]** by **[spraying wet concrete onto the excavated surface]**

✗ Exclude:

- Descriptions of **where the activity was carried out** or **where items involved in the task were located.**
    - In the example below the *contents of containers* constitute items involved in the task of *inspection,* i.e., they are the items undergoing inspection. Details about *where* these containers were located should be EXCLUDED from the annotated span.

The task of the gate controllers consist of **[inspecting the contents of containers ~~found on containers trucks coming through the gate of the port terminals~~]**.

-
    - In the example below, the phrase *"partially enclosed truck bays"* corresponds to the location **where** the *activity* of loading trucks takes place. Thus, it should not form part of the *Job Task / Activity* span; rather, it should be separately annotated using the *Industry/Workplace* category (see section 3.1)

Except for a 45-minute to 1 hour period of time in the morning when the workers are **[loading the trucks ~~within the partially enclosed truck bays~~]**, …

- A similar example is below, in which *"ventilated blasting shed"* should be separately annotated as an *Industry/Workplace* annotation.

Samples 4–7 and 15–22 were obtained during **[sandblasting of prestressed concrete building structures ~~inside a ventilated blasting shed~~]**

- The phrase *"steel structure"* may be considered as an *Industry/Workplace* (see section 3.1) in certain contexts. However, in the example below, it corresponds to the object that is affected by the blasting activity, and so should be included within the *Job Task/Activity* span. The phrase *"under a bridge"* is excluded because this is a separate phrase describing *where* the blasting was taking place rather than what was being blasted. As such, the word "bridge" should be separately annotated using the *Industry/Workplace* category.

Samples 8–14 were obtained during **[abrasive blasting of the steel structure ~~under a bridge~~]**

  - In the example below, *"outdoors"* is a phrase providing information about where the concrete wall was located (and hence, where the sandblasting took place). Similarly to the examples above, this means that "outdoors" should be separately annotated as an *Industry/Workplace* annotation.

Samples 1–3 were obtained during **[sandblasting of a concrete wall ~~outdoors~~]**

- Details about the **means** of carrying out a task should be excluded from the span, if they correspond to *tools or equipment* used to perform the activity.

For small jobs, a **[~~jackhammer may be used to~~ break up the heavy block of sawed concrete]**

Tables III and IV present data from a construction site where the **[concrete walls of a large building were being smoothed ~~using an electric angle grinder~~]**

3.4 Substance or Exposure Measured

3.4.1 Definition

The name of a substance, chemical or pollutant (a recognised exposure entity) that is measured or sampled.

3.4.2 Annotation scope

✓ Include:

- Any word or phrase (including abbreviations) indicating the name, identity or chemical composition of the substance, pollutant or chemical (a recognised exposure entity) that is sampled.

> **[PAHs]** were measured by using gas chromatography mass spectrometry.

> **[Elemental carbon]** (**[EC]**), **[nitrogen dioxide]** and **[inhalable dust]** were measured using sampling equipment attached to the subjects

- Mentions of diesel (when used to describe emissions/exposures)

> Spot samples of **[diesel emissions]** contaminants were taken in areas where the miners worked

> [Elemental carbon] was analyzed as a marker of **[diesel exhaust]**.

> The purpose of this study was to characterize [respirable dust], [crystalline silica], and **[diesel]** exposures to construction workers on a large highway construction project in Boston, Massachusetts

- Mentions of particles and dust when mentioned in the context of exposures.

> The results suggest that in-cab **[particle]** exposures are positively related to smoking, ambient **[particle]** concentrations, truck age, and open windows, with other significant modifying factors such as weather.

> The aim of the present study was to investigate if underground miners exposed to **[dust]** and [diesel exhaust] in an iron ore mine would show signs of airway inflammation as reflected in induced sputum.

> Since [crystalline silica] in the form of [quartz] is a major component of concrete, airborne **[respirable quartz dust]** may be produced during construction work involving the disturbance of concrete, thereby producing a silicosis hazard for exposed workers.

- All substances measured, even if these were found to be non-detectable / quantifiable

> Exposure to **[silica], [radon]** and **[arsenic]** was minimal or absent in all these mines.

✗ Exclude:

- Any word or phrase describing specific metrics of the substance measured including peak concentrations or average concentrations.

> On a calm day one could expect a higher particle concentration at the paving site, especially since **~~[peak concentrations]~~** up to 2.2 × 105 cm−3 were frequently observed during paving activities.

- Words or phrases that describe general classes of substances.

> The personal samplers for the four **~~[gaseous components]~~** were generally not suitable.

> Characterization of Occupational Exposure to **~~[Air Contaminants]~~** in Modern Tunnelling Operations

- Phrases that mention ONLY the state/form and NOT the type of substance or exposure

> In total 142 **~~[inhalable samples]~~** and 144 **~~[respirable samples]~~** were collected

> [Respirable dust] samples were combined prior to analysis of **~~[crystalline constituents]~~** if the amount of dust in each sample was not sufficient for analysis (<0.7 mg).

- Mentions of materials that DO NOT constitute specific exposure entities.

> The mill is set to remove a predetermined depth of **~~[asphalt]~~** or **~~[concrete]~~**.

  - Note that <u>*constituents*</u> of these materials may constitute valid exposure entities and should be annotated.

> Three bulk samples of the **[<u>*concrete*</u> dust]** were found to be 38, 47, and 43 percent [silica].

- Mentions of substances whose context denotes that they do not relate directly to exposures

> In all projects the machinery was **~~[diesel]~~** powered and the same types of machines were used.

> Workers performing the shotcrete technique and TBM excavation method occasionally wore **[~~dust~~]** masks

The ~~**[exhaust]**~~ backpressure is determined by summing the pressure drops across the water- bath scrubber, ~~**[exhaust]**~~ piping, and the filter

- Words and phrases that relate to exposures other than those concerning particulate or gaseous substances (e.g., noise, temperature, humidity, etc.)

The average ~~**[noise]**~~ levels were consistently high among the various trades, ranging from a mean of 86 dBA for carpenters to a mean of 91 dBA for operating engineers.

[CO2], ~~**[temperature]**~~, and **[**~~**relative humidity**~~**]** are indicators of room occupancy and ventilation

3.4.3 Annotation span

✓ Include:

- The fraction or state/form of the relevant substance if this is specified.
  - This information may be specified as part of the same noun phrase containing the substance name, as in the following examples:

A selection of 61 filters was sent to an external laboratory to determine the content of **[*respirable* crystalline silica]** in the collected **[*respirable* dust]**.

Filters and tubes for collection of **[*particulate* PAHs]** and **[*gaseous* PAHs]** were mounted in series during sampling.

This paper presents the **[diesel exhaust *aerosol*]** (**[DEA]**) concentration data collected in these mines

Operations such as longwall moves and stonedusting, which generate elevated levels of dust, cause severe interference to **[*submicron* DP]** measurements

The number of [particles] does not necessarily correlate with the mass of [particles], since **[*ultrafine* particles]** often are numerous, but does not contribute much to the [particle] mass

The pollutants monitored include **[particulate matter *less than or equal to 10 µm diameter*]** (**[PM10]**), and [**PM2.5**], depending on the site.

Tables III and IV summarize the overall descriptive statistics for **[*submicrometer-sized* elemental carbon].**

The accuracy of this method for measuring **[*total* carbon]** was ascertained in this measurement campaign by analysing a known quantity of carbon in the form of sucrose.

- The information about fraction or state/form may also be specified in a neighbouring prepositional phrase.

Silicosis is a debilitating and sometimes fatal lung disease resulting from breathing [***microscopic particles*** **of crystalline silica**].

The measurements showed considerable difference in exposure levels between various operations, in particular for the [***inhalable fraction*** **of the dust].**

- Words such as "matter", if they occur within the same phrase as the substance.

The diffusion charger measures the total active surface area of **[particulate *matter*]**

- Words such as "fraction", if they occur within the same phrase as the description of a specific fraction.

It was shown in the laboratory, and in underground mines, that inertial impaction, followed by gravi-metric analysis, can be used to separate and sample [DEA] and **[mineral dust aerosol *fractions*]**

✗ Exclude:

- Words corresponding to specific metrics or measurements of the substance, including peak concentrations or average concentrations.

Significant Pearson correlation coefficients were found between daily **[elemental carbon], [PM fractions]** and **[particle ~~number concentrations~~].**

The **[particle ~~mass~~]** was measured gravimetrically

Cleaning of poultry houses also showed the highest **[inhalable endotoxin ~~levels~~]**

The **[~~percentage of~~ crystalline silica]** was overall higher in the **[respirable dust ~~samples~~]** than the **[inhalable dust ~~samples~~].**

To measure the **[endotoxin ~~concentration~~]** on the filters, dust was extracted individually in 10 ml of sterile, non pyrogenic water.

Concrete was being mechanically disturbed in order to obtain data concerning **[respirable crystalline silica dust ~~exposures~~]**.

3.5 Occupational Hygiene (OH) Measurement Device

3.5.1 Definition

The device, tool, apparatus, or sampling head used to measure levels of particulate and gaseous exposures by occupational hygienists in the workplace.

3.5.2 Annotation scope

✓ Include:

- Any word or phrase that names or describes the sampling head, apparatus or device used to sample or monitor particulates. This includes:
    - Different types of sampling heads

Total dust was collected on glass fibre filters fitted in **[IOM samplers]**

Personal inhalation exposure measurements were obtained using **[Higgins Dewell cyclones]** in accordance with MDHS 14/4 (HSE, 2014)

Large-volume integrative samples for detailed chemical analysis were collected using a **[47-mm TissueQuartz filter sampler]** with a **[large cyclone separator]** (less than 2.5-µm- diameter cutoff) operated at 16.7 L/min for 8 to 12 hours

-
    - References to cassettes or other types of filter holders used within sampling heads

Total dust and particulate polycyclic aromatic hydrocarbons (PAHs) were collected on acrylic copolymer membrane filters (Versapore 800, Gelman Sciences, Ann Arbor, Mich.), with a 0.8 m pore size, fitted in **[25 mm closed-faced aerosol filter cassettes]** (Gelman Sciences) at a sampling flow rate of 2 L/min.

The elemental and organic carbon samples (for thermal-optical analyses) were obtained by drawing air at approximately 4 Lpm through 47-mm Pallflex QAOT quartz fiber filters and a stainless steel support screen, both of which were held in **[aluminum 47-mm in-line filter holders]**.

A number of difficulties were documented when sampling for diesel exhaust using organic carbon, e.g., a consistent positive interference (+26%) when **[open-faced monitors]** were sampled side-by-side with [cyclones].

- Mentions of containers used to collect samples of gases.

A range of **[Dräger stain tubes]** were used to identify and semi-quantify the airborne contaminants; benzene, sulphur dioxide, and nitrogen dioxide

VOC samples were collected in [**SKC Silcosteel-treated interior 15 L canisters]** that had been put under vacuum and controlled with a 10 Hr restrictor regulator

[Gas detector tubes] for CO, CO2, SO2, and NO2 were collected on a time-available basis

- Devices used to collect real-time samples.

Future sampling efforts will include more GPS tracking units and **[real-time particle exposure monitors]** in the vehicle samples

The carbon monoxide and nitrogen dioxide concentrations were measured with **[direct-reading electrochemical sensors]** with a data-logging facility built into the instrument

A **[MicroAeth AE51 aethalometer]** equipped with a [microCyclone] (time base 60 s, flow rate 50 ml min−1) measured BC concentrations in a fixed location

- Words or phrases referring to passive samplers or "badges".

NO2 was measured using a **[passive sampler]** and respirable particulate was determined gravimetrically

- References to equipment or apparatus described as being "personal", as long as they provide information that can identify the sampler type used in the measurement series.

Nitrogen dioxide was measured using **[personal *diffusive* samplers]** and ion chromatographic analysis

Sample collection for respirable dust, crystalline silica, and diesel particulates measured as elemental carbon was done using [cyclones] and **[personal *cascade impactors*]**.

Sample collection was performed using either a [BGI-4 Higgins & Dewell] (Waltham, Massachusetts), an [MSA 10 mm nylon Dorr-Oliver] (Pittsburgh, Pennsylvania), or an **[SKC Aluminum personal *cyclone*]** at a sampling flow rate of 2.2, 1.7, or 1.9 liters per minute (lpm), respectively

- Phrases that correspond to names and/or models of devices as well as phrases that describe/characterise devices.

The **[*P-Trak*]** is a **[condensation particle counter]** that monitored the number of particles every second during 8 hr.

Air concentrations of NO2 and NH3 were measured with **[portable direct-reading electrochemical sensors]** with data logging built into the instruments (**[*PAC7000*],** Dräger AK, Lübeck, Germany)

Concurrently with the **[DustTrak monitor]**, a ***[Q-Trak]*** (TSI) was used to obtain real-time data on carbon monoxide (CO), carbon dioxide (CO2), temperature, and relative humidity

  - Note that descriptions and model names should be separately annotated, unless they occur as part of the same noun phrase.
    - In the example below, the phrase *"cyclone separator"* constitutes a description of the sampler, while *"Casella T13026/2"* constitutes the model name of the sampler. The model name is provided in brackets. Hence, it does not form part of the same noun phrases as the sampler description, and so *"cyclone separator"* and *"Casella T13026/2"* should constitute two separate *OH Measurement Device* annotations.

Respirable dust was collected on 37-mm cellulose acetate filters with a pore size of 0.8 m using a **[cyclone separator]** (**[*Casella T13026/2*]**, London, UK) at a sampling flow rate of 2.2 L/min

✗ Exclude:

- Any word or phrase describing parts of the so called "sampling train" apparatus including the type of sampling pumps and the tubing connecting them to the sampling apparatus.
    - Note the difference here between tubes used to connect pumps to sampling heads (which are NOT to be annotated) and tubes used to collect gaseous samples (which SHOULD be annotated)

> The samplers were strapped on duplicate (one at each side) at the upper part of the chest of the farmers, and a ~~**[silicone rubber tube]**~~ connected each sampler to a pre-calibrated at an operational flow of 3.5 l min ~~**[AirChek XR5000 portable pump]**~~ (SKC Inc., Eighty Four, PA, USA).

> This unit consisted of a ~~**[NIOSH-approved pump]**~~, a [nylon cyclone], and a [cassette] containing a polyvinyl filter

- Mentions of the materials (e.g., **<u>filters</u>**) on which samples are collected

> Total dust and particulate polycyclic aromatic hydrocarbons (PAHs) were collected on ~~**[acrylic copolymer membrane filters]**~~ (Versapore 800, Gelman Sciences, Ann Arbor, Mich.), with a 0.8 m pore size.

> The upper stages were collected on ~~**[mylar substrate]**~~ sprayed with 316 Silicone Release Spray (Dow Corning Corporation, Midland, Michigan) to prevent particle bounce.

> The aldehydes were collected on a ~~**[hydroxymethyl piperazine (DNPH) matrix]**~~ housed in [glass sorbent tubes] (manufactured by Supelco, Inc.).

- Any word or phrase describing devices or analytical equipment used for the laboratory analysis or laboratory determination of the substance.

> The particle mass was measured by ~~**[microbalance (model AT261; Mettler Toledo, Inc., Columbus, Ohio)]**~~, with a detection limit of 0.031 mg/m3 based on 8 hr of sampling.

> Oil mist was measured by using a ~~**[Fourier transform infrared (FTIR) spectrophotometer (PE 1600 FTIR; PerkinElmer, Waltham, Mass.)]**~~; oil vapor was measured by ~~**[chromatography (CG) with a flame ionization detector (FID)]**~~.

EC and OC were determined by Sunset Laboratory Inc. (Tigard, OR, USA) using an **[~~OCEC Dual- Optical Analyzer~~]** according to NIOSH Method 5040 (NIOSH, 2003).

All [DPM Cassettes] were analysed for total carbon (TC), organic (OC) and elemental carbon (EC) via the NIOSH Method 5040 by Coal Services Pty Ltd, using a **[~~Sunset Laboratories thermo-optical instrument~~]**.

- Any word or phrase that corresponds to a **<u>technique or method</u>**, rather than a physical device or piece of apparatus.

The samples were analysed for endotoxin using the Limulus Amebocyte Lysate assay and crystalline silica content using **~~[X-ray diffraction].~~**

- Names of companies/manufacturers that are not included in the same phrase as names/descriptions of devices or apparatus

These measurements were performed, under contract, by **~~[Scott Environmental Technology, Inc]~~**

This was an [IOM inhalable dust sampler] (**~~[SKC West]~~**, Fullerton, CA), containing a 25 mm diameter polyvinylchloride (PVC) filter (pore size 5 µm).

- Mentions of devices or apparatus that are used to measure exposures other than those concerning particles or gases (e.g., temperature or humidity)

The particle collectors, their pumps, and a **[~~real-time monitor for temperature and humidity~~]** (**[~~HOBO~~]**; Onset Computer Corp, Bourne, Mass.) were mounted in a box housing connected to an external battery

The outdoor temperature was measured locally using a portable **~~[OBH Nordica weather station]~~** with a measurement accuracy of ±1°C

- Vague phrases that do not provide any useful clues about the type/nature of the devices or apparatus.
    - This includes phrases corresponding to devices or apparatus which:
        - Only mention the type of substance collected.

- If any substances are mentioned within these vague phrases, they should be separately annotated using the *Substance Measured* category (see Section 3.3).

▪ Only mention that the device is personal in nature.

The ~~**[dust sampling equipment]**~~ could be used according to well-established standard operation procedures

The ~~**[EC samplers]**~~ were placed near the breathing zone of the parking ramp attendants and close to the open window

Two types of ~~**[personal diesel exhaust aerosol samplers]**~~ ([~~**PDEAS]**~~) were developed to achieve this result.

Ten farms were randomly selected in Yolo and Solano counties and workers were invited to wear ~~**[personal sampling equipment]**~~ to measure inhalable and respirable dust levels during various operations.

3.5.3 Annotation span

✓ Include:

- Information that helps to fully characterise the device/equipment, <u>if this included in the same noun phrase</u>. Such information may include (among others):
  - The name of the manufacturer and/or model name

This was also the case in asphalt paving, confirmed by the results of the **[*<u>Grimm</u>* PDM]**, which showed that the concentration of particles above 700 nm was about 0.1 cm−3.

A range of **[*<u>Dräger</u>* stain tubes]** were used to identify and semi-quantify the airborne contaminants; benzene, sulphur dioxide, and nitrogen dioxide

The four gases: carbon monoxide (CO); oxygen (O2); lower flammability limit (LFL); and hydrogen sulfide (H2S) were measured using a **[*<u>Rae Systems MultiRae Plus</u>* four gas detector]**.

- Information about the size of the sampling head, cassette or filter holder

The first stage is an inertial pre- classifier, a **[*10-mm* Dorr-Oliver cyclone]** that separates and removes the larger, nonrespirable aerosol

- Number of parts in the sampling head, cassette or filter holder

An extra ring was inserted into the standard **[*three-part* 37-mm aerosol filter cassette]** (Millipore, MA, USA).

The second stage is a **[*four-nozzle* impactor].**

- Specifications of whether cassettes/monitors are open or closed.

Total dust and particulate polycyclic aromatic hydrocarbons (PAHs) were collected on acrylic copolymer membrane filters (Versapore 800, Gelman Sciences, Ann Arbor, Mich.), with a 0.8 m pore size, fitted in **[25 mm *closed-faced* aerosol filter cassettes]** .

There was a consistent positive interference (+26%) when **[*open-faced* monitors]** were sampled side-by-side with [cyclones].

- Material used to manufacture the sampling head, cassette or filter holder.

The elemental and organic carbon samples (for thermal optical analyses) were obtained by drawing air at approximately 4 Lpm through 47-mm Pallflex QAOT quartz fiber filters and a stainless steel support screen, both of which were held in **[*aluminum* 47-mm in-line filter holders]**

- Prepositional phrases that follow relevant devices or apparatus introduced in a noun phrase, if they help to better specify the nature if the device/apparatus

Usually, an [impactor] or a similar **[preseparator *with a submicron cutoff*]** is necessary to separate out the larger particles

- Annotate abbreviations separately from full forms.

Particle size distribution was measured by using a **[Scanning Mobility Particle Sizer]** (**[*SMPS*], [**model 3071A]; TSI GmbH, Aachen, Germany)

✗ Exclude:

- Manufacturer names that do not occur as part of the same noun phrase that names or describes the device or apparatus (e.g., if they are specified if brackets after the name or description of the device).

Concurrently with the [DustTrak monitor], a [Q-Trak] (~~[TSI]~~) was used to obtain real-time data on carbon monoxide (CO), carbon dioxide (CO2), temperature, and relative humidity

The carbon monoxide and nitrogen dioxide concentrations were measured with [direct-reading electrochemical sensors] with a data-logging facility built into the instrument (type [PAC III]; **~~[Drager Aktiengesellschaft]~~**)

- Places of manufacture

These measurements were obtained using [Sensidyne 5LC colorimetric detector tubes] (Sensidyne, Inc., **~~[Clearwater, FL]~~**).

3.6 Sample Type: Personal

3.6.1 Definition

Phrases denoting that collected samples of airborne substances, chemicals or pollutants represent **personal** exposures. **Personal samples** pertain to those measurements involving measuring concentrations of air contaminants directly in the breathing zone of the workers.

3.6.2 Annotation scope

✓ Include:

- Mentions that **<u>personal</u>** sampling/measurement techniques were carried out as part of methodological descriptions of the study.

**[Personal sampling]** was performed using GSP samplers.

**[Personal measurements]** of dust were carried out at both terminals.

[**Measurements of personal exposure]** to respirable dust and diesel exhaust were made

- Mentions of **<u>personal</u>** samples/measurements that are reported as results

Neither respirable quartz nor respirable dust were detected by the 127-minute **[personal breathing zone sample].**

- Phrases denoting that sampling was carried out on or near the workers' bodies

Sampling was performed in the **[breathing zone]** of the workers.

✗ Exclude:

- Any word or phrase describing *equipment or apparatus* used for sampling, including equipment described as being "personal".
  - Certain phrases describing equipment or apparatus used for measuring exposures should be annotated using the *Occupational Hygiene (OH) Measurement Device* category (see section 3.5).
    - This is the case, for "IOM personal samplers" in the example above, since "IOM" denotes the type of sampling head used.

Sampling was performed by **~~[IOM personal samplers]~~** worn by every worker during their shift.

Ten farms were randomly selected in Yolo and Solano counties and workers were invited to wear **~~[personal sampling equipment]~~** to measure inhalable and respirable dust levels during various operations.

- Note that phrases such as “personal sampling” should also be EXCLUDED from annotation with this category when they occur within descriptions of equipment, such as in the example above.

- Verbs describing the activity performed.

Air was **~~[sampled]~~** at mouth level.

- Any word or phrase referring to stationary/areal samples. Areal samples involving the measurement or estimation of the concentration of the substance concerned in the air of the workplace where the workers of interest are situated. This type of measurement can also be referred to as static or stationary.

**~~[Area]~~** measurements of PM mass concentrations and particle number concentrations were carried out at both terminals.

The IOM samplers were positioned at a **~~[table located in the centre of the room]~~**.

Air was sampled at **~~[mouth level]~~** of the involved workers.

- Any other phrase describing samples or measurements that are **not specifically specified to be personal ones.**

In addition, **~~[high-volume respirable dust samples]~~** were obtained on 37-mm PVC filters

A large percentage of the **~~[respirable measurements]~~** were below the limit of detection

3.6.3 Annotation span

✓ Include:

- In the case that a noun phrase denotes personal measurements, sampling or exposures, annotate the whole phrase.

In total, 79 **[personal *full-shift* samples]** of NO2 using direct-reading instruments (DRI) were evaluated.

4. Quick reference guides

4.1 Overview

This section contains abbreviated summaries of the guidelines introduced in the previous section. For both the general guidelines and each of the six different entity categories, guidelines are summarised in form of one-page tables. These include short descriptions of the inclusion and exclusion criteria for annotation scope and span; each criterion is supported by one of more supporting examples.

The tables are designed to complement the full guidelines, rather than acting as a substitute for them. That is to say, it is expected that the full set of guidelines will have been read and fully understood prior to embarking upon annotation; the tables in this section can then act as a mnemonic that can be consulted more easily and quickly consulted while performing the actual annotation task.

In contrast to the examples included in the previous section, most of the examples in the tables below consist only of single phrases, rather than sentences. This is partly for reasons of space, i.e., to ensure that examples for all inclusion and exclusion criteria can be included on the single page summaries. It should also hopefully help the examples to be more rapidly consulted and understood. However, in cases where context is required to illustrate a particular criterion, then this is provided.

The inclusion and exclusion criteria for annotation scope and annotation span are arranged in separate columns of the tables, and are colour-coded using green for positive (inclusion) examples and red for negative (exclusion) examples. Each criterion is numbered; supporting example(s) for each criterion are provided in the final column, indicated with the same number assigned to the corresponding criterion. The conventions used within these examples are as follows:

- Words to be included in annotated spans are highlighted in ***green italicised bold font***
- Words to be excluded from annotated spans are highlighted in ***red italicised bold font***
- Underlining in either ***red*** or ***green*** may be used to emphasise that certain words should excluded from or included in annotated spans, according to the criterion being illustrated
- In examples that include contextual information are ***"quoted, emboldened and italicised".*** Any words that do not form part of an exemplified annotation are shown in ***black***

4.2 General Guidelines

**Annotation Scope**		**Annotation Span**		**Examples**
**Include**	**Exclude**	**Include**	**Exclude**	
**1.** Annotate EVERY mention of each relevant entity in a document<br>**2.** Annotate relevant abbreviations and acronyms **in addition to, and separately from, full forms**<br>**3.** Consider **complete phrases** for annotation prior to considering parts of them<br>**4.** If the complete phrase does not correspond to an entity, but a part of it does, then this part may be annotated	**5.** “Embedded” entities (i.e., shorter annotations that occur within longer ones)	**6.** For entities that are nouns, the annotation will usually include all nouns and adjectives within the noun phrase<br>**7.** For entities including verbs, following prepositions or adverbs should be included if the action/activity can only be fully understood with the preposition or adverb<br>**8.** Each entity in a list should be annotated separately<br>**9.** General entities and more specific instances should be annotated separately<br>**10.** Entities on either side of a slash should be annotated **separately**	**11.** Determiners (e.g., *a, the, some*) and numerical values occurring at the start of noun phrases<br>**12.** The word “to” when annotating verbs in their infinitive forms<br>**13.** Subjects of verbs	**1.** ***“The EC samplers were placed near the breathing zone of the parking ramp attendants and close to the open window. There were several parking ramp attendants that worked in the booth throughout the day”***<br>**2.** **“total carbon (TC)”**<br>**3.** ***underground construction workers***<br>**4.** ***“underground construction project”***<br>**5.** ***underground construction workers; repair of an interstate highway***<br>**6.** ***asphalt strippers; lead locomotives***<br>**7.** ***“They send a P&D driver to pick the freight up”; “For larger jobs a clamshell-type device may be used with a backhoe to lift the block out”***<br>**8.** ***“paver operators, screedmen, roller drivers, and drivers of the binding agent trucks”***<br>**9.** ***“Amines (dietanolamine , diethylenetriamine, triehytlenete-tramine) were collected”***<br>**10.** ***“EC/OC/TC”***<br>**11.** ***The asphalt strippers; 22 miners***<br>**12.** ***To weld***<br>**13.** ***long-haul drivers drive***

4.3 Industry / Workplace

Definition	Annotation Scope		Annotation Span		Examples
	**Include**	**Exclude**	**Include**	**Exclude**	
Either:<br>• The industry involved in the sampling series<br>• The type of workplace or environment involved in the sampling series	**1.** Economic activities, industries or sectors<br>**2.** General areas, plants or factories<br>**3.** Indoor areas where work takes place<br>**4.** Specific outdoor areas<br>**5.** Vehicles and other types of mobile heavy equipment<br>**6.** Stationary heavy equipment used to specify worker locations<br>**7.** Specific features of the work environment, which may impact on the types and/or levels of exposures (underground, enclosed, outdoors, etc);<br>**8.** Simulated work places	**9.** Specific tasks performed by workers on a daily basis<br>➢ **Use *Job task / Activity* instead**<br>**10.** Phrases that form part of job task descriptions, even if these phrases could also correspond to workplaces<br>➢ **Use *Job task / Activity* to annotate the whole phrase**<br>**11.** Mentions of light equipment not providing information about location of workers<br>**12.** Names of locations<br>**13.** Named sites or areas<br>**14.** General phrases not providing information about the nature of the working environment and/or industry<br>**15.** Words/phrases that occur as part of company names, even if they could otherwise denote workplaces<br>**16.** Very general features of the environment, rather than specific information about the nature of the workplace<br>**17.** Specific job roles<br>➢ **Use *Occupation /Job Title* instead**<br>**18.** Parts of phrases that characterise workers (even if the characterisation is based on an industry or workplace).<br>➢ **Use *Occupation /Job Title* to annotate the whole phrase**	**19.** Words in the same phrase that help to describe the workplace more precisely<br>**20.** Words like *industry* or *services*, if they occur in the same phrase that mentions an industry or economic activity<br>**21.** Words like *site* or *environment* if they help to fully characterise a place of work	**22.** References to named places<br>**23.** Words that do not constitute part of the industry name or description	**1.** ***underground tunnel construction; railway transport; mechanical services***<br>**2.** ***bitumen plant; diesel factory; dock area***<br>**3.** ***diesel-use mines; tunnels; repair and maintenance workshop***<br>**4.** ***four-lane motorway; heavy repair area***<br>**5.** ***diesel powered fork-lift trucks; lead locomotives; water truck; asphalt mill***<br>**6.** ***pneumatic drills; conveyor belt***<br>**7.** ***underground locations; surface; enclosed cabin; outdoors***<br>**8.** ***test tunnel; simulated section of mine roadway***<br><br>**9.** ***welding***<br>**10.** ***reconstruction of an interstate highway bridge.***<br>**11.** ***electric angle grinder; jackhammer***<br>**12.** ***Dalston***<br>**13.** ***Site A; Area 1***<br>**14.** ***area; work site; plant; machine***<br>**15.** ***"Ontario Southland Railways"; "Tower Colliery"***<br>**16.** ***city; suburbs***<br>**17.** ***asphalt workers***<br>**18.** ***"highway construction workers"; "mine workers"; "workers on the farms"; "operators in the refinery"***<br><br>**19.** ***underground diesel- use mine; diesel-powered loaders***<br>**20.** ***Engineering services***<br>**21.** ***Construction sites***<br><br>**22.** ***Mine M; London Tunelling Environment***<br>**23.** ***bus transport companies***

4.4 Occupation / Job Title

Definition	Annotation Scope		Annotation Span		Examples
	Include	Exclude	Include	Exclude	
A phrase that characterises a person or group of people in terms of their occupation, job title, position, the general type of work that they typically undertake, the types of equipment with which they typically work or the type of location in which they typically work.	Phrases that characterise people according to the type of work that they undertake. This includes (but is not limited to):<br><br>1. Their specific job title<br>2. The nature of their work<br>3. The materials with which they work<br>4. The general location or environment in which they work<br>5. The equipment or vehicles that they generally operate<br>6. The tasks that they undertake as part of their work<br>7. The generic activity (industry) in which they work	8. Descriptions of the nature, character, location or industry of work that do not refer to specific people<br>• **Use *Industry/ Workplace* or *Job task / Activity* (as appropriate) instead**<br>9. Characterisations of workers that do not concern the nature of their work, e.g. what type of shifts they work<br>10. General phrases that do not correspond to a specific occupation or type of job<br>11. Words/phrases that do not refer to worker in the context in which they occur , even if the same word/phrase may refer to workers in other contexts<br>12. Mentions of workers who do not constitute subjects of exposure measurements (i.e., those who are not being sampled).	13. All nouns and adjectives that occur within simple noun phrases that characterise workers<br><br>Modified noun phrases consisting of:<br><br>• A noun corresponding to worker(s)<br>• A subsequent *modifying* phrase, which better characterises the worker(s). One of the following:<br>14. Prepositional phrase<br>15. Phrase beginning with verb in "ing" form<br>16. Relative clause starting with "who"<br>17. Reduced relative clause, i.e., shortened relative clause in which the word "who" is omitted	18. Information following noun phrases, corresponding to locations or conditions under which workers' activities are taking place, if the noun phrase itself sufficiently characterises a job or occupation<br>19. Named places<br>20. Personal characteristics of workers (e.g., gender/ethnicity)<br>21. Job codes in occupational schemes	*1.* ***electrician; carpenter***<br>*2.* ***asphalt strippers; gate controllers***<br>*3.* ***concrete workers; battery workers***<br>*4.* ***mine workers; railway workers surface workers; workers in toll stations***<br>*5.* ***heavy equipment operator; concrete cutter operator; worker using the grinder***<br>*6.* ***drill and blast crew; shaft-drilling crew***<br>*7.* ***underground construction workers; tunnel construction workers***<br><br>*8.* ***asphalt work; drilling***<br>*9.* ***day shift workers; workers on the afternoon and night shifts***<br>*10.* ***workers; miscellaneous trades***<br>*11.* ***"A metal shroud was attached to the grinder"***<br>*12.* ***industrial hygienist***<br><br>*13.* ***shaft drillers; underground coal miners***<br>*14.* ***drivers of trains; workers on the farms***<br>*15.* ***workers operating an asphalt mill; worker using the grinder***<br>*16.* ***Workers who are employed in the production of SiC***<br>17. **Workers involved in the production of SiC**<br><br>*18.* ***Paver operators seated on paving machines without a cabin***<br>*19.* ***London Transport employees***<br>*20.* ***white male underground coal miners***<br>*21.* ***railway workers (ISIC 71)***

4.5 Job Task / Activity

**Definition**	**Annotation Scope**		**Annotation Span**		**Examples**
	**Include**	**Exclude**	**Include**	**Exclude**	
Specific and well-defined physical activities or actions carried out by workers as part of their daily duties<br><br>Annotations should capture as much information as is provided about the activity conveyed by:<br>• The word (noun/verb) corresponding to the basic activity<br>• Other words/phrases occuring in the immediate context of this word, which specify/clarify the activity more precisely (if present)<br><br>**Annotations should only be created if the annotated span in isolation allows a specific and well-defined activity to be understood.**	1. Single words (verbs or nouns) ONLY if they correspond to specific and well-defined job activities that can be understood in isolation, and as long as no further neighbouring details about the nature of the activity are provided in the text<br>2. Longer phrases should be annotated whenever they are present in the text and as long as they provide further details about what the activity involves	3. Phrases that describe **where** the task/activity was carried out, as long as DO NOT correspond to the object affected by the activity<br>➢ **Use *Industry/ Workplace* instead**<br>4. Parts of phrases that characterise workers in terms of tasks undertaken<br>➢ **Use *Occupation / Job Title* to annotate whole phrase**<br>5. Phrases starting with a verb denoting locations of work<br>➢ **Use *Industry / Workplace* to annotate locations**<br>6. Vague or general action verbs or nouns, without neighbouring words providing further task-specific information<br>7. Unintended actions/ accidents, or activities not forming part of a worker's daily duties<br>8. Phrases **characterising people** in terms of the job that they do, even if in terms of the tasks that they undertake<br>➢ **Use *Occupation / Job Title* instead**<br>9. **General** economic activities or industries<br>➢ **Use *Industry / Workplace* instead**	The word/phrase introducing the basic activity, along with any immediately following phrases providing information about what the activity involves. Typically:<br>• Nature/characteristics of the activity<br>• Materials, items or objects affected by it (even if they are workplaces).<br>• Initial and/or resultant state, e.g.,<br>○ Starting or finishing position<br>○ Objects resulting from or constituting the outcome of the activity<br><br>Specifically:<br><br>For activities described using verbs:<br>10. Direct objects of verbs<br>11. Prepositional phrases following verbs<br>12. Prepositional phrases following direct objects of verbs<br>13. Activities described using the passive voice<br><br>For activities described using nouns:<br>14. Other nouns/adjectives in the same noun phrase<br>15. Prepositional phrases immediately following noun phrases<br>16. Subsequent prepositional phrases<br><br>17. Sufficient text should be annotated to adequately describe a task<br>18. Distinct activities that are temporally ordered should be separately annotated<br>19. If a "higher level"task is broken down into one or more specific tasks, then all tasks should be separately annotated	20. Descriptions of **where the activity was carried out** or **where items involved in the task were located**<br>➢ **Use *Industry / Workplace* instead**<br><br>21. Details about the means of carrying out the activity, if this corresponds to tools or equipment used to carry out the activity	***1.*** *welding; sawing*<br>***2.*** *concrete pouring; mechanical mowing of weeds; feeding poultry*<br><br>***3.*** **"Measurements were carried out on one bitumen plant"**<br>***4.*** *drill and blast crew; shaft drilling crew*<br>***5.*** *working indoors in office environments;*<br>*use forklift trucks*<br>***6.*** *production various operations; construction activities*<br>***7.*** *completed an activity questionnaire*<br>***8.*** *asphalt workers*<br>***9.*** *transportation; mechanical services*<br><br>***10.*** *chipping concrete; drilling concrete highway pavement*<br>***11.*** *tunnelling through areas of clay*<br>***12.*** *drill holes through the block; lift the concrete block out of the pavement*<br>***13.*** *locomotives are repaired; expansion joints were being sawed in the fresh concrete; restroom floors could be installed*<br>***14.*** *concrete mixing; pneumatic drilling*<br>***15.*** *repair of an interstate highway*<br>***16.*** *milling of asphalt from concrete highway pavement*<br>***17.*** *concrete finish work*<br>***18.*** *"concrete drilling work during interstate highway repair"*<br>***19.*** ***"Rock support includes** sealing of the rock **by** spraying wet concrete onto the excavated surface"*<br><br>***20.*** *blasting of steel structures under a bridge; inspecting the contents of containers found on containers trucks coming through the gate of the port terminals*<br>***21.*** *jackhammer may be used to break up the heavy block of sawed concrete*

4.6 Substance or Exposure Measured

<table>
<tr><th rowspan="2">Definition</th><th colspan="2">Annotation Scope</th><th colspan="2">Annotation Span</th><th rowspan="2">Examples</th></tr>
<tr><th>Include</th><th>Exclude</th><th>Include</th><th>Exclude</th></tr>
<tr>
<td>Name of a substance, chemical or pollutant (a recognised exposure entity) that is measured or sampled.</td>
<td>1. Any word or phrase (including abbreviations) indicating the name, identity or chemical composition of the substance, pollutant or chemical (recognised exposure entity) sampled
2. Mentions of diesel (when used to describe emissions/exposures)
3. Mentions of particles and dust when mentioned in the context of exposures
4. All substances measured, even if results were found to be non-detectable / quantifiable.</td>
<td>5. Specific metrics of the substance measured, including peak concentrations and average concentrations.
6. General classes of substances
7. Phrases that mention only the state/form and NOT the type of substance or exposure
8. Mentions of materials that do not constitute specific exposure entities
9. Mentions of substances whose context denotes that they do not relate directly to exposures
10. Exposures that do not concern particulate or gaseous substances</td>
<td>11. Fraction or state/form of the relevant substance, if specified
• May occur in the same noun phrase as the substance name or in a neighbouring prepositional phrase
12. Words such as “matter” that occur within the same phrase as the substance
13. Words such as “fraction” that occur within the same phrase as the description of a specific fraction</td>
<td>14. Words corresponding to specific metrics or measurements of the substance measured, including peak concentrations and average concentrations</td>
<td>1. Particles; elemental carbon; EC; PAHs
2. diesel emissions; diesel exhaust
3. “In-cab particle exposures are positively related to smoking”; respirable quartz dust; concrete dust
4. “Exposure to silica, radon and arsenic was minimal or absent in all these mines”

5. peak concentrations
6. gaseous components; air contaminants
7. inhalable samples; crystalline constituents
8. “The mill is set to remove a predetermined depth of asphalt or concrete.”
9. “In all projects the machinery was diesel powered”; “Workers occasionally wore dust masks”
10. noise; temperature; relative humidity

11. <u>respirable</u> dust; <u>gaseous</u> PAHs; diesel exhaust <u>aerosol</u>; <u>submicrometer-sized</u> elemental carbon; particulate matter <u>less than or equal to 10 μm diameter</u>; <u>total</u> carbon; <u>microscopic particles</u> of crystalline silica; <u>inhalable fraction</u> of the dust
12. particulate <u>matter</u>
13. mineral dust aerosol <u>fractions</u>

14. particle <u>number concentrations;</u> particle <u>mass; inhalable endotoxin levels;</u> respirable dust <u>samples; percentage of</u> crystalline silica</td>
</tr>
</table>

4.7 Occupational Hygiene (OH) Measurement Device

Definition	Annotation Scope		Annotation Span		Examples
	**Include**	**Exclude**	**Include**	**Exclude**	
The device, tool, apparatus, or sampling head used to measure levels of particulate and gaseous exposures by occupational hygienists in the workplace.	1. Different types of sampling heads<br>2. Cassettes or other types of filter holders used within sampling heads<br>3. Containers used to collect samples of gases<br>4. Devices used to collect real-time samples<br>5. Passive samplers or "badges"<br>6. References to equipment or apparatus described as being "personal", as long as they provide information that can identify the sampler type used in the measurement series<br>7. Names or models of devices/apparatus as well as descriptions or characterisations of them	8. Parts of the so called "sampling train" apparatus including the type of sampling pumps, the tubes connecting them to the sampling apparatus and position.<br>• **NOTE: tubes used to collect gaseous samples should be annotated (criterion 3)**<br>9. Materials (e.g., filters) on which samples are collected<br>10. Devices or analytical equipment used for the laboratory analysis or determination of the substance.<br>11. Any word/phrase corresponding to a technique or method<br>12. Names of companies or manufacturers not occurring in the same phrase as names / descriptions of devices or apparatus<br>13. Apparatus used to measure exposures other than particles or gases (e.g., temperature)<br>14. Vague phrases not providing useful clues about the type/nature of the device. Includes:<br>• Phrases that only mention the type of substance collected<br>➢ **Any relevant substances should instead be annotated as *Substance Measured***<br>• Phrases that only mention that the device is personal in nature	15. Names of manufacturers or models in the same noun phrase as the device/apparatus<br>16. Size of device or apparatus in the same noun phrase<br>17. Number of parts in device or apparatus in the same noun phrase<br>18. Specifications of whether cassettes are open or closed in the same noun phrase<br>19. Material used to manufacture device or apparatus in the same noun phrase<br>20. Prepositional phrases that follow noun phrases, if they help to better specify the nature of the device/apparatus<br>21. Annotate abbreviations separately from full forms	22. Manufacturer names that do not occur in the same noun phrase that names or describes the device or apparatus (e.g., if they are specified if brackets after the name or description of the device)<br>23. Places of manufacture	***1.*** ***IOM samplers; Higgins Dewell cyclones***<br>***2.*** ***25 mm closed-faced aerosol filter cassettes; aluminum 47-mm in-line filter holders; open-faced monitors***<br>***3.*** ***Dräger stain tubes; Silcosteel-treated interior 15 L canisters***<br>***4.*** ***direct-reading electrochemical sensors***<br>***5.*** ***passive sampler***<br>***6.*** ***personal cascade impactors***<br>***7.*** ***P-Track; Casella T13026/2***<br>***8.*** ***silicone rubber tube; AirChek XR5000 portable pump***<br>***9.*** ***acrylic copolymer membrane filters; mylar substrate***<br>***10.*** ***Microbalance; OCEC Dual- Optical Analyzer***<br>***11.*** ***X-ray diffraction***<br>***12.*** ***"These measurements were performed by Scott Environmental Technology, Inc"; "This was an IOM inhalable dust sampler (SKC West, Fullerton, CA)"***<br>***13.*** ***real-time monitor for temperature and humidity; OBH Nordica weather station***<br>***14.*** ***dust sampling equipment; EC samplers; personal diesel exhaust aerosol samplers; personal sampling equipment***<br>***15.*** ***Grimm PDM; Dräger stain tubes***<br>***16.*** ***10-mm Dorr-Oliver cyclone***<br>***17.*** ***three-part 37-mm aerosol filter cassette; four-nozzle impactor***<br>***18.*** ***25 mm closed-faced aerosol filter cassettes; open-faced monitors***<br>***19.*** ***aluminum 47-mm in-line filter holders***<br>***20.*** ***preseparator with a submicron cutoff***<br>***21.*** ***Scanning Mobility Particle Sizer (SMPS)***<br>***22.*** "**a Q-Trak (TSI) was used**";<br>***23.*** ***"Sensidyne 5LC colorimetric detector tubes (Sensidyne, Inc., Clearwater, FL)"***

4.8 Sample Type: Personal

Definition	Annotation Scope		Annotation Span		Examples
	**Include**	**Exclude**	**Include**	**Exclude**	
Phrases denoting that collected samples of airborne substances, chemicals or pollutants represent **personal** exposures.<br><br>**Personal samples** pertain to those measurements involving measuring concentrations of air contaminants directly in the breathing zone of the workers	1. Mentions that **personal** sampling /measurement techniques were carried out as part of methodological descriptions of the study<br>2. Mentions of **personal** samples / measurements as part of the reporting of results<br>3. Phrases denoting that sampling was carried out on or near the workers' bodies	4. Any word or phrase describing *equipment or apparatus* used for sampling, including equipment described as being "personal".<br>➢ **Some such phrases should be annotated using OH Measurement Device instead**<br>• Note that phrases such as "personal sampling" should also be excluded when they occur within descriptions of equipment<br>5. Verbs describing the activity performed<br>6. Words/phrases describing the performance of static, stationary or areal samples.<br>7. Any other phrase describing samples or measurements that are not specifically specified to be personal ones	8. For noun phrases denoting personal measurements or sampling, annotate the whole phrase, even if some of the words if the phrase do not concern personal sampling		***1.*** ***"Personal sampling was performed using GSP samplers."***<br>***2.*** ***"Neither respirable quartz nor respirable dust were detected by the 127-minute personal breathing zone sample"***<br>***3.*** ***breathing zone***<br>***4.*** ***IOM personal samplers; personal sampling equipment***<br>***5.*** ***sampled***<br>***6.*** ***area measurements; "The IOM samplers were positioned at a table located in the centre of the room."***<br>***7.*** ***high-volume respirable dust samples; respirable measurements***<br>***8.*** ***personal full-shift samples***
