# Supplementary material for: Supporting the working life exposome: Annotating occupational exposure for enhanced literature search
Source: PLoS One. 2024 Aug 15;19(8):e0307844. doi: 10.1371/journal.pone.0307844 (PMC11326626; doi:10.1371/journal.pone.0307844)
Supplement: S2 Appendix — (PDF) [file pone.0307844.s002.pdf]

S2 Appendix: Rule-based combination of annotations

This supplementary document describes our rule-based approach to determine an optimal, unified set of annotations, given two sets of alternative annotations produced by different human annotators. Based an analysis of the individual annotation trends of each annotator, we develop sets of rules that define how to combine their annotations, taking into account the relative strengths, weaknesses and annotation behaviours of the different annotators.

We firstly describe and motivate the application of the rule-based algorithm and the format of the rules applied. We then show how the application of the rules can result in unified sets of annotations that exhibit more homogenous characteristics, compared to the application of a simpler "best annotator" approach, i.e., using only the annotations of whoever is perceived to be the better annotator of a pair.

Rule-based algorithm

The algorithm firstly identifies all annotations that are agreed by both annotators (i.e., which have exactly the same span and category) as the starting point for the final set of annotations. For each named entity (NE) category, a rule is then applied to determine how to handle disagreed annotations. Each rule consists of:

1) **Annotation selection strategy** (required)
   Two possible strategies may be specified:
   a) **Single** – For the NE category in question, only the disagreed annotations of a single, specified annotator (i.e., whoever is considered to be the better annotator) are added to the final set of annotations. All non-agreed annotations of the other annotator in the pair are ignored.
   b) **Combine** – Merge the disagreed annotations from both annotators.
      - In the case that disagreed annotations from each annotator have overlapping spans, the default behaviour of the algorithm is to retain the longer of the spans and to discard the shorter span. However, the rule may also specify that the *shorter* of the overlapping spans should be retained.
2) **Pattern-based filters** (optional)
   Any number of annotation span patterns may be specified; all annotations whose spans match one of these patterns will be excluded from the final set.

In many cases, disagreements occur simply because one of the annotators failed to spot and annotate a relevant word or phrase. This means that it is usually advantageous to choose the *combine* strategy for as many categories as possible, to maximise the number of annotations in the final set. However, if one annotator is clearly stronger than the other in annotating a particular category, then there is a danger that combining *all* disagreed annotations of both annotators could also result in the inclusion of erroneous annotations in the final set. If there are too many erroneous annotations, then it is likely that ML models that are trained using the corpus will become confused and may perform poorly when applied to the task of predicting NEs in new documents. However, we have observed that the errors made by a particular annotator within a given category often follow regular patterns. The purpose of the pattern-based filters is to allow erroneous annotations that follow such patterns to be excluded from

the final set, while still allowing the annotator's more correct annotations to be included in the final set.

As an example of a case in which pattern-based filters can be useful, some annotators marked up mentions of certain types of pumps (e.g., *personal sampling pump*) as *Sample Type Personal*, even though the guidelines explicitly state that any mentions of equipment or apparatus are out of scope for this category. Accordingly, a pattern-based filter matching *Sample Type Personal* annotations that include the word *pump* was helpful in excluding erroneous annotations. The ability to specify such filters means that the *single* annotation strategy only needs to be selected when one annotator in a pair has clearly failed to grasp multiple aspects of annotating a given category, and when their errors follow no regular pattern. In such cases, the inclusion of their annotations in the final set would significantly compromise its quality.

The option to specify that the *shorter* of two disagreed, but overlapping, spans should be added to the final annotation set is intended for application in cases where one of the annotators consistently includes too much information in their annotated spans. As an example, one annotator consistently included information about the *means* used to perform job tasks in their *Job Task/Activity* annotations (where this information was present), even though the guidelines state that this information should be excluded from annotated spans. For instance, the annotator selected the span *cutting of wood* <u>*with chain and circular saws*</u>, while the other annotator in the pair chose the more correct span *cutting of wood*. Accordingly, by specifying in the rule for *Job Task/Activity* that the shorter of any disagreed overlapping spans should be added to the final annotation set, greater compliance with the guidelines was ensured.

Evaluation of rule-based approach

Our rule-based method aims to allow full advantage to be taken of the different sets of annotations available for each article. By taking into account the relative strengths and weaknesses of different annotators, the aim of the rules is produce a final set of annotations that complies with the guidelines as closely as possible. As such, it is expected that following the application of the rules, the annotations across the corpus should exhibit a certain degree of homogeneity, and that this homogeneity should greater than if a simpler approach to selecting annotations was used. For example, the application of "best annotator" approach, which uses only the annotations of a single annotator who is perceived to be the better annotator of a pair, may result in potentially valuable annotations of the other annotator being discarded. These discarded annotations may sometimes comply equally well (or potentially even better) with the guidelines, compared to those produced by the overall "best" annotator.

To evaluate the success of our rule-based approach, we considered a specific characteristic of annotations belonging to each NE category, i.e., their average length. We investigated variations in the differences between the average lengths of NEs in articles covering our two selected exposure substances, i.e., diesel exhaust and respirable crystalline silica (RCS), depending on whether the "best annotator" or rule-based approach is applied. The results are reported in Table S2.1.

Table S2.1. Comparison of average annotation lengths (number of words) obtained using different approaches for final annotation selection.

Category	Annotation selection method					
	**Best Annotator**			**Rules**		
	**Diesel exhaust**	**RCS**	**Difference in av. length**	**Diesel exhaust**	**RCS**	**Difference in av. length**
Industry/Workplace	1.67	2.08	0.41	1.77	2.07	**0.30**
Job Task/Activity	2.23	2.76	0.53	2.54	2.43	**0.11**
OH Measurement Device	2.06	2.21	0.15	2.34	2.23	**0.11**
Occupation/ Job Title	1.73	1.97	0.24	1.73	1.97	0.24
Sample Type Personal	2.06	2.63	**0.57**	2.27	2.85	0.58
Substance or Exposure Measured	1.44	1.51	0.07	1.49	1.51	**0.02**

Bold underlined font is used to denote the approach resulting in the smallest differences in average annotation lengths between articles concerning diesel exhaust and RCS exposures

According to the results shown in Table S2.1, we consider that the rule-based approach is the more successful of the two methods for creating an optimal annotation set. This is because its application almost always results in the smallest difference between the average annotation lengths for a given NE category in articles covering the two different types of exposures. These results provide evidence that the application of the rule-based method results in the selection of a final set of annotations that adhere most closely and consistently to the annotation span guidelines, regardless of the exposure substance in question.

Overall, the more homogeneous the characteristics of annotations belonging to a particular NE category are across the complete corpus, the more likely it is that trained ML models will be sufficiently general to allow successful recognition of NEs relating to multiple types of substance exposures. It should, however, be noted that, regardless of the efficacy of the annotation selection method applied, the average lengths of annotations concerning different exposure substances cannot be expected to be identical. This is because articles concerning different types of exposures are likely to introduce distinct types of concepts and/or distinct ways of describing these concepts. Nevertheless, the method of selecting a final set of annotations that results in the smallest difference between the lengths of annotations concerning diesel exhaust and RCS exposures is to be preferred.
