# Supplementary material for: Supporting the working life exposome: Annotating occupational exposure for enhanced literature search
Source: PLoS One. 2024 Aug 15;19(8):e0307844. doi: 10.1371/journal.pone.0307844 (PMC11326626; doi:10.1371/journal.pone.0307844)
Supplement: S3 Appendix — (PDF) [file pone.0307844.s003.pdf]

# S3 Appendix: Detailed annotation analysis

This supplementary document complements the general discussion of the annotation results provided in the main manuscript with a more detailed analysis of the annotation results for individual categories. To make this discussion self-contained, we duplicate here two of the figures included in the main manuscript, and supplement them with two additional figures that allow for more in-depth analysis of the impact of changes made to the scope and span guidelines through various iterations of the annotation process.

- Fig S3.1 illustrates the *average* IAA rates attained among pairs of annotators for each category across each of the four workflow iterations. Separate results are shown for *exact* and *relaxed* matching criteria.
- Fig S3.2 provides box and whisker plots that illustrate the range of category-wise IAA rates obtained for each possible pairing of the annotators in different annotation rounds, to allow a more detailed analysis of performance variability among individual annotators.
- Fig S3.3 provides box and whisker plots that show the range of average span lengths marked up by different annotators for each NE category in different annotation rounds. Comparing the differences across the two rounds helps to assess the impact of changes made to *span* guidelines.
- Fig S3.4 provides box and whisker plots that depict the range of average frequencies of each NE category marked up by different annotators in different annotation rounds. Comparing these across different rounds helps to assess the impact of changes made to *scope* guidelines.

While Fig S3.1 reports statistics for all four rounds of annotation, Figs S3.2, S3.3 and S3.4 only provide information for rounds 1 and 3. Since these figures focus on differences among individual annotators, we chose these rounds because they both involved the annotation of a common set of articles by all annotators, thus allowing meaningful comparisons to be made.

It should be noted that since the characteristics of the annotations belonging to each different NE category are distinct in terms of aspects such as specific semantics and typical syntactic structures, variations in average lengths and frequencies *between* categories are to be expected. However, guideline updates should ideally result in a reduction in differences among annotators *within* each NE category.

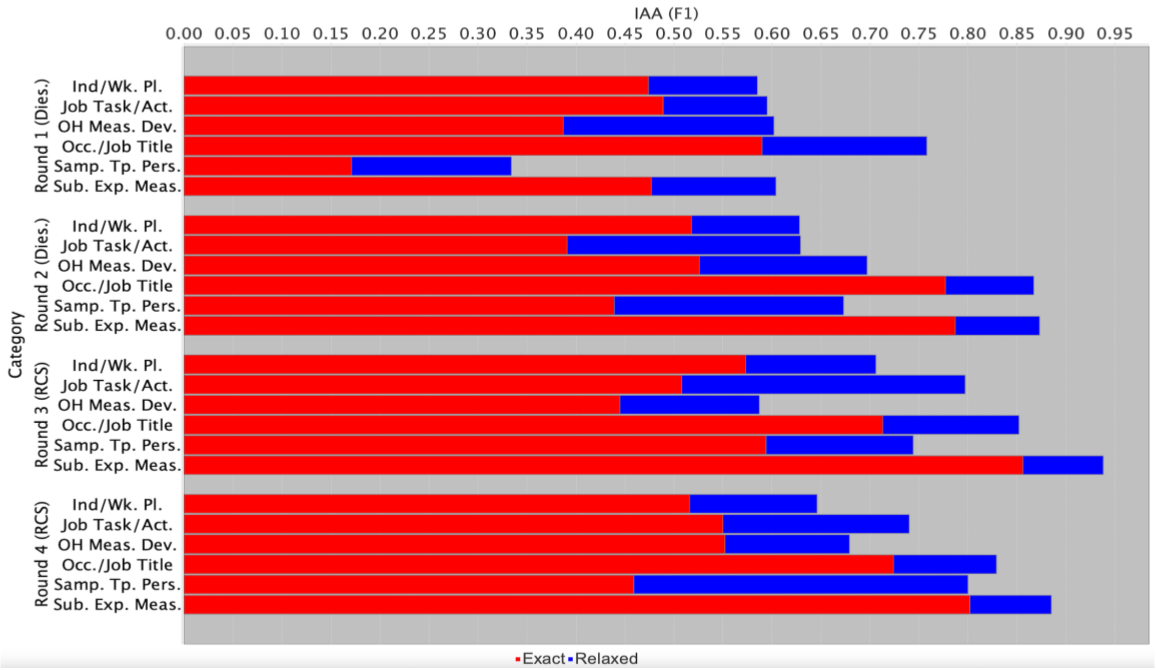


**Fig S3.1. Average exact and relaxed agreement rates in different annotation rounds.** "Ind/Wk. Pl" = Industry/Workplace; "Job Task/Act." = Job Task/Activity; "OH Meas. Dev." = OH Measurement Device; "Occ./Job Title" = Occupation/Job Title; "Samp. Tp. Pers" = Sample Type Personal; "Sub. Exp. Meas" = Substance or Exposure Measured.

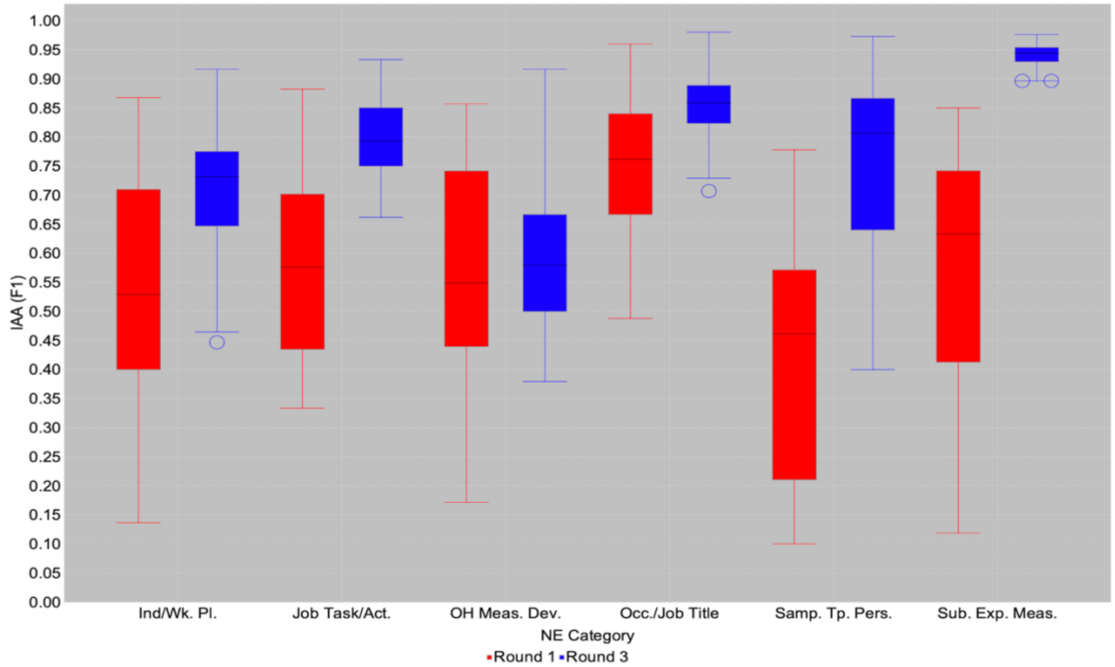


**Fig S3.2. Variability in pairwise relaxed IAA rates in rounds 1 and 3.** "Ind/Wk. Pl" = Industry/Workplace; "Job Task/Act." = Job Task/Activity; "OH Meas. Dev." = OH Measurement Device; "Occ./Job Title" = Occupation/Job Title; "Samp. Tp. Pers" = Sample Type Personal; "Sub. Exp. Meas" = Substance or Exposure Measured.

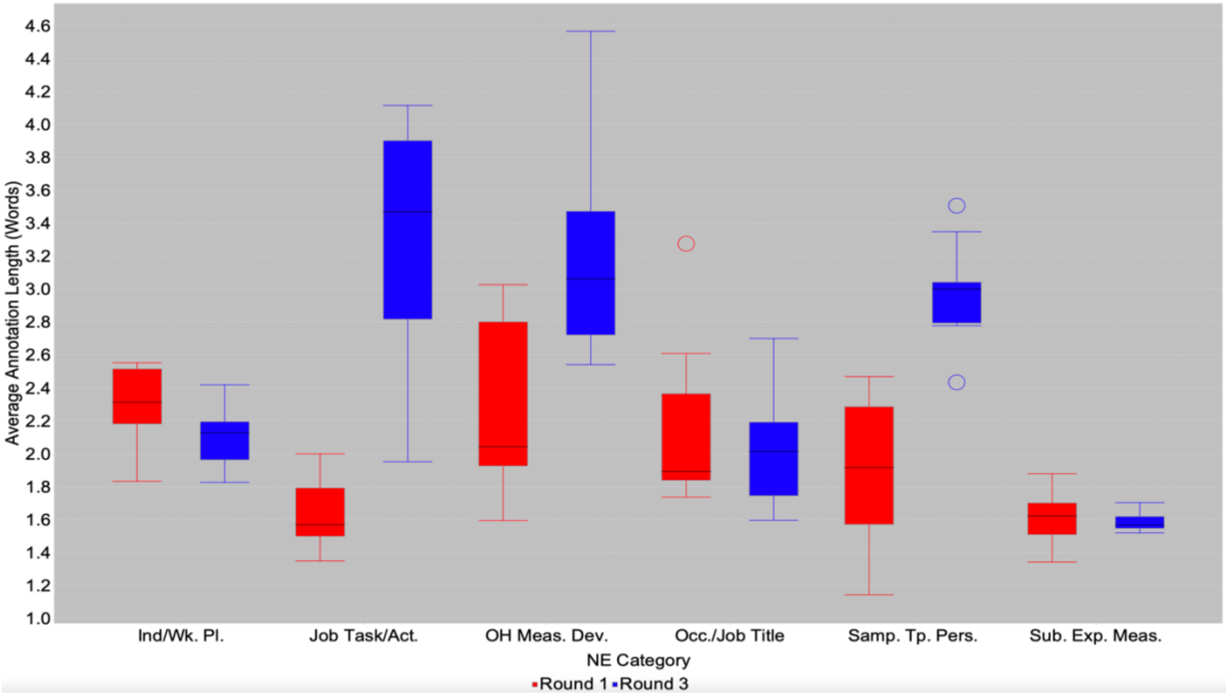


**Fig S3.3. Variability in average annotation lengths in rounds 1 and 3.** "Ind/Wk. Pl" = Industry/Workplace; "Job Task/Act." = Job Task/Activity; "OH Meas. Dev." = OH Measurement Device; "Occ./Job Title" = Occupation/Job Title; "Samp. Tp. Pers" = Sample Type Personal; "Sub. Exp. Meas" = Substance or Exposure Measured.

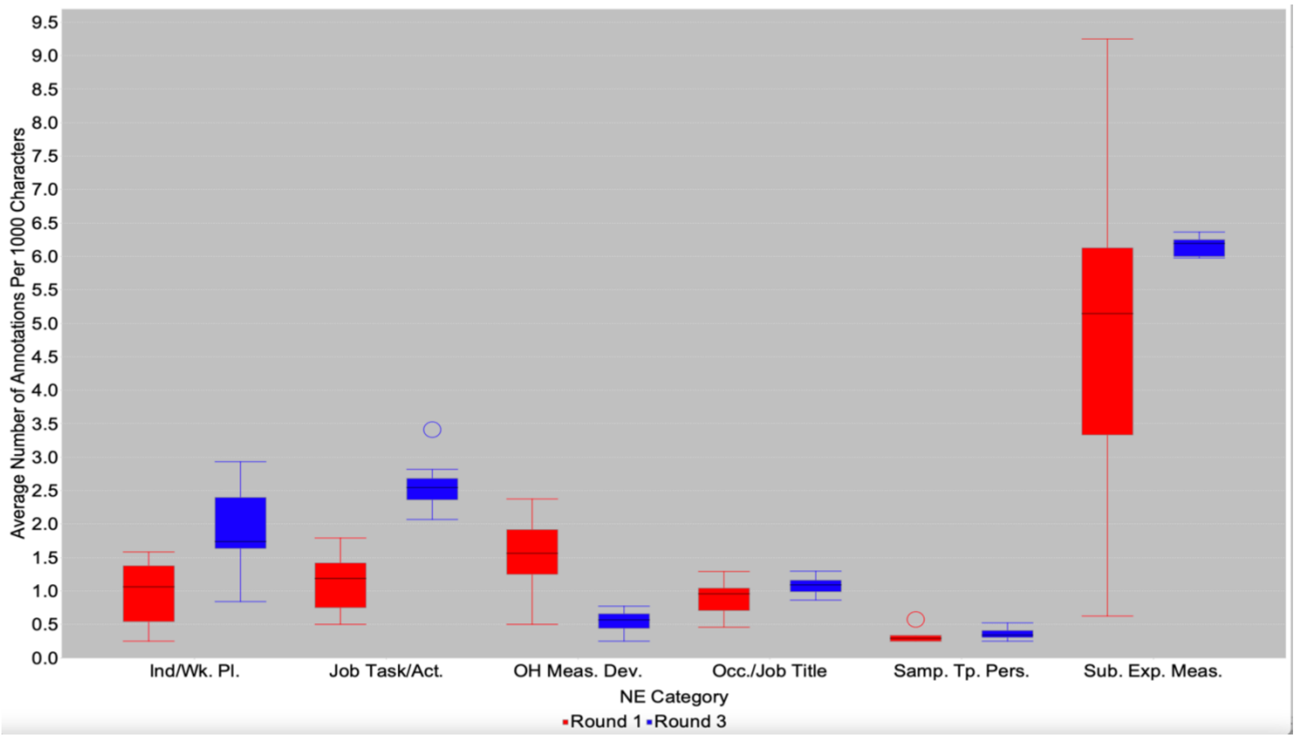


**Figure S3.4. Variability in average annotation frequencies per 1000 characters in rounds 1 and 3.** "Ind/Wk. Pl" = Industry/Workplace; "Job Task/Act." = Job Task/Activity; "OH Meas. Dev." = OH Measurement Device; "Occ./Job Title" = Occupation/Job Title; "Samp. Tp. Pers" = Sample Type Personal; "Sub. Exp. Meas" = Substance or Exposure Measured.

In the following sections, we separately discuss the results obtained and challenges faced for each of the six NE categories annotated.

## Substance or Exposure Measured

Fig S3.1 illustrates relatively low average IAA in round 1, while the long box plots in Figs S3.2 and S3.4 indicate significant divergences in annotator behaviour for this category in round 1. It was found that issues largely concerned disagreements involving another category that we decided to remove from the original annotation scheme, i.e., *Exposure Form*, which was defined as "*The physical state or form of the substance, chemical or pollutant to which exposure occurs*". Information about state or form is often expressed as an adjective that occurs in the same noun phrase as the substance (e.g., *particulate* or *gaseous*). However, a general guideline states that annotations should usually correspond to *complete* noun phrases. This resulted in many confusions about how to annotate phrases containing *both* a form *and* a substance, e.g., *gaseous PAHs.* To alleviate this potential for confusion, we decided to combine the two categories, such that if information about state or form is mentioned within the context of an exposure substance, then it should be included within the span of the *Substance or Exposure Measured* annotation. Following this change, Fig S3.1 shows that high average levels of agreement were achieved in all subsequent rounds. Furthermore, Figs S3.2 and S3.4 demonstrate that the removal of scope confusions with the original *Exposure Form* category resulted in a dramatically clearer understanding among all annotators about when to annotate instances of this category in round 3. Specifically, Fig S3.2 shows that in round 3, almost all annotator pairs achieved a relaxed IAA rate of 0.90 F1 or higher, and that some pairs attained an extremely high agreement of 0.98 F1. Meanwhile, Fig S3.4 illustrates that in contrast to the significant variation in the frequencies of annotation of this category among different annotators in round 1, all annotators identified similar numbers of *Substance or Exposure Measured* annotations in round 3.

Fig S3.3 demonstrates that, in comparison to most other categories, there are fairly small differences among annotators regarding the most appropriate span lengths to annotate for NEs in this category. This can be explained by the simple noun phrase structure of the majority of *Substance or Exposure Measured* annotations. Nevertheless, span disagreements were sometimes found to occur when unexpected phrase structures were encountered. For example, while information about the form as a substance typically occurs within the same noun phrase as the substance (e.g., *respirable crystalline silica*), it can sometimes occur after the noun phrase that introduces the substance, e.g., *particulate matter less than or equal to 10 μm diameter.* Analysis of such span disagreements resulted in updates being made to the guidelines to provide additional exemplification of the different ways in which information about the state/form of substances can be specified in articles. The positive effects of these guideline updates are evident in Fig S3.3, which illustrates a noticeable reduction in the variability of average span lengths for this category between rounds 1 and 3.

## Occupation/Job Title

In round 1, *Occupation/Job Title* exhibited the highest average IAA levels of all categories (see Fig S3.1), demonstrating that the scope and span guidelines were already well understood by several annotators. Nevertheless, Fig S3.2 reveals that was considerable variation in individual

annotator behaviour for this category in round 1. Analysis of discrepancies revealed that this was largely due to frequent category confusions between *Occupation/Job Title, Industry/Workplace* and *Job Task/Activity*. Since workers are often characterised in terms of the industry in which they work or the types of tasks that they typically undertake, phrases corresponding to occupations or job titles sometimes differ only subtly from those describing industries (*asphalt worker* vs *asphalt work*) or job tasks/activities (*asphalt stripper* vs. *asphalt stripping*). Based on the types of confusions identified, appropriate clarifications and adjustments were made to the guidelines at the end of round 1. Fig S3.1 shows that, as a result of these guideline changes, average IAA rates increased considerably in round 2, and remained relatively stable in subsequent rounds. Evidence that these positive effects of the guidelines extended to all annotators is provided in Fig S3.2. Specifically, the lower bound of pairwise IAA rose from around 0.47 F1 in round 1 to almost 0.70 F1 in round 3, while the upper bound reached almost 0.98 F1 in round 3. Furthermore, the degree of variability in pairwise IAA rates reduced considerably in round 3, demonstrating an increasingly shared understanding of the scope of this category among annotators.

Despite these positive trends, there are several indications that *Occupation /Job Title* is more challenging to annotate than *Substance or Exposure Measured*. Firstly, Fig S3.1 shows that from round 2 onwards, average IAA for *Occupation/Job Title* is always lower than *Substance or Exposure Measured*, while Fig S3.2 illustrates greater degree variability among pairwise IAA rates for *Occupation/Job Title*. Finally, Fig S3.3 indicates a certain level of variability among annotators regarding the most appropriate span lengths to annotate for *Occupation/Job Title* annotations, which the updates to the guidelines between rounds 1 and 3 failed to resolve.

In round 1, a contributing factor towards IAA issues concerned different opinions about whether or not to annotate general phrases that can encompass a range of possible occupations, e.g., *underground construction workers*. This term could refer to workers with various more specific occupational titles, such as *coal miner* or *power loader*. Such general phrases, which often characterise people according to the industry in which they work or the tasks that they undertake as part of their job (e.g., *drill and blast crew),* tend to be longer than simple job titles like *electrician* or *carpenter*. The uncertainly about whether to annotate these longer, more general phrases at least in part explains the variability in average span lengths shown in Fig S3.3. To help to reduce such confusions, the scope guidelines were updated to specify that *all* phrases that characterise a person or group of people in terms of what they do for a living should be annotated, regardless of whether this characterisation is in terms of the industry that they work in, the tasks that they undertake or specific occupational titles.

A further issue with respect to *Occupation/Job Title* concerns the range of potentially complex phrases that can characterise workers, which have come to light through the various rounds of annotation. Confusions and uncertainties regarding how and whether to annotate a number of different types of phrases contributed towards the continuing IAA variability (Fig S3.2), average span length variability (Fig S3.3) and slight drop in average IAA in round 3 (Fig S3.1). Table S3.1 provides several examples of how information in simple noun phrases that describe workers can also be conveyed by more complex phrase types. Although we have tried to cover these cases in discussions and by expanding the guidelines, they inevitably add complexity to the annotation task, in terms of both trying to spot such phrases and deciding on the most appropriate spans to annotate.

**Table S3.1 Examples of job descriptions expressed by simple and more complex phrases.**

| Simple Noun Phrase | Modified Noun Phrase with (near) equivalent meaning | Modifying phrase type |
|---|---|---|
| *train drivers* | *drivers of trains* | Prepositional Phrase |
| *toll station workers* | *workers in toll stations* | Prepositional Phrase |
| *backhoe operator* | *operator of the backhoe* | Prepositional Phrase |
| *farm workers* | *workers on the farms* | Prepositional Phrase |
| *asphalt mill operator* | *workers operating asphalt mills* | "ing" clause |
| *grinder operator* | *worker using the grinder* | "ing" clause |
| *SiC processors* | *workers processing SiC* | "ing" clause |
| *SiC production workers* | *workers who are employed in the production of SiC* | Relative clause |
| *SiC production workers* | *workers involved in the production of SiC* | Reduced relative clause |

***Prepositional phrase*** - a group of words that begins with a preposition and ends with a noun/noun phrase
***Relative clause*** - a clause used to give additional information about a noun, introduced by a relative pronoun like 'that', 'which' or 'who'.
***Reduced relative clause*** – a shortened version of a relative clause, in which the pronoun is omitted.

## Industry/Workplace

This category has a wide semantic scope, which encompasses phrases that refer either to industries *or* specific workplaces. An analysis of annotator discrepancies revealed that there is generally good agreement for phrases referring to industries or sectors. However, achieving consensus about the scope of *workplaces* that should be annotated has been rather more problematic. There is a potentially large range of phrases providing information about where people work, for instance:

- Indoor work areas, e.g., *diesel use mines, tollbooths*
- Outdoor work areas, e.g., *four-lane motorway, heavy repair area*
- Vehicles driven by workers, e.g., *diesel fork-lift truck, lead locomotive*
- Large pieces of heavy equipment (underlined), e.g., *The operator spent almost all of his time on top of the asphalt mill, a laborer is seen stationed at the pneumatic drills.*
- Phrases describing specific features of the working environment, e.g., *surface, underground* and *enclosed workspaces*

The initial guidelines provided only a very small number of workplace examples, which led to divergent decisions among annotators, as illustrated by the highly variable pairwise IAA results show in Fig S3.2 for this category in round 1. The full diversity of the types of workplaces that can be mentioned in articles did not become fully apparent until we had collected evidence from the large-scale annotation effort in round 2. Based on this evidence, we substantially updated the scope guidelines for *Industry/Workplace* prior to round 3, by categorising,

enumerating and exemplifying a range of different types of workplaces that should or should not be annotated. It was determined that all of the types of workplaces enumerated above can provide potentially useful information about the characteristics of working environments that can impact upon exposures, and hence should be annotated. More general environmental descriptions, e.g., *city* or *urban*, and very general references to places of work, such as *work site* or *plant*, were specified to be excluded, since they provide little useful information.

Evidence of the positive impact of these guideline changes can be seen in several of the figures. For example, Fig S3.1 shows that both exact and relaxed matching rates increased noticeably in round 3. Meanwhile, Fig S3.2 confirms that this impact extends across all annotators, with a shift towards higher pairwise IAA rates among all annotators in round 3. Furthermore, Fig S3.4 illustrates a trend towards increased annotation frequency between rounds 1 and 3, which indicates that many annotators began to grasp the wide range of phrases that can fall under this category.

Despite these positive impacts, the degree of IAA variability among annotator pairs in round 3 for *Industry/Workplace* (Fig S3.2) is still quite high compared to some other categories. Moreover, the variability for this category in terms of annotation frequencies in round 3 (Fig S3.4) is the highest among categories. These observations are indicative of the difficulties faced in trying to define a general set of guidelines for *Industry/Workplace* that is applicable in a wide range of scenarios. Every new article generally introduces new types of workplaces, which can lead to challenging questions and decisions, some of which may be reliant on the annotator's depth of knowledge about a particular industry, e.g.:

- *Is "farm" too general to annotate as a workplace*?
- *What type of equipment is a "potato sorting machine" or a "backhoe"*? *Are they sufficiently large to be annotated as workplaces?* [Only heavy equipment should be annotated as a workplace]
- *How specific should an annotation span be, e.g., is "crusher" sufficient or should "upper platform of the crusher" be annotated?*

## Job Task/Activity

Similarly to *Industry/Workplace*, determining the exact scope of this category is challenging; there is large range of words/phrases that could be considered as *potentially* suitable for annotation, and deciding which of these constitute suitable NEs can be problematic. Moreover, *Job Task/Activity* presents further challenges due to its syntactic diversity, i.e., annotation may focus on *either* noun *or* verbs. Such complexities, combined with an initially small set of guidelines, help to explain why the average relaxed IAA rate (see Fig S3.1) was relatively low.

Fig S3.1 also reveals that in round 2, average exact IAA for *Job Task/Activity* was 50% lower than average relaxed IAA, and constituted the only IAA statistic among all categories that declined between rounds 1 and 2. This indicates that annotators faced significant difficulties in choosing appropriate spans for *Job Task/Activity* annotations. The problem stems from the fact that descriptions of activities can vary in their complexity. The scale of this complexity (and the potential for divergent annotation decisions regarding how much information to annotate) only became fully apparent after the large-scale annotation in round 2. While descriptions of activities sometimes correspond only to single words like *weld* or *drilling*, they may also include, among other things:

- Further details about the activity itself, e.g., *spraying* <u>*of oil onto concrete forms*</u>

- The location of the activity, e.g., *abrasive blasting of the steel structure* *under a bridge*;
- The means of carrying out the task, e.g., *fastening of unsafe rock with steel bolts*

As a further complication, activities may be described using the passive as well as the active voice. For example, the activity of *sawing expansion joints in the fresh concrete* may also be conveyed using the passive construction *expansion joints were being sawn in the fresh concrete.*

To address scope uncertainties, we considered in more detail which types of phrases convey useful information. Some simple nouns/verbs that had been annotated in round 2 did not convey specific work activities (e.g., *walk*), while others were too vague to provide any useful information about the specific type of activity being undertaken, e.g., *pouring, installation, laying,* etc. However, words conveying vague activities become considerably more meaningful if further detail is provided in their immediate context, e.g., *concrete pouring, installation of drop ceilings, laying conduit.* In other cases, a verb or noun alone is sufficient to provide an idea of the specific activity, e.g., *welding* or *drilling.* Nevertheless, further information can be useful, if it is provided in the context of such verbs or nouns, e.g., *rock drilling.* Based on these observations, we determined that:

- Single noun or verbs should *only* be annotated if they convey a specific, well-defined activity. Vague nouns/verbs may *only* be annotated if further details that clarify the nature of the activity are provided in the neighbouring content.
- Additional details about activities should *always* be included in the span, when they are provided in the immediate context of the noun or verb, and when these details comply with certain rules (see below).

The rules in question place restrictions on both the internal syntactic structure and semantics of annotated phrases, to try to ensure that they convey useful information about the activity, while also being feasible to annotate and to recognise automatically. To fulfil the latter criterion, annotated spans should ideally have consistent and predictable structures, and avoid being overly long.

In terms of restrictions on the *syntactic* structure of annotations, we specify that an annotated span may consist ONLY of the following:

- A noun or verb that describes the basic activity PLUS:
    - Other words within the same syntactic phrase, e.g., noun phrases may include other nouns and/or adjectives that contribute towards the description of the activity (e.g., *rock drilling*).
    - One or more phrases that immediately follow the basic activity phrase, i.e., either grammatical objects of activities described using verbs (e.g., *laying conduit*), and/or prepositional phrases (e.g., *installation of drop ceilings*).
    - The grammatical subject of an activity described using a verb, if the activity is described using the passive voice (e.g., *concrete was being poured*).

In terms of *semantic* restrictions, information conveyed by any parts of the annotated span that extend beyond the basic activity word must correspond to one of the types of information exemplified in the *Key* of Fig S3.5. The figure provides examples of how these different types of information can be expressed using several different types of phrases and structures.

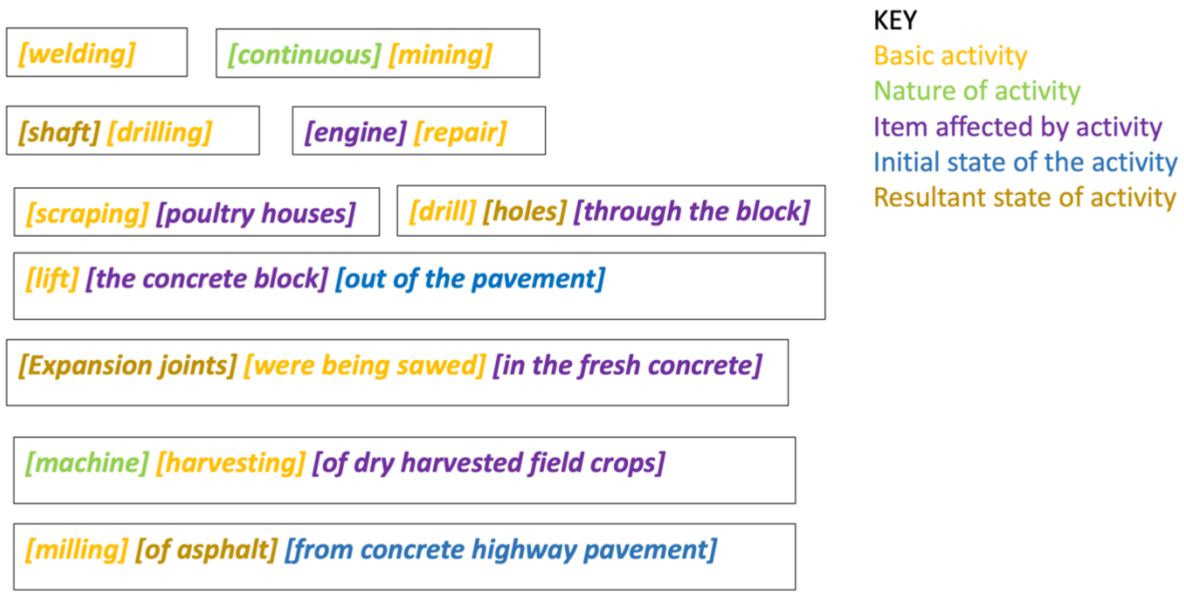


**Fig S3.5. Examples of *Job Task/Activity* spans with varying structures and semantics**

The success of introducing the detailed rules and criteria outlined above into the guidelines prior to round 3 may be evidenced in several ways. In Fig S3.1, the significant increase in average relaxed IAA observable in round 3 demonstrates an enhanced general understanding of the scope of the category. Fig S3.2 confirms that this positive effect extended to all annotators: there was a considerable reduction in the variability of pairwise relaxed IAA rates in round 3, with the lowest IAA rate doubling from 0.33 F1 in round 1 to 0.66 F1 in round 3. Furthermore, almost half of annotator pairs achieved an F1 of 0.80 or above in round 3. In Fig S3.4, the large increase in annotation frequencies in round 3, together with the reduced variability among annotators in terms of this statistic, confirm a better shared appreciation of the diversity of phrases that can be annotated in this category.

In terms of improved span agreement, Fig S3.1 illustrates a round-on-round rise in average exact matching IAA rates from round 2 onwards. Furthermore, Fig S3.3 illustrates that the average lengths of annotations increased between rounds 1 and 3, which confirms that annotators generally begun to understand that *Job Task/Activity* annotations should capture details that go beyond a simple noun or verb, whenever possible. Nevertheless, Fig S3.3 shows that extensions to the guidelines resulted in increased variability among annotators in round 3 regarding the best length of span to annotate. To take a particular example, one annotator simply marked up the verb *sawing,* while another annotated the much longer phrase *sawing under wet conditions with a hand-held Still saw in wet weather*. A possible reason for such disagreements is that some annotators may struggle to fully grasp and absorb the involved nature of the guidelines for this category which, at ten pages, are the most detailed of all categories. Although the longer annotation complies with the *syntactic* restrictions for this category, in that an annotation may consist of a verb followed by one or more prepositional phrases, it flouts the guideline stating that phrases describing tools used to carry out the work should be excluded from annotated spans. Furthermore, the guidelines do not provide explicit mention of how to handle phrases dealing with climatic conditions (e.g., *under wet conditions*) which could add to the confusion.

## Occupational Hygiene (OH) Measurement Device

Various types of scope disagreements were initially found to occur with this category. They included uncertainty about whether to annotate only names of devices (e.g., *P-Trak*) or whether to additionally mark up more descriptive phrases (e.g., *condensation particle counter*). Furthermore, while annotations in this category are supposed to correspond to *only* to devices used to measure levels of particulate and gaseous exposures in the workplace, some annotators were also incorrectly marking up analytical methods (e.g., *chromatography*). However, the most problematic aspect of this category for several annotators appears to be grasping that its scope covers only a *subset* of the devices and apparatus that are likely to be mentioned in the context of occupational exposure studies. Some annotators were found to be erroneously marking up almost *all* mentions of equipment and apparatus, including equipment that is used to perform laboratory analysis (e.g., *flame ionization detector*), pieces of apparatus that form part of the "sampling train" (e.g., *NIOSH-approved pump*) and filters on which samples are collected (e.g., a*crylic copolymer membrane filters*). It was found that particular difficulties arose in trying to differentiate between mentions of various types of tubes, some of which form part of the sampling train and so should NOT be annotated (e.g., *silicone rubber tubes*, which are used to connect a pump to sampling head), and others which are used to collect gaseous samples (e.g., *Dräger stain tubes*) and so SHOULD be annotated.

We updated the guidelines in several ways to try to make the task clearer. These included separately enumerating and exemplifying different types of apparatus/equipment that *should* be annotated in the scope guidelines (e.g., sampling heads, cassettes and other filter holders, containers for collecting gaseous samples, devices used to collect real time samples); trying to differentiate between similar types of phrases that *should* or *should not* be annotated (e.g., phrases that include the word *tubes*); and extending the span guidelines to better enumerate the types of words that should be included within spans (e.g. manufacturer names, information about the size or construction material of apparatus, etc).

As shown in Fig S3.1, these guideline updates initially appeared to have a positive impact, since there was a fairly significant increase in the relaxed matching rate between rounds 1 and 2, rising from 0.52 to 0.70 F1. However, in rounds 3 and 4, average IAA was unstable. While the extended scope guidelines have largely eliminated annotations corresponding to analytical *methods*, it appears that, more than other categories, grasping the precise semantic scope of this category requires an in-depth knowledge of occupational hygiene monitoring equipment. Nevertheless, other figures provide further evidence of the positive impacts of the guideline changes. For example, Fig S3.3 demonstrates a general trend towards an increased length of annotated spans, as encouraged by the updated span guidelines. Meanwhile, a significant decrease in annotation frequency between rounds 1 and 3 is observable in Fig S3.4. This indicates that annotators began to gain an increased appreciation of the restricted semantic scope of this category.

Fig S3.2 illustrates a considerable increase in the lower bound of pairwise IAA between rounds 1 and 3, while the upper bound of IAA reaches as high as 0.92 F1 in round 3. This indicates that the guidelines are sufficiently clear and detailed to support high quality annotation of this category, as long as annotators possess appropriate background knowledge. Nevertheless, the difference between these upper and lower bounds in round 3 remains greater than all other categories apart from *Sample Type Personal*. This shows that there remain many differences of opinion regarding the exact semantic scope of this category. Mentions of equipment that are similar to those exemplified in the guidelines appear to be straightforward for all annotators to

spot, e.g., those including words such as *cyclone, sampler, monitor* or *cassette.* However, mentions that differ more significantly from the guideline examples are missed by some annotators, e.g., *Type 1000 MLE Personal DataRAM* or *SidePak AM10.* Furthermore, there remains some uncertainly regarding span length, e.g., whether to mark-up *DustTrak* or *DustTrak Aerosol Monitor.*

## Sample Type Personal

In round 1, the original scope of this category encompassed phrases providing evidence of both personal *and* static/stationary samples. However, evidence of the latter appears to occur relatively infrequently, and is often provided in the form of phrases that specify the *location* of the sampling equipment, which can be long and complex (e.g., *Air was sampled at a position where portions of newly produced asphalt were emptied repeatedly*). Such phrases are difficult to annotate consistently, and ML models, in general, struggle to learn to detect phrases with such characteristics, especially when there is sparse evidence about them. Furthermore, since location-denoting phrases are also within the scope of *Industry/Workplace,* there was some confusion among annotators about how to label such phrases. In contrast, phrases denoting that the samples were personal in nature tend to be shorter and far less variable (e.g., *personal sampling*) and thus easier to annotate and to recognise automatically. According to such issues, and also because personal sampling is more relevant for JEM development, the scope of the category was reduced from round 2 onwards to cover only phrases providing evidence of personal sampling.

The reduction in scope and resulted in a large increase in relaxed IAA between rounds 1 and 2, as may be observed Fig S3.1. Additional updates to the guidelines have aimed to further reduce the complexity of this category, e.g., by excluding mentions of personal sampling equipment, which were sometimes confused with *OH Measurement Device*, and by limiting the scope of three basic criteria. i.e.:

- Mentions that personal sampling/measurement techniques were carried out as part of methodological descriptions of the study, e.g., *Personal sampling was performed using GSP samplers*.
- Mentions of personal samples/measurements that are reported as results, e.g., *Neither respirable quartz nor respirable dust were detected by the 127-minute personal breathing zone sample*.
- Phrases denoting that sampling was carried out on or near the workers' bodies, e.g., *Sampling was performed in the breathing zone of the workers.*

The use of such a simple and straightforward set of scope inclusion criteria and the clarification of confusing cases resulted in a round-on-round improvement of average relaxed IAA, reaching 0.80 F1 in round 4 (see Fig S3.1). Fig S3.4 also illustrates that there is little variability in the frequencies of annotations of this category created by different annotators. Furthermore, Figure S3.2 shows that in round 3, half of the annotator pairs achieved relaxed IAA in the range of 0.81 – 0.97 F1. Although there remains considerable variation in pairwise relaxed IAA in round 3 for the other half of the annotator pairs, this may at least in part be explained by the low number of annotations, compared to other categories. This means that even small differences between annotators can result in large differences in agreement figures.

For all rounds of annotation, exact IAA rates fall some way behind the relaxed IAA rates. Initially, there was confusion about whether to annotate multi-word phrases such as *personal*

*full-shift samples*, or whether to simply annotate the word *personal*. To encourage consistent decisions in such cases, we introduced a guideline stating that, whenever a noun phrase denotes personal measurements, sampling or exposures, the complete phrase should be annotated. The positive impact of this guideline may be observed in Fig S3.3, which shows that the average length of annotations in this category considerably increased between rounds 1 and 3. This provides evidence that in the latter round, more complete phrases were being annotated. Furthermore, there was a reduction in the variability of average span lengths among different annotators, which indicates that the “whole phrase” rule resulted in less confusion regarding the most appropriate spans to annotate. Despite this, Fig S3.1 shows, even in round 4, there remains a considerable discrepancy between exact and relaxed matching rates. A possible reason is the paucity of supporting examples in the guidelines. For the more frequently occurring NE categories, we often include several supporting examples for each individual guideline. However, only a single supporting example is provided for some of the *Sample Type Personal* guidelines. When mentions of the *breathing zone* occur in articles, some annotators *only* annotate these two words, even if they occur as part of a larger phrase, since they correspond to one of the example phrases provided in the guidelines. In contrast, other annotators comply with the rule of annotating the whole phrase, e.g., *operator's breathing zone* or *quasi-breathing zone sample.* Such span-level issues may be helped by augmenting the set supporting examples provided for this category in the guidelines.
